# Supplementary material for: Meta-Analysis and Systematic Literature Review of the Genus Pneumocystis in Pet, Farm, Zoo, and Wild Mammal Species
Source: J Fungi (Basel). 2023 Nov 4;9(11):1081. doi: 10.3390/jof9111081 (PMC10672670; doi:10.3390/jof9111081)
Supplement: Supplementary file 1 [file jof-09-01081-s001.zip › Table S2_v2.pdf]

Table S2. List of studies excluded from the individual data sets grouped by reason of exclusion.

1. **Prevalence.**

Sample sizes ≤ 10 samples (n = 324)

Artiodactyla	Antilope cervicapra	Weissenbacher-Lang et al. 2023	Detection of Pneumocystis and morphological description of fungal distribution and severity of infection in thirty-six mammal species
Artiodactyla	Antilope spp.	Meer and Brug 1942	Infection a Pneumocystis chez l'homme et chez les animaux
Artiodactyla	Bos bonasus	Weissenbacher-Lang et al. 2023	Detection of Pneumocystis and morphological description of fungal distribution and severity of infection in thirty-six mammal species
Artiodactyla	Bos bonasus	Meer and Brug 1942	Infection a Pneumocystis chez l'homme et chez les animaux
Artiodactyla	Bubalus bubalis	Weissenbacher-Lang et al. 2023	Detection of Pneumocystis and morphological description of fungal distribution and severity of infection in thirty-six mammal species
Artiodactyla	Capra hircus	Shimizu et al. 1985	Occurrence of Pneumocystis carinii in animals in Japan
Artiodactyla	Capra hircus	Settnes and Henriksen 1989	Pneumocystis carinii in large domestic animals in Denmark
Artiodactyla	Capra hircus	McConnell et al. 1971	Pneumocystosis in a domestic goat
Artiodactyla	Capra hircus	Shimizu et al. 1985	Occurrence of Pneumocystis carinii in animals in Japan
Artiodactyla	Capra hircus	Shimizu et al. 1985	Occurrence of Pneumocystis carinii in animals in Japan
Artiodactyla	Capra hircus	Sakakibara et al. 2013	Pneumocystis carinii Infection in a Domestic Goat (Capra hircus domesticus) with Multibacillary Paratuberculosis
Artiodactyla	Hippotragus niger	Wilson et al. 1974	Observations on mortality rates and disease in roan and sable antelope on nature reserves in the Transvaal
Artiodactyla	Ovis aries	Meer and Brug 1942	Infection a Pneumocystis chez l'homme et chez les animaux
Artiodactyla	Rupicapra rupicapra	Weissenbacher-Lang et al. 2023	Detection of Pneumocystis and morphological description of fungal distribution and severity of infection in thirty-six mammal species
Artiodactyla	Camelus bactrianus	Weissenbacher-Lang et al. 2023	Detection of Pneumocystis and morphological description of fungal distribution and severity of infection in thirty-six mammal species
Artiodactyla	Camelus dromedarius	Weissenbacher-Lang et al. 2023	Detection of Pneumocystis and morphological description of fungal distribution and severity of infection in thirty-six mammal species
Artiodactyla	Lama glama	Underwood et al. 1992	Apparent retrovirus-induced immunosuppression in a yearling llama

Artiodactyla	Lama glama	Weissenbacher-Lang et al. 2023	Detection of Pneumocystis and morphological description of fungal distribution and severity of infection in thirty-six mammal species
Artiodactyla	Capreolus capreolus	Settnes et al. 1986	Pneumocystis carinii Delanoe & Delanoe, 1912 found in lungs of freeliving animals in Denmark at Autopsy
Artiodactyla	Capreolus capreolus	Weissenbacher-Lang et al. 2023	Detection of Pneumocystis and morphological description of fungal distribution and severity of infection in thirty-six mammal species
Artiodactyla	Cervus alfredi	Weissenbacher-Lang et al. 2023	Detection of Pneumocystis and morphological description of fungal distribution and severity of infection in thirty-six mammal species
Artiodactyla	Cervus elaphus	Weissenbacher-Lang et al. 2023	Detection of Pneumocystis and morphological description of fungal distribution and severity of infection in thirty-six mammal species
Artiodactyla	Cervus nippon	Weissenbacher-Lang et al. 2023	Detection of Pneumocystis and morphological description of fungal distribution and severity of infection in thirty-six mammal species
Artiodactyla	Cervus spp.	Weissenbacher-Lang et al. 2023	Detection of Pneumocystis and morphological description of fungal distribution and severity of infection in thirty-six mammal species
Artiodactyla	Cervus spp.	Yoshida and Ikai 1979	Pneumocystis carinii pneumonia: epidemiology in Japan and cyst concentration method
Artiodactyla	Dama dama	Settnes and Henriksen 1989	Pneumocystis carinii in large domestic animals in Denmark
Artiodactyla	Elaphurus davidianus	Weissenbacher-Lang et al. 2023	Detection of Pneumocystis and morphological description of fungal distribution and severity of infection in thirty-six mammal species
Artiodactyla	Muntiacus muntjak	Meer and Brug 1942	Infection a Pneumocystis chez l'homme et chez les animaux
Artiodactyla	Rangifer tarandus	Weissenbacher-Lang et al. 2023	Detection of Pneumocystis and morphological description of fungal distribution and severity of infection in thirty-six mammal species
Artiodactyla	Rangifer tarandus groenlandicus	Settnes et al. 1986	Pneumocystis carinii Delanoe & Delanoe, 1912 found in lungs of freeliving animals in Denmark at Autopsy
Artiodactyla	Rusa unicolor	Meer and Brug 1942	Infection a Pneumocystis chez l'homme et chez les animaux
Artiodactyla	Sus scrofa	Yoshida and Ikai 1979	Pneumocystis carinii pneumonia: epidemiology in Japan and cyst concentration method
Artiodactyla	Sus scrofa domesticus	Seibold and Munnell 1977	Pneumocystis carinii in a pig
Artiodactyla	Sus scrofa domesticus	Ramos-Vara et al. 1998	Characterization of natural occuring Pneumocystis carinii pneumonia in pigs by histopathology, electron microscopy, in situ hybridization and PCR amplification
Artiodactyla	Sus scrofa domesticus	Shimizu et al. 1985	Occurrence of Pneumocystis carinii in animals in Japan

Artiodactyla	Sus scrofa domesticus	Jensen et al. 2001	Application of fluorescent in situ hybridization for specific diagnosis of Pneumocystis carinii pneumonia in foals and pigs
Carnivora	Ailurus fulgens	Poelma 1975	Pneumocystis carinii infections in zoo animals
Carnivora	Canis lupus	Weissenbacher-Lang et al. 2023	Detection of Pneumocystis and morphological description of fungal distribution and severity of infection in thirty-six mammal species
Carnivora	Canis lupus familiaris	Cabanes et al. 2000	Pneumocystis carinii pneumonia in a Yorkshire terrier dog
Carnivora	Canis lupus familiaris	English et al. 2001	DNA analysis of Pneumocystis infecting a Cavalier King Charles spaniel
Carnivora	Canis lupus familiaris	Fox 2012	A case of suspected Pneumocystis carinii pneumonia in a Cavalier King Charles spaniel
Carnivora	Canis lupus familiaris	Furuta et al. 1994	Spontaneous Pneumocystis carinii infection in the dog with naturally acquired generalised demodicosis
Carnivora	Canis lupus familiaris	Giannoulopoulos and Lobetti 2020	Pneumocystosis in a lurcher puppy
Carnivora	Canis lupus familiaris	Hagiwara et al. 2001	Pneumocystis carinii pneumonia in a Cavalier King Charles spaniel
Carnivora	Canis lupus familiaris	Kanemoto et al. 2015	Common variable immune deficiency in a Pomeranian with Pneumocystis carinii pneumonia
Carnivora	Canis lupus familiaris	McCully et al. 1979	Canine Pneumocystis pneumonia
Carnivora	Canis lupus familiaris	Okine et al. 2018	Diagnosis of Pneumocystis pneumonia in a 2-year-old King Charles Cavalier Spaniel using the polymerase chain reaction
Carnivora	Canis lupus familiaris	Sakashita et al. 2020	Disseminated pneumocystosis in a toy poodle
Carnivora	Canis lupus familiaris	Sedlmaier and Dahme 1955	Pneumocystis carinii infection in a dog
Carnivora	Canis lupus familiaris	Van den Akker and Goedbloed 1960	Pneumonia caused by Pneumocystis carinii in a dog
Carnivora	Canis lupus familiaris	Best et al. 2019	Confirmed case of Pneumocystis pneumonia in a Maltese Terrier × Papillon dog being treated with toceranib phosphate
Carnivora	Canis lupus familiaris	Merrill et al. 2021	X-linked CD40 ligand deficiency in a 1-year-old male Shih Tzu with secondary Pneumocystis pneumonia
Carnivora	Canis lupus familiaris	Botha and van Rensburg 1979	Pneumocystosis: a chronic respiratory distress syndrome in dog
Carnivora	Canis lupus familiaris	Meffert 2009	Pneumocystis pneumonia in two Cavalier King Charles spaniel littermates
Carnivora	Canis lupus familiaris	Ramsey et al. 1997	Pneumocystis carinii pneumonia in two Cavalier King Charles spaniels
Carnivora	Canis lupus familiaris	Copland 1974	Canine pneumonia caused by Pneumocystis carinii

Carnivora	Canis lupus familiaris	Farrow et al. 1972	Pneumocystis pneumonia in the dog
Carnivora	Canis lupus familiaris	Lobetti 2000	Common variable immunodeficiency in miniature dachshunds affected with pneumocystis carinii pneumonia
Carnivora	Canis lupus familiaris	Weissenbacher-Lang et al. 2017	Pneumocystis carinii infection with severe pneumomediastinum and lymph node involvement in a Whippet mixed-breed dog
Carnivora	Canis lupus familiaris	Johnson et al. 2023	Oculosystemic pneumocystosis in 2 sibling Chihuahuas
Carnivora	Canis lupus familiaris	Cisse et al. 2021	Genomic insights into the host specific adaptation the Pneumocystis genus
Carnivora	Canis lupus familiaris	Petini et al. 2019	Nested–polymerase chain reaction detection of Pneumocystis carinii f. sp. canisin a suspected immunocompromised Cavalier King Charles spaniel with multiple infections
Carnivora	Canis lupus lycaon	Weissenbacher-Lang et al. 2023	Detection of Pneumocystis and morphological description of fungal distribution and severity of infection in thirty-six mammal species
Carnivora	Vulpes vulpes	Weissenbacher-Lang et al. 2023	Detection of Pneumocystis and morphological description of fungal distribution and severity of infection in thirty-six mammal species
Carnivora	Vulpes vulpes	Yoshida and Ikai 1979	Pneumocystis carinii pneumonia: epidemiology in Japan and cyst concentration method
Carnivora	Vulpes vulpes	Meer and Brug 1942	Infection a Pneumocystis chez l'homme et chez les animaux
Carnivora	Vulpes zerda	Poelma 1975	Pneumocystis carinii infections in zoo animals
Carnivora	Felis catus	Yoshida and Ikai 1979	Pneumocystis carinii pneumonia: epidemiology in Japan and cyst concentration method
Carnivora	Felis silvestris	Weissenbacher-Lang et al. 2023	Detection of Pneumocystis and morphological description of fungal distribution and severity of infection in thirty-six mammal species
Carnivora	Lynx lynx	Weissenbacher-Lang et al. 2023	Detection of Pneumocystis and morphological description of fungal distribution and severity of infection in thirty-six mammal species
Carnivora	Panthera leo	Weissenbacher-Lang et al. 2023	Detection of Pneumocystis and morphological description of fungal distribution and severity of infection in thirty-six mammal species
Carnivora	Panthera pardus	Weissenbacher-Lang et al. 2023	Detection of Pneumocystis and morphological description of fungal distribution and severity of infection in thirty-six mammal species
Carnivora	Panthera tigris	Weissenbacher-Lang et al. 2023	Detection of Pneumocystis and morphological description of fungal distribution and severity of infection in thirty-six mammal species

Carnivora	Puma concolor	Weissenbacher-Lang et al. 2023	Detection of Pneumocystis and morphological description of fungal distribution and severity of infection in thirty-six mammal species
Carnivora	Puma concolor	Meer and Brug 1942	Infection a Pneumocystis chez l'homme et chez les animaux
Carnivora	Aonyx cinereus	Weissenbacher-Lang et al. 2023	Detection of Pneumocystis and morphological description of fungal distribution and severity of infection in thirty-six mammal species
Carnivora	Lontra canadensis	Weissenbacher-Lang et al. 2023	Detection of Pneumocystis and morphological description of fungal distribution and severity of infection in thirty-six mammal species
Carnivora	Lutra lutra	Weissenbacher-Lang et al. 2023	Detection of Pneumocystis and morphological description of fungal distribution and severity of infection in thirty-six mammal species
Carnivora	Martes foina	Settnes et al. 1986	Pneumocystis carinii Delanoe & Delanoe, 1912 found in lungs of freeliving animals in Denmark at Autopsy
Carnivora	Martes foina	Weissenbacher-Lang et al. 2023	Detection of Pneumocystis and morphological description of fungal distribution and severity of infection in thirty-six mammal species
Carnivora	Martes spp.	Weissenbacher-Lang et al. 2023	Detection of Pneumocystis and morphological description of fungal distribution and severity of infection in thirty-six mammal species
Carnivora	Meles meles	Settnes et al. 1986	Pneumocystis carinii Delanoe & Delanoe, 1912 found in lungs of freeliving animals in Denmark at Autopsy
Carnivora	Meles meles	Weissenbacher-Lang et al. 2023	Detection of Pneumocystis and morphological description of fungal distribution and severity of infection in thirty-six mammal species
Carnivora	Mephitis mephitis	Weissenbacher-Lang et al. 2023	Detection of Pneumocystis and morphological description of fungal distribution and severity of infection in thirty-six mammal species
Carnivora	Mustela lutreola	Weissenbacher-Lang et al. 2023	Detection of Pneumocystis and morphological description of fungal distribution and severity of infection in thirty-six mammal species
Carnivora	Mustela putorius furo	Dias et al. 2017	Extreme tetralogy of fallot with polycythemia in a ferret (Mustela putorius furo)
Carnivora	Mustela putorius furo	Weissenbacher-Lang et al. 2023	Detection of Pneumocystis and morphological description of fungal distribution and severity of infection in thirty-six mammal species
Carnivora	Neogale vison	Dyer and Schamber 1999	Pneumocystosis associated with canine distemper virus infection in a mink
Carnivora	Nasua narica	Lainson and Shaw 1975	Pneumocystis and Histoplasma infection in wild animals from the Amazon region of Brazil

Carnivora	Procyon lotor	Weissenbacher-Lang et al. 2023	Detection of Pneumocystis and morphological description of fungal distribution and severity of infection in thirty-six mammal species
Chiroptera	Eumops glaucinus	Cavallini Sanches et al. 2013	Pneumocystis spp. in bats evaluated by qPCR
Chiroptera	Eumops glaucinus	Veloso et al. 2014	Pneumocystis spp. and Histoplasma capsulatum in bats lungs in Southern and Midwestern Regions of Brazil
Chiroptera	Molossus currentium	Cavallini Sanches et al. 2013	Pneumocystis spp. in bats evaluated by qPCR
Chiroptera	Molossus currentium	Veloso et al. 2014	Pneumocystis spp. and Histoplasma capsulatum in bats lungs in Southern and Midwestern Regions of Brazil
Chiroptera	Molossus rufus	Cavallini Sanches et al. 2013	Pneumocystis spp. in bats evaluated by qPCR
Chiroptera	Molossus rufus	Veloso et al. 2014	Pneumocystis spp. and Histoplasma capsulatum in bats lungs in Southern and Midwestern Regions of Brazil
Chiroptera	Nyctinomops macrotis	Cavallini Sanches et al. 2013	Pneumocystis spp. in bats evaluated by qPCR
Chiroptera	Nyctinomops macrotis	Veloso et al. 2014	Pneumocystis spp. and Histoplasma capsulatum in bats lungs in Southern and Midwestern Regions of Brazil
Chiroptera	Promops nasutus	Cavallini Sanches et al. 2013	Pneumocystis spp. in bats evaluated by qPCR
Chiroptera	Promops nasutus	Veloso et al. 2014	Pneumocystis spp. and Histoplasma capsulatum in bats lungs in Southern and Midwestern Regions of Brazil
Chiroptera	Mormoops megalophylla	Akbar et al. 2012	Characterizing Pneumocystis in the lungs of bats: understanding Pneumocystis evolution and the spread of Pneumocystis organisms in mammal populations
Chiroptera	Mormoops megalophylla	González-González et al. 2014	Histoplasma capsulatum and Pneumocystis spp. co-infection in wild bats from Argentina, French Guyana, and Mexico
Chiroptera	Pteronotus davyi	Akbar et al. 2012	Characterizing Pneumocystis in the lungs of bats: understanding Pneumocystis evolution and the spread of Pneumocystis organisms in mammal populations
Chiroptera	Pteronotus davyi	González-González et al. 2014	Histoplasma capsulatum and Pneumocystis spp. co-infection in wild bats from Argentina, French Guyana, and Mexico
Chiroptera	Pteronotus parnellii	Akbar et al. 2012	Characterizing Pneumocystis in the lungs of bats: understanding Pneumocystis evolution and the spread of Pneumocystis organisms in mammal populations
Chiroptera	Pteronotus parnellii	González-González et al. 2014	Histoplasma capsulatum and Pneumocystis spp. co-infection in wild bats from Argentina, French Guyana, and Mexico

Chiroptera	Natalus stramineus	Akbar et al. 2012	Characterizing Pneumocystis in the lungs of bats: understanding Pneumocystis evolution and the spread of Pneumocystis organisms in mammal populations
Chiroptera	Natalus stramineus	González-González et al. 2014	Histoplasma capsulatum and Pneumocystis spp. co-infection in wild bats from Argentina, French Guyana, and Mexico
Chiroptera	Cynopterus spp.	Weissenbacher-Lang et al. 2023	Detection of Pneumocystis and morphological description of fungal distribution and severity of infection in thirty-six mammal species
Chiroptera	Pteropus giganteus	Weissenbacher-Lang et al. 2023	Detection of Pneumocystis and morphological description of fungal distribution and severity of infection in thirty-six mammal species
Chiroptera	Pteropus spp.	Weissenbacher-Lang et al. 2023	Detection of Pneumocystis and morphological description of fungal distribution and severity of infection in thirty-six mammal species
Chiroptera	Artibeus fimbriatus	Cavallini Sanches et al. 2013	Pneumocystis spp. in bats evaluated by qPCR
Chiroptera	Artibeus fimbriatus	Sanches et al. 2009	Detection of Pneumocystis in lungs of bats from Brazil by PCR amplification
Chiroptera	Artibeus fimbriatus	Veloso et al. 2014	Pneumocystis spp. and Histoplasma capsulatum in bats lungs in Southern and Midwestern Regions of Brazil
Chiroptera	Artibeus hirsutus	Akbar et al. 2012	Characterizing Pneumocystis in the lungs of bats: understanding Pneumocystis evolution and the spread of Pneumocystis organisms in mammal populations
Chiroptera	Artibeus hirsutus	González-González et al. 2014	Histoplasma capsulatum and Pneumocystis spp. co-infection in wild bats from Argentina, French Guyana, and Mexico
Chiroptera	Artibeus lituratus	Cavallini Sanches et al. 2013	Pneumocystis spp. in bats evaluated by qPCR
Chiroptera	Artibeus lituratus	Veloso et al. 2014	Pneumocystis spp. and Histoplasma capsulatum in bats lungs in Southern and Midwestern Regions of Brazil
Chiroptera	Artibeus spp.	Cavallini Sanches et al. 2013	Pneumocystis spp. in bats evaluated by qPCR
Chiroptera	Artibeus spp.	Veloso et al. 2014	Pneumocystis spp. and Histoplasma capsulatum in bats lungs in Southern and Midwestern Regions of Brazil
Chiroptera	Carollia perspicillata	Akbar et al. 2012	Characterizing Pneumocystis in the lungs of bats: understanding Pneumocystis evolution and the spread of Pneumocystis organisms in mammal populations
Chiroptera	Carollia perspicillata	Cavallini Sanches et al. 2013	Pneumocystis spp. in bats evaluated by qPCR

Chiroptera	Carollia perspicillata	González-González et al. 2014	Histoplasma capsulatum and Pneumocystis spp. co-infection in wild bats from Argentina, French Guyana, and Mexico
Chiroptera	Carollia perspicillata	Veloso et al. 2014	Pneumocystis spp. and Histoplasma capsulatum in bats lungs in Southern and Midwestern Regions of Brazil
Chiroptera	Diaemus youngii	Cavallini Sanches et al. 2013	Pneumocystis spp. in bats evaluated by qPCR
Chiroptera	Diaemus youngii	Veloso et al. 2014	Pneumocystis spp. and Histoplasma capsulatum in bats lungs in Southern and Midwestern Regions of Brazil
Chiroptera	Diphylla ecaudata	Sanches et al. 2009	Detection of Pneumocystis in lungs of bats from Brazil by PCR amplification
Chiroptera	Diphylla ecaudata	Veloso et al. 2014	Pneumocystis spp. and Histoplasma capsulatum in bats lungs in Southern and Midwestern Regions of Brazil
Chiroptera	Glossophaga soricina	Cavallini Sanches et al. 2013	Pneumocystis spp. in bats evaluated by qPCR
Chiroptera	Glossophaga soricina	Veloso et al. 2014	Pneumocystis spp. and Histoplasma capsulatum in bats lungs in Southern and Midwestern Regions of Brazil
Chiroptera	Rhinophylla pumilio	Veloso et al. 2014	Pneumocystis spp. and Histoplasma capsulatum in bats lungs in Southern and Midwestern Regions of Brazil
Chiroptera	Sturnira lilium	Cavallini Sanches et al. 2013	Pneumocystis spp. in bats evaluated by qPCR
Chiroptera	Sturnira lilium	Sanches et al. 2009	Detection of Pneumocystis in lungs of bats from Brazil by PCR amplification
Chiroptera	Sturnira lilium	Veloso et al. 2014	Pneumocystis spp. and Histoplasma capsulatum in bats lungs in Southern and Midwestern Regions of Brazil
Chiroptera	Rhinolophus hipposideros	Weissenbacher-Lang et al. 2023	Detection of Pneumocystis and morphological description of fungal distribution and severity of infection in thirty-six mammal species
Chiroptera	Aeorestes cinereus	Veloso et al. 2014	Pneumocystis spp. and Histoplasma capsulatum in bats lungs in Southern and Midwestern Regions of Brazil
Chiroptera	Eptesicus furinalis	Cavallini Sanches et al. 2013	Pneumocystis spp. in bats evaluated by qPCR
Chiroptera	Eptesicus furinalis	Veloso et al. 2014	Pneumocystis spp. and Histoplasma capsulatum in bats lungs in Southern and Midwestern Regions of Brazil
Chiroptera	Eptesicus serotinus	Akbar et al. 2012	Characterizing Pneumocystis in the lungs of bats: understanding Pneumocystis evolution and the spread of Pneumocystis organisms in mammal populations

Chiroptera	Eptesicus serotinus	Weissenbacher-Lang et al. 2023	Detection of Pneumocystis and morphological description of fungal distribution and severity of infection in thirty-six mammal species
Chiroptera	Hypsugo savii	Weissenbacher-Lang et al. 2023	Detection of Pneumocystis and morphological description of fungal distribution and severity of infection in thirty-six mammal species
Chiroptera	Lasiurus blossevillii	Cavallini Sanches et al. 2013	Pneumocystis spp. in bats evaluated by qPCR
Chiroptera	Lasiurus blossevillii	Veloso et al. 2014	Pneumocystis spp. and Histoplasma capsulatum in bats lungs in Southern and Midwestern Regions of Brazil
Chiroptera	Myotis californicus	Akbar et al. 2012	Characterizing Pneumocystis in the lungs of bats: understanding Pneumocystis evolution and the spread of Pneumocystis organisms in mammal populations
Chiroptera	Myotis californicus	González-González et al. 2014	Histoplasma capsulatum and Pneumocystis spp. co-infection in wild bats from Argentina, French Guyana, and Mexico
Chiroptera	Myotis daubentoni	Akbar et al. 2012	Characterizing Pneumocystis in the lungs of bats: understanding Pneumocystis evolution and the spread of Pneumocystis organisms in mammal populations
Chiroptera	Myotis levis	Cavallini Sanches et al. 2013	Pneumocystis spp. in bats evaluated by qPCR
Chiroptera	Myotis levis	Sanches et al. 2009	Detection of Pneumocystis in lungs of bats from Brazil by PCR amplification
Chiroptera	Myotis levis	Veloso et al. 2014	Pneumocystis spp. and Histoplasma capsulatum in bats lungs in Southern and Midwestern Regions of Brazil
Chiroptera	Myotis myotis	Akbar et al. 2012	Characterizing Pneumocystis in the lungs of bats: understanding Pneumocystis evolution and the spread of Pneumocystis organisms in mammal populations
Chiroptera	Myotis mystacinus	Weissenbacher-Lang et al. 2023	Detection of Pneumocystis and morphological description of fungal distribution and severity of infection in thirty-six mammal species
Chiroptera	Myotis nigricans	Cavallini Sanches et al. 2013	Pneumocystis spp. in bats evaluated by qPCR
Chiroptera	Myotis nigricans	Veloso et al. 2014	Pneumocystis spp. and Histoplasma capsulatum in bats lungs in Southern and Midwestern Regions of Brazil
Chiroptera	Nyctalus leisleri	Akbar et al. 2012	Characterizing Pneumocystis in the lungs of bats: understanding Pneumocystis evolution and the spread of Pneumocystis organisms in mammal populations

Chiroptera	Nyctalus noctula	Weissenbacher-Lang et al. 2023	Detection of Pneumocystis and morphological description of fungal distribution and severity of infection in thirty-six mammal species
Chiroptera	Pipistrellus pipistrellus	Akbar et al. 2012	Characterizing Pneumocystis in the lungs of bats: understanding Pneumocystis evolution and the spread of Pneumocystis organisms in mammal populations
Chiroptera	Pipistrellus pipistrellus	Derouiche et al. 2009	Pneumocystis diversity as a phyleographic tool
Chiroptera	Plecotus auritus	Akbar et al. 2012	Characterizing Pneumocystis in the lungs of bats: understanding Pneumocystis evolution and the spread of Pneumocystis organisms in mammal populations
Chiroptera	Plecotus austriacus	Akbar et al. 2012	Characterizing Pneumocystis in the lungs of bats: understanding Pneumocystis evolution and the spread of Pneumocystis organisms in mammal populations
Chiroptera	Vespertilio murinus	Weissenbacher-Lang et al. 2023	Detection of Pneumocystis and morphological description of fungal distribution and severity of infection in thirty-six mammal species
Chiroptera	Vespertilio spp.	Weissenbacher-Lang et al. 2023	Detection of Pneumocystis and morphological description of fungal distribution and severity of infection in thirty-six mammal species
Diprotodontia	Osphranter rufus	Poelma 1975	Pneumocystis carinii infections in zoo animals
Eulipotyphla	Atelerix albiventris	Weissenbacher-Lang et al. 2023	Detection of Pneumocystis and morphological description of fungal distribution and severity of infection in thirty-six mammal species
Eulipotyphla	Erinaceus europaeus	Settnes et al. 1986	Pneumocystis carinii Delanoe & Delanoe, 1912 found in lungs of freeliving animals in Denmark at Autopsy
Eulipotyphla	Erinaceus roumanicus	Weissenbacher-Lang et al. 2023	Detection of Pneumocystis and morphological description of fungal distribution and severity of infection in thirty-six mammal species
Eulipotyphla	Crocidura leucodon	Weissenbacher-Lang et al. 2023	Detection of Pneumocystis and morphological description of fungal distribution and severity of infection in thirty-six mammal species
Eulipotyphla	Crocidura suaveolens	Sebek and Rosicky 1967	The finding of Pneumocystis carinii in shrews (Incestivora:Soricidae)
Eulipotyphla	Crocidura suaveolens	Weissenbacher-Lang et al. 2023	Detection of Pneumocystis and morphological description of fungal distribution and severity of infection in thirty-six mammal species
Eulipotyphla	Neomys fodiens	Kučera et al. 1971	Pneumocystosis
Eulipotyphla	Sorex araneus	Mazars et al. 1997	Detection of Pneumocystis in European wild animals
Eulipotyphla	Sorex araneus	Kučera et al. 1971	Pneumocystosis

Eulipotyphla	Sorex araneus	Settnes and Lodal 1980	Prevalence of Pneumocystis carinii Delanoë & Delanoë, 1912 in rodents in Denmark
Eulipotyphla	Sorex isodon	Laakkonen 1998	Pneumocystis carinii in wildlife
Eulipotyphla	Sorex minutissimus	Laakkonen 1998	Pneumocystis carinii in wildlife
Eulipotyphla	Sorex minutus	Weissenbacher-Lang et al. 2023	Detection of Pneumocystis and morphological description of fungal distribution and severity of infection in thirty-six mammal species
Eulipotyphla	Sorex minutus	Kučera et al. 1971	Pneumocystosis
Eulipotyphla	Sorex minutus	Laakkonen 1998	Pneumocystis carinii in wildlife
Eulipotyphla	Sorex minutus	Kučera et al. 1971	Pneumocystosis
Eulipotyphla	Sorex minutus	Sebek and Rosicky 1967	The finding of Pneumocystis carinii in shrews (Incestivora:Soricidae)
Eulipotyphla	Sorex spp.	Peters et al. 1994	DNA analysis of Pneumocystis carinii infecting Finnish and English shrews
Eulipotyphla	Sorex spp.	Peters et al. 1994	DNA analysis of Pneumocystis carinii infecting Finnish and English shrews
Eulipotyphla	Talpa europaea	Mazars et al. 1997	Detection of Pneumocystis in European wild animals
Eulipotyphla	Talpa europaea	Weissenbacher-Lang et al. 2023	Detection of Pneumocystis and morphological description of fungal distribution and severity of infection in thirty-six mammal species
Eulipotyphla	Tupaia glis	Poelma 1975	Pneumocystis carinii infections in zoo animals
Eulipotyphla	Tupaia glis	Weissenbacher-Lang et al. 2023	Detection of Pneumocystis and morphological description of fungal distribution and severity of infection in thirty-six mammal species
Hyracoidea	Dendrohyrax arboreus	Poelma 1975	Pneumocystis carinii infections in zoo animals
Hyracoidea	Procavia capensis	Poelma 1975	Pneumocystis carinii infections in zoo animals
Lagomorpha	Lepus europaeus	Weissenbacher-Lang et al. 2023	Detection of Pneumocystis and morphological description of fungal distribution and severity of infection in thirty-six mammal species
Lagomorpha	Oryctolagus cuniculus	Poelma 1975	Pneumocystis carinii infections in zoo animals
Perissodactyla	Equus asinus	Weissenbacher-Lang et al. 2023	Detection of Pneumocystis and morphological description of fungal distribution and severity of infection in thirty-six mammal species
Perissodactyla	Equus caballus	Clark-Price et al. 2004	Use of dapsone in the treatment of Pneumocystis carinii pneumonia in a foal
Perissodactyla	Equus caballus	Franklin et al. 2002	Proliferative interstitial pneumonia, Pneumocystis carinii infection, and immunodeficiency in an adult Paso Fino horse

Perissodactyla	Equus caballus	Perron Lepage et al. 1999	A case of interstitial pneumonia associated with Pneumocystis carinii in a foal
Perissodactyla	Equus caballus	Tanaka et al. 1994	Pneumocystis carinii pneumonia in a thoroughbred foal
Perissodactyla	Equus caballus	Shively et al. 1974	Fine structure of spontaneous Pneumocystis carinii pulmonary infection in foals
Perissodactyla	Equus caballus	Shively et al. 1973	Pneumocystis carinii pneumonia in two foals
Perissodactyla	Equus caballus	Ewing et al. 1994	Pneumocystis carinii pneumonia in foals
Perissodactyla	Equus caballus	Jensen et al. 2001	Application of fluorescent in situ hybridization for specific diagnosis of Pneumocystis carinii pneumonia in foals and pigs
Perissodactyla	Equus caballus	Punsmann et al. 2022	Acute interstitial pneumonia in foals: a severe, multifactorial syndrome with lung tissue recovery in surviving foals
Perissodactyla	Equus caballus	Peters et al. 1994	Pneumocystis carinii pneumonia in thoroughbred foals: identification of a genetically distinct organism by DNA amplification
Perissodactyla	Equus quagga	Weissenbacher-Lang et al. 2023	Detection of Pneumocystis and morphological description of fungal distribution and severity of infection in thirty-six mammal species
Pilosa	Bradypus tridactylus	Poelma 1975	Pneumocystis carinii infections in zoo animals
Pilosa	Bradypus tridactylus	Lainson and Shaw 1975	Pneumocystis and Histoplasma infections in wild animals from the Amazon region of Brazil
Pilosa	Bradypus variegatus	Yonushonis et al. 1986	Infection of a three-toed sloth (Bradypus variegatus) by a Pneumocystis-like organism in Panama
Pilosa	Choloepus didactylus	Lainson and Shaw 1975	Pneumocystis and Histoplasma infections in wild animals from the Amazon region of Brazil
Primates	Aotus trivirgatus	Weissenbacher-Lang et al. 2023	Detection of Pneumocystis and morphological description of fungal distribution and severity of infection in thirty-six mammal species
Primates	Alouatta fusca	Poelma 1975	Pneumocystis carinii infections in zoo animals
Primates	Ateles belzebuth	Poelma 1975	Pneumocystis carinii infections in zoo animals
Primates	Lagothrix lagothricha	Poelma 1975	Pneumocystis carinii infections in zoo animals
Primates	Callimico goeldii	Demanche et al. 2001	Phylogeny of Pneumocystis carinii from 18 primate species confirms host specificity and suggests coevolution
Primates	Callithrix aurita	Poelma 1975	Pneumocystis carinii infections in zoo animals

Primates	Callithrix geoffroyi	Demanche et al. 2001	Phylogeny of Pneumocystis carinii from 18 primate species confirms host specificity and suggests coevolution
Primates	Leontocebus fuscicollis	Weissenbacher-Lang et al. 2023	Detection of Pneumocystis and morphological description of fungal distribution and severity of infection in thirty-six mammal species
Primates	Leontopithecus rosalia	Weissenbacher-Lang et al. 2023	Detection of Pneumocystis and morphological description of fungal distribution and severity of infection in thirty-six mammal species
Primates	Saguinus fuscicollis	Demanche et al. 2001	Phylogeny of Pneumocystis carinii from 18 primate species confirms host specificity and suggests coevolution
Primates	Saguinus imperator	Demanche et al. 2001	Phylogeny of Pneumocystis carinii from 18 primate species confirms host specificity and suggests coevolution
Primates	Saguinus midas	Demanche et al. 2001	Phylogeny of Pneumocystis carinii from 18 primate species confirms host specificity and suggests coevolution
Primates	Saguinus oedipus	Weissenbacher-Lang et al. 2023	Detection of Pneumocystis and morphological description of fungal distribution and severity of infection in thirty-six mammal species
Primates	Saguinus oedipus	Demanche et al. 2001	Phylogeny of Pneumocystis carinii from 18 primate species confirms host specificity and suggests coevolution
Primates	Cebus capucinus	Weissenbacher-Lang et al. 2023	Detection of Pneumocystis and morphological description of fungal distribution and severity of infection in thirty-six mammal species
Primates	Saimiri sciureus	Weissenbacher-Lang et al. 2023	Detection of Pneumocystis and morphological description of fungal distribution and severity of infection in thirty-six mammal species
Primates	Saimiri sciureus	Demanche et al. 2001	Phylogeny of Pneumocystis carinii from 18 primate species confirms host specificity and suggests coevolution
Primates	Allenopithecus nigroviridis	Demanche et al. 2001	Phylogeny of Pneumocystis carinii from 18 primate species confirms host specificity and suggests coevolution
Primates	Cercopithecus hamlyni	Demanche et al. 2001	Phylogeny of Pneumocystis carinii from 18 primate species confirms host specificity and suggests coevolution
Primates	Cercopithecus nictitans	Demanche et al. 2001	Phylogeny of Pneumocystis carinii from 18 primate species confirms host specificity and suggests coevolution
Primates	Cercopithecus/Miopithecus spp.	Weissenbacher-Lang et al. 2023	Detection of Pneumocystis and morphological description of fungal distribution and severity of infection in thirty-six mammal species
Primates	Colobus guereza	Weissenbacher-Lang et al. 2023	Detection of Pneumocystis and morphological description of fungal distribution and severity of infection in thirty-six mammal species

Primates	Colobus polykomos	Weissenbacher-Lang et al. 2023	Detection of Pneumocystis and morphological description of fungal distribution and severity of infection in thirty-six mammal species
Primates	Macaca fascicularis	Demanche et al. 2001	Phylogeny of Pneumocystis carinii from 18 primate species confirms host specificity and suggests coevolution
Primates	Macaca mulatta	Weissenbacher-Lang et al. 2023	Detection of Pneumocystis and morphological description of fungal distribution and severity of infection in thirty-six mammal species
Primates	Macaca mulatta	Demanche et al. 2001	Phylogeny of Pneumocystis carinii from 18 primate species confirms host specificity and suggests coevolution
Primates	Macaca nemestrina	Demanche et al. 2001	Phylogeny of Pneumocystis carinii from 18 primate species confirms host specificity and suggests coevolution
Primates	Macaca sylvanus	Weissenbacher-Lang et al. 2023	Detection of Pneumocystis and morphological description of fungal distribution and severity of infection in thirty-six mammal species
Primates	Theropithecus gelada	Weissenbacher-Lang et al. 2023	Detection of Pneumocystis and morphological description of fungal distribution and severity of infection in thirty-six mammal species
Primates	Galago demidoff	Poelma 1975	Pneumocystis carinii infections in zoo animals
Primates	Galago senegalensis	Poelma 1975	Pneumocystis carinii infections in zoo animals
Primates	Pan troglodytes	Poelma 1975	Pneumocystis carinii infections in zoo animals
Primates	Pongo spp.	Weissenbacher-Lang et al. 2023	Detection of Pneumocystis and morphological description of fungal distribution and severity of infection in thirty-six mammal species
Primates	Eulemur macaco	Demanche et al. 2001	Phylogeny of Pneumocystis carinii from 18 primate species confirms host specificity and suggests coevolution
Primates	Hapalemur griseus	Demanche et al. 2001	Phylogeny of Pneumocystis carinii from 18 primate species confirms host specificity and suggests coevolution
Primates	Pithecia pithecia	Weissenbacher-Lang et al. 2023	Detection of Pneumocystis and morphological description of fungal distribution and severity of infection in thirty-six mammal species
Primates	Pithecia pithecia	Demanche et al. 2001	Phylogeny of Pneumocystis carinii from 18 primate species confirms host specificity and suggests coevolution
Rodentia	Heliophobius argenteocinereus	Petružela et al. 2019	Evolutionary history of Pneumocystis fungi in their African rodent hosts
Rodentia	Castor fiber	Weissenbacher-Lang et al. 2023	Detection of Pneumocystis and morphological description of fungal distribution and severity of infection in thirty-six mammal species
Rodentia	Cavia porcellus	Shimizu et al. 1985	Occurrence of Pneumocystis carinii in animals in Japan

Rodentia	Cavia porcellus	Weissenbacher-Lang et al. 2023	Detection of Pneumocystis and morphological description of fungal distribution and severity of infection in thirty-six mammal species
Rodentia	Kerodon rupestris	Weissenbacher-Lang et al. 2023	Detection of Pneumocystis and morphological description of fungal distribution and severity of infection in thirty-six mammal species
Rodentia	Chinchilla lanigera	Weissenbacher-Lang et al. 2023	Detection of Pneumocystis and morphological description of fungal distribution and severity of infection in thirty-six mammal species
Rodentia	Arvicola terrestris	Settnes and Lodal 1980	Prevalence of Pneumocystis carinii Delanoë & Delanoë, 1912 in rodents in Denmark
Rodentia	Cricetus cricetus	Weissenbacher-Lang et al. 2023	Detection of Pneumocystis and morphological description of fungal distribution and severity of infection in thirty-six mammal species
Rodentia	Hylaeamys megacephalus	Lainson and Shaw 1975	Pneumocystis and Histoplasma infections in wild animals from the Amazon region of Brazil#
Rodentia	Mesocricetus auratus	Weissenbacher-Lang et al. 2023	Detection of Pneumocystis and morphological description of fungal distribution and severity of infection in thirty-six mammal species
Rodentia	Microtus agrestis	Kučera et al. 1971	Pneumocystosis
Rodentia	Microtus agrestis	Settnes and Lodal 1980	Prevalence of Pneumocystis carinii Delanoë & Delanoë, 1912 in rodents in Denmark
Rodentia	Microtus agrestis	Mazars et al. 1997	Detection of Pneumocystis in European wild animals
Rodentia	Microtus arvalis	Sebek and Rosicky 1967	The finding of Pneumocystis carinii in shrews (Incestivora:Soricidae)
Rodentia	Microtus multiplex	Danesi et al. 2016	Barcoding markers for Pneumocystis species in wildlife
Rodentia	Microtus subterraneus	Kučera et al. 1971	Pneumocystosis
Rodentia	Myodes glareolus	Kučera et al. 1971	Pneumocystosis
Rodentia	Myodes glareolus	Danesi et al. 2016	Barcoding markers for Pneumocystis species in wildlife
Rodentia	Myodes glareolus	Settnes and Lodal 1980	Prevalence of Pneumocystis carinii Delanoë & Delanoë, 1912 in rodents in Denmark
Rodentia	Myodes rufocanus	Shiota et al. 1986	Prevalence of Pneumocystis carinii in wild rodents in Japan
Rodentia	Peromyscus boylii	Laakkonen et al. 2001	Pneumocystis in wild small mammals from California
Rodentia	Peromyscus maniculatus	Laakkonen et al. 2001	Pneumocystis in wild small mammals from California
Rodentia	Phodopus sungorus	Weissenbacher-Lang et al. 2023	Detection of Pneumocystis and morphological description of fungal distribution and severity of infection in thirty-six mammal species
Rodentia	Eliomys quercinus	Mazars et al. 1997	Detection of Pneumocystis in European wild animals

Rodentia	Graphiurus spp.	Petružela et al. 2019	Evolutionary history of Pneumocystis fungi in their African rodent hosts
Rodentia	Muscardinus avellanarius	Sebek and Rosicky 1967	The finding of Pneumocystis carinii in shrews (Incestivora:Soricidae)
Rodentia	Chaetodipus californicus	Laakkonen et al. 2001	Pneumocystis in wild small mammals from California
Rodentia	Acomys ignitus	Petružela et al. 2019	Evolutionary history of Pneumocystis fungi in their African rodent hosts
Rodentia	Acomys muzei	Petružela et al. 2019	Evolutionary history of Pneumocystis fungi in their African rodent hosts
Rodentia	Acomys ngurui	Petružela et al. 2019	Evolutionary history of Pneumocystis fungi in their African rodent hosts
Rodentia	Aethomys hindei	Petružela et al. 2019	Evolutionary history of Pneumocystis fungi in their African rodent hosts
Rodentia	Aethomys kaiseri	Petružela et al. 2019	Evolutionary history of Pneumocystis fungi in their African rodent hosts
Rodentia	Apodemus flavicollis	Settnes and Lodal 1980	Prevalence of Pneumocystis carinii Delanoë & Delanoë, 1912 in rodents in Denmark
Rodentia	Apodemus sylvaticus	Settnes and Lodal 1980	Prevalence of Pneumocystis carinii Delanoë & Delanoë, 1912 in rodents in Denmark
Rodentia	Apomys banahao	Latinne et al. 2021	Revisiting the Pneumocystis host specificity paradigm and transmission ecology in wild Southeast Asian rodents
Rodentia	Berylmys bowersi	Latinne et al. 2021	Revisiting the Pneumocystis host specificity paradigm and transmission ecology in wild Southeast Asian rodents
Rodentia	Berylmys bowersi	Latinne et al. 2018	Genetic diversity and evolution of Pneumocystis fungi infecting wild Southeast Asian murid rodents
Rodentia	Grammomys surdaster	Petružela et al. 2019	Evolutionary history of Pneumocystis fungi in their African rodent hosts
Rodentia	Hylomyscus arcimontensis	Petružela et al. 2019	Evolutionary history of Pneumocystis fungi in their African rodent hosts
Rodentia	Lemniscomys rosalia	Petružela et al. 2019	Evolutionary history of Pneumocystis fungi in their African rodent hosts
Rodentia	Lemniscomys striatus	Petružela et al. 2019	Evolutionary history of Pneumocystis fungi in their African rodent hosts

Rodentia	Leopoldamys herberti	Latinne et al. 2018	Genetic diversity and evolution of Pneumocystis fungi infecting wild Southeast Asian murid rodents
Rodentia	Leopoldamys neilli	Latinne et al. 2021	Revisiting the Pneumocystis host specificity paradigm and transmission ecology in wild Southeast Asian rodents
Rodentia	Leopoldamys sabanus	Latinne et al. 2021	Revisiting the Pneumocystis host specificity paradigm and transmission ecology in wild Southeast Asian rodents
Rodentia	Lophuromys kilonzoi	Petružela et al. 2019	Evolutionary history of Pneumocystis fungi in their African rodent hosts
Rodentia	Lophuromys makundii	Petružela et al. 2019	Evolutionary history of Pneumocystis fungi in their African rodent hosts
Rodentia	Meriones unguiculatus	Weissenbacher-Lang et al. 2023	Detection of Pneumocystis and morphological description of fungal distribution and severity of infection in thirty-six mammal species
Rodentia	Micromys minutus	Shiota et al. 1986	Prevalence of Pneumocystis carinii in wild rodents in Japan
Rodentia	Micromys minutus	Shiota et al. 1986	Prevalence of Pneumocystis carinii in wild rodents in Japan
Rodentia	Mus musculus	Shimizu et al. 1985	Occurrence of Pneumocystis carinii in animals in Japan
Rodentia	Mus musculus	Sebek and Rosicky 1967	The finding of Pneumocystis carinii in shrews (Incestivora:Soricidae)
Rodentia	Mus musculus	Shiota et al. 1986	Prevalence of Pneumocystis carinii in wild rodents in Japan
Rodentia	Mus musculus	Weissenbacher-Lang et al. 2023	Detection of Pneumocystis and morphological description of fungal distribution and severity of infection in thirty-six mammal species
Rodentia	Mus pahari	Latinne et al. 2021	Revisiting the Pneumocystis host specificity paradigm and transmission ecology in wild Southeast Asian rodents
Rodentia	Mus saxicola	Weissenbacher-Lang et al. 2023	Detection of Pneumocystis and morphological description of fungal distribution and severity of infection in thirty-six mammal species
Rodentia	Myomyscus brockmani	Petružela et al. 2019	Evolutionary history of Pneumocystis fungi in their African rodent hosts
Rodentia	Niviventer fulvescens	Latinne et al. 2018	Genetic diversity and evolution of Pneumocystis fungi infecting wild Southeast Asian murid rodents
Rodentia	Otomys angoniensis	Petružela et al. 2019	Evolutionary history of Pneumocystis fungi in their African rodent hosts
Rodentia	Praomys delectorum	Petružela et al. 2019	Evolutionary history of Pneumocystis fungi in their African rodent hosts

Rodentia	Rattus andamanensis	Latinne et al. 2021	Revisiting the Pneumocystis host specificity paradigm and transmission ecology in wild Southeast Asian rodents
Rodentia	Rattus andamanensis	Latinne et al. 2018	Genetic diversity and evolution of Pneumocystis fungi infecting wild Southeast Asian murid rodents
Rodentia	Rattus everetti	Latinne et al. 2021	Revisiting the Pneumocystis host specificity paradigm and transmission ecology in wild Southeast Asian rodents
Rodentia	Rattus norvegicus	Shimizu et al. 1985	Occurrence of Pneumocystis carinii in animals in Japan
Rodentia	Rattus norvegicus	Kučera et al. 1971	Pneumocystosis
Rodentia	Rattus norvegicus	Yoshida and Ikai 1979	Pneumocystis carinii pneumonia: epidemiology in Japan and cyst concentration method
Rodentia	Rattus rattus	Settnes and Lodal 1980	Prevalence of Pneumocystis carinii Delanoë & Delanoë, 1912 in rodents in Denmark
Rodentia	Rattus rattus	Weissenbacher-Lang et al. 2023	Detection of Pneumocystis and morphological description of fungal distribution and severity of infection in thirty-six mammal species
Rodentia	Rattus spp.	Shimizu et al. 1985	Occurrence of Pneumocystis carinii in animals in Japan
Rodentia	Rhabdomys dilectus	Petružela et al. 2019	Evolutionary history of Pneumocystis fungi in their African rodent hosts
Rodentia	Sundamys muelleri	Latinne et al. 2021	Revisiting the Pneumocystis host specificity paradigm and transmission ecology in wild Southeast Asian rodents
Rodentia	Myocastor coypus	Weissenbacher-Lang et al. 2023	Detection of Pneumocystis and morphological description of fungal distribution and severity of infection in thirty-six mammal species
Rodentia	Saccostomus umbriventer	Petružela et al. 2019	Evolutionary history of Pneumocystis fungi in their African rodent hosts
Rodentia	Steatomys ssp.	Petružela et al. 2019	Evolutionary history of Pneumocystis fungi in their African rodent hosts
Rodentia	Octodon degus	Weissenbacher-Lang et al. 2023	Detection of Pneumocystis and morphological description of fungal distribution and severity of infection in thirty-six mammal species
Rodentia	Funambulus palmarum	Poelma 1975	Pneumocystis carinii infections in zoo animals
Rodentia	Sciurus vulgaris	Weissenbacher-Lang et al. 2023	Detection of Pneumocystis and morphological description of fungal distribution and severity of infection in thirty-six mammal species
Rodentia	Tamias spp.	Weissenbacher-Lang et al. 2023	Detection of Pneumocystis and morphological description of fungal distribution and severity of infection in thirty-six mammal species

Rodentia	Tamiops swinhoei	Weissenbacher-Lang et al. 2023	Detection of Pneumocystis and morphological description of fungal distribution and severity of infection in thirty-six mammal species
Rodentia	Cannomys badius	Latinne et al. 2021	Revisiting the Pneumocystis host specificity paradigm and transmission ecology in wild Southeast Asian rodents
Rodentia	Cannomys badius	Latinne et al. 2018	Genetic diversity and evolution of Pneumocystis fungi infecting wild Southeast Asian murid rodents
Rodentia	Rhizomys pruinosus	Latinne et al. 2021	Revisiting the Pneumocystis host specificity paradigm and transmission ecology in wild Southeast Asian rodents
Rodentia	Tachyoryctes splendens	Petružela et al. 2019	Evolutionary history of Pneumocystis fungi in their African rodent hosts

No exact samples size indicated (n = 7)

Afrosoricida	Microgale spp.	Laakkonen 1998	Pneumocystis carinii in wildlife
Carnivora	Galictis vittata	Guillot et al. 2001	Parallel phylogenies of Pneumocystis species and their mammalian hosts
Chiroptera	Glossophaga soricina	Guillot et al. 2001	Parallel phylogenies of Pneumocystis species and their mammalian hosts
Didelphimorphia	Marmosa murina	Guillot et al. 2001	Parallel phylogenies of Pneumocystis species and their mammalian hosts
Rodentia	Agouti paca	Guillot et al. 2001	Parallel phylogenies of Pneumocystis species and their mammalian hosts
Rodentia	Proechimys guyannensis	Guillot et al. 2001	Parallel phylogenies of Pneumocystis species and their mammalian hosts
Rodentia	Sciurus aestuans	Guillot et al. 2001	Parallel phylogenies of Pneumocystis species and their mammalian hosts

No number of positive samples indicated (n = 1)

Artiodactyla	Sus scrofa domesticus	Sanches et al. 2011	Phylogenetic analysis of Pneumocystis from pig lungs obtained from slaughterhouses in southern and midwestern regions of Brazil

Selected pre-defined samples (n = 1)

Artiodactyla	Sus scrofa domesticus	Weissenbacher-Lang et al. 2016	Establishment of a quantitative real-time PCR for the detection of Pneumocystis carinii f. sp. suis in bronchoalveolar lavage samples from pigs

Studies on exclusively positive samples (n = 1)

Artiodactyla	Sus scrofa domesticus	Weissenbacher-Lang et al. 2016	Retrospective Analysis of Bacterial and Viral Co-Infections in Pneumocystis spp. Positive Lung Samples of Austrian Pigs with Pneumonia

Same samples used as in previous study (n = 1)

Artiodactyla	Sus scrofa domesticus	Sanches et al. 2007	Detection of Pneumocystis spp. in lung samples from pigs in Brazil

Species not exactly defined (n = 5)

Rodentia	Peromyscus spp.	Salazar-Hamm et al. 2022	Breathing can be dangerous: Opportunistic fungal pathogens and the diverse community of the small mammal lung microbiome
Rodentia	Dipodomys spp.	Salazar-Hamm et al. 2022	Breathing can be dangerous: Opportunistic fungal pathogens and the diverse community of the small mammal lung microbiome
Rodentia	Apodemus spp.	Kučera et al. 1971	Pneumocystosis
Rodentia	Rattus rattus/Rattus norvegicus	Rothenburger et al. 2015	Respiratory Pathology and Pathogens in Wild Urban Rats (Rattus norvegicus and Rattus rattus)
Rodentia	Rattus spp.	Meer and Brug 1942	Infection a Pneumocystis chez l'homme et chez les animaux

Serological studies (n = 11)

Artiodactyla	Bos taurus	Kučera et al. 1971	Pneumocystosis
Artiodactyla	Sus scrofa domesticus	Kučera et al. 1971	Pneumocystosis

Artiodactyla	Sus scrofa domesticus	Weissenbacher-Lang et al. 2017	Comparison of Pneumocystis nucleic acid and antibody profiles and their associations with other respiratory pathogens in two Austrian pig herds
Primates	Macaca fascicularis	Demanche et al. 2005	Molecular and serological evidence of Pneumocystis circulation in a social organization of healthy macaques (Macaca fascicularis)
Rodentia	Microtus agrestis	Kučera et al. 1971	Pneumocystosis
Rodentia	Microtus arvalis	Kučera et al. 1971	Pneumocystosis
Rodentia	Microtus subterraneus	Kučera et al. 1971	Pneumocystosis
Rodentia	Myodes glareolus	Kučera et al. 1971	Pneumocystosis
Rodentia	Apodemus flavicollis	Kučera et al. 1971	Pneumocystosis
Rodentia	Apodemus sylvaticus	Kučera et al. 1971	Pneumocystosis
Rodentia	Rattus rattus/Rattus norvegicus	Boey et al. 2019	Seroprevalence of Rodent Pathogens in Wild Rats from the Island of St. Kitts, West Indies

Method not defined (n = 1)

Carnivora	Canis lupus familiaris	Brownlie 1990	A retrospective study of diagnosis in 109 cases of canine lower respiratory disease

2. Prevalence in wild animals.

Sample sizes ≤ 10 samples (n = 180)

Artiodactyla	Hippotragus niger	Wilson et al. 1974	Observations on mortality rates and disease in roan and sable antelope on nature reserves in the Transvaal
Artiodactyla	Rupicapra rupicapra	Weissenbacher-Lang et al. 2023	Detection of Pneumocystis and morphological description of fungal distribution and severity of infection in thirty-six mammal species
Artiodactyla	Capreolus capreolus	Settnes et al. 1986	Pneumocystis carinii Delanoe & Delanoe, 1912 found in lungs of freeliving animals in Denmark at Autopsy

Artiodactyla	Capreolus capreolus	Weissenbacher-Lang et al. 2023	Detection of Pneumocystis and morphological description of fungal distribution and severity of infection in thirty-six mammal species
Artiodactyla	Cervus elaphus	Weissenbacher-Lang et al. 2023	Detection of Pneumocystis and morphological description of fungal distribution and severity of infection in thirty-six mammal species
Artiodactyla	Cervus spp.	Yoshida and Ikai 1979	Pneumocystis carinii pneumonia: epidemiology in Japan and cyst concentration method
Artiodactyla	Elaphurus davidianus	Weissenbacher-Lang et al. 2023	Detection of Pneumocystis and morphological description of fungal distribution and severity of infection in thirty-six mammal species
Artiodactyla	Rangifer tarandus	Weissenbacher-Lang et al. 2023	Detection of Pneumocystis and morphological description of fungal distribution and severity of infection in thirty-six mammal species
Artiodactyla	Rangifer tarandus groenlandicus	Settnes et al. 1986	Pneumocystis carinii Delanoe & Delanoe, 1912 found in lungs of freeliving animals in Denmark at Autopsy
Artiodactyla	Sus scrofa	Yoshida and Ikai 1979	Pneumocystis carinii pneumonia: epidemiology in Japan and cyst concentration method
Carnivora	Vulpes vulpes	Weissenbacher-Lang et al. 2023	Detection of Pneumocystis and morphological description of fungal distribution and severity of infection in thirty-six mammal species
Carnivora	Vulpes vulpes	Yoshida and Ikai 1979	Pneumocystis carinii pneumonia: epidemiology in Japan and cyst concentration method
Carnivora	Vulpes vulpes	Meer and Brug 1942	Infection a Pneumocystis chez l'homme et chez les animaux
Carnivora	Martes foina	Settnes et al. 1986	Pneumocystis carinii Delanoe & Delanoe, 1912 found in lungs of freeliving animals in Denmark at Autopsy
Carnivora	Martes foina	Weissenbacher-Lang et al. 2023	Detection of Pneumocystis and morphological description of fungal distribution and severity of infection in thirty-six mammal species
Carnivora	Martes spp.	Weissenbacher-Lang et al. 2023	Detection of Pneumocystis and morphological description of fungal distribution and severity of infection in thirty-six mammal species
Carnivora	Meles meles	Settnes et al. 1986	Pneumocystis carinii Delanoe & Delanoe, 1912 found in lungs of freeliving animals in Denmark at Autopsy
Carnivora	Meles meles	Weissenbacher-Lang et al. 2023	Detection of Pneumocystis and morphological description of fungal distribution and severity of infection in thirty-six mammal species
Carnivora	Nasua narica	Lainson and Shaw 1975	Pneumocystis and Histoplasma infection in wild animals from the Amazon region of Brazil
Chiroptera	Eumops glaucinus	Cavallini Sanches et al. 2013	Pneumocystis spp. in bats evaluated by qPCR

Chiroptera	Eumops glaucinus	Veloso et al. 2014	Pneumocystis spp. and Histoplasma capsulatum in bats lungs in Southern and Midwestern Regions of Brazil
Chiroptera	Molossus currentium	Cavallini Sanches et al. 2013	Pneumocystis spp. in bats evaluated by qPCR
Chiroptera	Molossus currentium	Veloso et al. 2014	Pneumocystis spp. and Histoplasma capsulatum in bats lungs in Southern and Midwestern Regions of Brazil
Chiroptera	Molossus rufus	Cavallini Sanches et al. 2013	Pneumocystis spp. in bats evaluated by qPCR
Chiroptera	Molossus rufus	Veloso et al. 2014	Pneumocystis spp. and Histoplasma capsulatum in bats lungs in Southern and Midwestern Regions of Brazil
Chiroptera	Nyctinomops macrotis	Cavallini Sanches et al. 2013	Pneumocystis spp. in bats evaluated by qPCR
Chiroptera	Nyctinomops macrotis	Veloso et al. 2014	Pneumocystis spp. and Histoplasma capsulatum in bats lungs in Southern and Midwestern Regions of Brazil
Chiroptera	Promops nasutus	Cavallini Sanches et al. 2013	Pneumocystis spp. in bats evaluated by qPCR
Chiroptera	Promops nasutus	Veloso et al. 2014	Pneumocystis spp. and Histoplasma capsulatum in bats lungs in Southern and Midwestern Regions of Brazil
Chiroptera	Mormoops megalophylla	Akbar et al. 2012	Characterizing Pneumocystis in the lungs of bats: understanding Pneumocystis evolution and the spread of Pneumocystis organisms in mammal populations
Chiroptera	Mormoops megalophylla	González-González et al. 2014	Histoplasma capsulatum and Pneumocystis spp. co-infection in wild bats from Argentina, French Guyana, and Mexico
Chiroptera	Pteronotus davyi	Akbar et al. 2012	Characterizing Pneumocystis in the lungs of bats: understanding Pneumocystis evolution and the spread of Pneumocystis organisms in mammal populations
Chiroptera	Pteronotus davyi	González-González et al. 2014	Histoplasma capsulatum and Pneumocystis spp. co-infection in wild bats from Argentina, French Guyana, and Mexico
Chiroptera	Pteronotus parnellii	Akbar et al. 2012	Characterizing Pneumocystis in the lungs of bats: understanding Pneumocystis evolution and the spread of Pneumocystis organisms in mammal populations
Chiroptera	Pteronotus parnellii	González-González et al. 2014	Histoplasma capsulatum and Pneumocystis spp. co-infection in wild bats from Argentina, French Guyana, and Mexico
Chiroptera	Natalus stramineus	Akbar et al. 2012	Characterizing Pneumocystis in the lungs of bats: understanding Pneumocystis evolution and the spread of Pneumocystis organisms in mammal populations

Chiroptera	Natalus stramineus	González-González et al. 2014	Histoplasma capsulatum and Pneumocystis spp. co-infection in wild bats from Argentina, French Guyana, and Mexico
Chiroptera	Artibeus fimbriatus	Cavallini Sanches et al. 2013	Pneumocystis spp. in bats evaluated by qPCR
Chiroptera	Artibeus fimbriatus	Sanches et al. 2009	Detection of Pneumocystis in lungs of bats from Brazil by PCR amplification
Chiroptera	Artibeus fimbriatus	Veloso et al. 2014	Pneumocystis spp. and Histoplasma capsulatum in bats lungs in Southern and Midwestern Regions of Brazil
Chiroptera	Artibeus hirsutus	Akbar et al. 2012	Characterizing Pneumocystis in the lungs of bats: understanding Pneumocystis evolution and the spread of Pneumocystis organisms in mammal populations
Chiroptera	Artibeus hirsutus	González-González et al. 2014	Histoplasma capsulatum and Pneumocystis spp. co-infection in wild bats from Argentina, French Guyana, and Mexico
Chiroptera	Artibeus lituratus	Cavallini Sanches et al. 2013	Pneumocystis spp. in bats evaluated by qPCR
Chiroptera	Artibeus lituratus	Veloso et al. 2014	Pneumocystis spp. and Histoplasma capsulatum in bats lungs in Southern and Midwestern Regions of Brazil
Chiroptera	Artibeus spp.	Cavallini Sanches et al. 2013	Pneumocystis spp. in bats evaluated by qPCR
Chiroptera	Artibeus spp.	Veloso et al. 2014	Pneumocystis spp. and Histoplasma capsulatum in bats lungs in Southern and Midwestern Regions of Brazil
Chiroptera	Carollia perspicillata	Akbar et al. 2012	Characterizing Pneumocystis in the lungs of bats: understanding Pneumocystis evolution and the spread of Pneumocystis organisms in mammal populations
Chiroptera	Carollia perspicillata	Cavallini Sanches et al. 2013	Pneumocystis spp. in bats evaluated by qPCR
Chiroptera	Carollia perspicillata	González-González et al. 2014	Histoplasma capsulatum and Pneumocystis spp. co-infection in wild bats from Argentina, French Guyana, and Mexico
Chiroptera	Carollia perspicillata	Veloso et al. 2014	Pneumocystis spp. and Histoplasma capsulatum in bats lungs in Southern and Midwestern Regions of Brazil
Chiroptera	Diaemus youngii	Cavallini Sanches et al. 2013	Pneumocystis spp. in bats evaluated by qPCR
Chiroptera	Diaemus youngii	Veloso et al. 2014	Pneumocystis spp. and Histoplasma capsulatum in bats lungs in Southern and Midwestern Regions of Brazil
Chiroptera	Diphylla ecaudata	Sanches et al. 2009	Detection of Pneumocystis in lungs of bats from Brazil by PCR amplification

Chiroptera	Diphylla ecaudata	Veloso et al. 2014	Pneumocystis spp. and Histoplasma capsulatum in bats lungs in Southern and Midwestern Regions of Brazil
Chiroptera	Glossophaga soricina	Cavallini Sanches et al. 2013	Pneumocystis spp. in bats evaluated by qPCR
Chiroptera	Glossophaga soricina	Veloso et al. 2014	Pneumocystis spp. and Histoplasma capsulatum in bats lungs in Southern and Midwestern Regions of Brazil
Chiroptera	Rhinophylla pumilio	Veloso et al. 2014	Pneumocystis spp. and Histoplasma capsulatum in bats lungs in Southern and Midwestern Regions of Brazil
Chiroptera	Sturnira lilium	Cavallini Sanches et al. 2013	Pneumocystis spp. in bats evaluated by qPCR
Chiroptera	Sturnira lilium	Sanches et al. 2009	Detection of Pneumocystis in lungs of bats from Brazil by PCR amplification
Chiroptera	Sturnira lilium	Veloso et al. 2014	Pneumocystis spp. and Histoplasma capsulatum in bats lungs in Southern and Midwestern Regions of Brazil
Chiroptera	Rhinolophus hipposideros	Weissenbacher-Lang et al. 2023	Detection of Pneumocystis and morphological description of fungal distribution and severity of infection in thirty-six mammal species
Chiroptera	Aeorestes cinereus	Veloso et al. 2014	Pneumocystis spp. and Histoplasma capsulatum in bats lungs in Southern and Midwestern Regions of Brazil
Chiroptera	Eptesicus furinalis	Cavallini Sanches et al. 2013	Pneumocystis spp. in bats evaluated by qPCR
Chiroptera	Eptesicus furinalis	Veloso et al. 2014	Pneumocystis spp. and Histoplasma capsulatum in bats lungs in Southern and Midwestern Regions of Brazil
Chiroptera	Eptesicus serotinus	Akbar et al. 2012	Characterizing Pneumocystis in the lungs of bats: understanding Pneumocystis evolution and the spread of Pneumocystis organisms in mammal populations
Chiroptera	Eptesicus serotinus	Weissenbacher-Lang et al. 2023	Detection of Pneumocystis and morphological description of fungal distribution and severity of infection in thirty-six mammal species
Chiroptera	Hypsugo savii	Weissenbacher-Lang et al. 2023	Detection of Pneumocystis and morphological description of fungal distribution and severity of infection in thirty-six mammal species
Chiroptera	Lasiurus blossevillii	Cavallini Sanches et al. 2013	Pneumocystis spp. in bats evaluated by qPCR
Chiroptera	Lasiurus blossevillii	Veloso et al. 2014	Pneumocystis spp. and Histoplasma capsulatum in bats lungs in Southern and Midwestern Regions of Brazil
Chiroptera	Myotis californicus	Akbar et al. 2012	Characterizing Pneumocystis in the lungs of bats: understanding Pneumocystis evolution and the spread of Pneumocystis organisms in mammal populations

Chiroptera	Myotis californicus	González-González et al. 2014	Histoplasma capsulatum and Pneumocystis spp. co-infection in wild bats from Argentina, French Guyana, and Mexico
Chiroptera	Myotis daubentoni	Akbar et al. 2012	Characterizing Pneumocystis in the lungs of bats: understanding Pneumocystis evolution and the spread of Pneumocystis organisms in mammal populations
Chiroptera	Myotis levis	Cavallini Sanches et al. 2013	Pneumocystis spp. in bats evaluated by qPCR
Chiroptera	Myotis levis	Sanches et al. 2009	Detection of Pneumocystis in lungs of bats from Brazil by PCR amplification
Chiroptera	Myotis levis	Veloso et al. 2014	Pneumocystis spp. and Histoplasma capsulatum in bats lungs in Southern and Midwestern Regions of Brazil
Chiroptera	Myotis myotis	Akbar et al. 2012	Characterizing Pneumocystis in the lungs of bats: understanding Pneumocystis evolution and the spread of Pneumocystis organisms in mammal populations
Chiroptera	Myotis mystacinus	Weissenbacher-Lang et al. 2023	Detection of Pneumocystis and morphological description of fungal distribution and severity of infection in thirty-six mammal species
Chiroptera	Myotis nigricans	Cavallini Sanches et al. 2013	Pneumocystis spp. in bats evaluated by qPCR
Chiroptera	Myotis nigricans	Veloso et al. 2014	Pneumocystis spp. and Histoplasma capsulatum in bats lungs in Southern and Midwestern Regions of Brazil
Chiroptera	Nyctalus leisleri	Akbar et al. 2012	Characterizing Pneumocystis in the lungs of bats: understanding Pneumocystis evolution and the spread of Pneumocystis organisms in mammal populations
Chiroptera	Nyctalus noctula	Weissenbacher-Lang et al. 2023	Detection of Pneumocystis and morphological description of fungal distribution and severity of infection in thirty-six mammal species
Chiroptera	Pipistrellus pipistrellus	Akbar et al. 2012	Characterizing Pneumocystis in the lungs of bats: understanding Pneumocystis evolution and the spread of Pneumocystis organisms in mammal populations
Chiroptera	Pipistrellus pipistrellus	Derouiche et al. 2009	Pneumocystis diversity as a phyleographic tool
Chiroptera	Plecotus auritus	Akbar et al. 2012	Characterizing Pneumocystis in the lungs of bats: understanding Pneumocystis evolution and the spread of Pneumocystis organisms in mammal populations

Chiroptera	Plecotus austriacus	Akbar et al. 2012	Characterizing Pneumocystis in the lungs of bats: understanding Pneumocystis evolution and the spread of Pneumocystis organisms in mammal populations
Chiroptera	Vespertilio murinus	Weissenbacher-Lang et al. 2023	Detection of Pneumocystis and morphological description of fungal distribution and severity of infection in thirty-six mammal species
Chiroptera	Vespertilio spp.	Weissenbacher-Lang et al. 2023	Detection of Pneumocystis and morphological description of fungal distribution and severity of infection in thirty-six mammal species
Eulipotyphla	Atelerix albiventris	Weissenbacher-Lang et al. 2023	Detection of Pneumocystis and morphological description of fungal distribution and severity of infection in thirty-six mammal species
Eulipotyphla	Erinaceus europaeus	Settnes et al. 1986	Pneumocystis carinii Delanoe & Delanoe, 1912 found in lungs of freeliving animals in Denmark at Autopsy
Eulipotyphla	Erinaceus roumanicus	Weissenbacher-Lang et al. 2023	Detection of Pneumocystis and morphological description of fungal distribution and severity of infection in thirty-six mammal species
Eulipotyphla	Crocidura leucodon	Weissenbacher-Lang et al. 2023	Detection of Pneumocystis and morphological description of fungal distribution and severity of infection in thirty-six mammal species
Eulipotyphla	Crocidura suaveolens	Sebek and Rosicky 1967	The finding of Pneumocystis carinii in shrews (Incestivora:Soricidae)
Eulipotyphla	Crocidura suaveolens	Weissenbacher-Lang et al. 2023	Detection of Pneumocystis and morphological description of fungal distribution and severity of infection in thirty-six mammal species
Eulipotyphla	Neomys fodiens	Kučera et al. 1971	Pneumocystose
Eulipotyphla	Sorex araneus	Mazars et al. 1997	Detection of Pneumocystis in European wild animals
Eulipotyphla	Sorex araneus	Kučera et al. 1971	Pneumocystose
Eulipotyphla	Sorex araneus	Settnes and Lodal 1980	Prevalence of Pneumocystis carinii Delanoë & Delanoë, 1912 in rodents in Denmark
Eulipotyphla	Sorex isodon	Laakkonen 1998	Pneumocystis carinii in wildlife
Eulipotyphla	Sorex minutissimus	Laakkonen 1998	Pneumocystis carinii in wildlife
Eulipotyphla	Sorex minutus	Weissenbacher-Lang et al. 2023	Detection of Pneumocystis and morphological description of fungal distribution and severity of infection in thirty-six mammal species
Eulipotyphla	Sorex minutus	Kučera et al. 1971	Pneumocystose
Eulipotyphla	Sorex minutus	Laakkonen 1998	Pneumocystis carinii in wildlife
Eulipotyphla	Sorex minutus	Kučera et al. 1971	Pneumocystose
Eulipotyphla	Sorex minutus	Sebek and Rosicky 1967	The finding of Pneumocystis carinii in shrews (Incestivora:Soricidae)

Eulipotyphla	Sorex spp.	Peters et al. 1994	DNA analysis of Pneumocystis carinii infecting Finnish and English shrews
Eulipotyphla	Sorex spp.	Peters et al. 1994	DNA analysis of Pneumocystis carinii infecting Finnish and English shrews
Eulipotyphla	Talpa europaea	Mazars et al. 1997	Detection of Pneumocystis in European wild animals
Eulipotyphla	Talpa europaea	Weissenbacher-Lang et al. 2023	Detection of Pneumocystis and morphological description of fungal distribution and severity of infection in thirty-six mammal species
Pilosa	Bradypus tridactylus	Lainson and Shaw 1975	Pneumocystis and Histoplasma infections in wild animals from the Amazon region of Brazil
Pilosa	Bradypus variegatus	Yonushonis et al. 1986	Infection of a three-toed sloth (Bradypus variegatus) by a Pneumocystis-like organism in Panama
Pilosa	Choloepus didactylus	Lainson and Shaw 1975	Pneumocystis and Histoplasma infections in wild animals from the Amazon region of Brazil
Primates	Pithecia pithecia	Demanche et al. 2001	Phylogeny of Pneumocystis carinii from 18 primate species confirms host specificity and suggests coevolution
Rodentia	Heliophobius argenteocinereus	Petružela et al. 2019	Evolutionary history of Pneumocystis fungi in their African rodent hosts
Rodentia	Castor fiber	Weissenbacher-Lang et al. 2023	Detection of Pneumocystis and morphological description of fungal distribution and severity of infection in thirty-six mammal species
Rodentia	Arvicola terrestris	Settnes and Lodal 1980	Prevalence of Pneumocystis carinii Delanoë & Delanoë, 1912 in rodents in Denmark
Rodentia	Cricetus cricetus	Weissenbacher-Lang et al. 2023	Detection of Pneumocystis and morphological description of fungal distribution and severity of infection in thirty-six mammal species
Rodentia	Hylaeamys megacephalus	Lainson and Shaw 1975	Pneumocystis and Histoplasma infections in wild animals from the Amazon region of Brazil#
Rodentia	Microtus agrestis	Kučera et al. 1971	Pneumocystose
Rodentia	Microtus agrestis	Settnes and Lodal 1980	Prevalence of Pneumocystis carinii Delanoë & Delanoë, 1912 in rodents in Denmark
Rodentia	Microtus agrestis	Mazars et al. 1997	Detection of Pneumocystis in European wild animals
Rodentia	Microtus arvalis	Sebek and Rosicky 1967	The finding of Pneumocystis carinii in shrews (Incestivora:Soricidae)
Rodentia	Microtus multiplex	Danesi et al. 2016	Barcoding markers for Pneumocystis species in wildlife
Rodentia	Microtus subterraneus	Kučera et al. 1971	Pneumocystose

Rodentia	Myodes glareolus	Kučera et al. 1971	Pneumocystose
Rodentia	Myodes glareolus	Danesi et al. 2016	Barcoding markers for Pneumocystis species in wildlife
Rodentia	Myodes glareolus	Settnes and Lodal 1980	Prevalence of Pneumocystis carinii Delanoë & Delanoë, 1912 in rodents in Denmark
Rodentia	Myodes rufocanus	Shiota et al. 1986	Prevalence of Pneumocystis carinii in wild rodents in Japan
Rodentia	Peromyscus boylii	Laakkonen et al. 2001	Pneumocystis in wild small mammals from California
Rodentia	Peromyscus maniculatus	Laakkonen et al. 2001	Pneumocystis in wild small mammals from California
Rodentia	Eliomys quercinus	Mazars et al. 1997	Detection of Pneumocystis in European wild animals
Rodentia	Graphiurus spp.	Petružela et al. 2019	Evolutionary history of Pneumocystis fungi in their African rodent hosts
Rodentia	Muscardinus avellanarius	Sebek and Rosicky 1967	The finding of Pneumocystis carinii in shrews (Incestivora:Soricidae)
Rodentia	Chaetodipus californicus	Laakkonen et al. 2001	Pneumocystis in wild small mammals from California
Rodentia	Acomys ignitus	Petružela et al. 2019	Evolutionary history of Pneumocystis fungi in their African rodent hosts
Rodentia	Acomys muzei	Petružela et al. 2019	Evolutionary history of Pneumocystis fungi in their African rodent hosts
Rodentia	Acomys ngurui	Petružela et al. 2019	Evolutionary history of Pneumocystis fungi in their African rodent hosts
Rodentia	Aethomys hindei	Petružela et al. 2019	Evolutionary history of Pneumocystis fungi in their African rodent hosts
Rodentia	Aethomys kaiseri	Petružela et al. 2019	Evolutionary history of Pneumocystis fungi in their African rodent hosts
Rodentia	Apodemus flavicollis	Settnes and Lodal 1980	Prevalence of Pneumocystis carinii Delanoë & Delanoë, 1912 in rodents in Denmark
Rodentia	Apodemus sylvaticus	Settnes and Lodal 1980	Prevalence of Pneumocystis carinii Delanoë & Delanoë, 1912 in rodents in Denmark
Rodentia	Apomys banahao	Latinne et al. 2021	Revisiting the Pneumocystis host specificity paradigm and transmission ecology in wild Southeast Asian rodents
Rodentia	Berylmys bowersi	Latinne et al. 2021	Revisiting the Pneumocystis host specificity paradigm and transmission ecology in wild Southeast Asian rodents
Rodentia	Berylmys bowersi	Latinne et al. 2018	Genetic diversity and evolution of Pneumocystis fungi infecting wild Southeast Asian murid rodents

Rodentia	Grammomys surdaster	Petružela et al. 2019	Evolutionary history of Pneumocystis fungi in their African rodent hosts
Rodentia	Hylomyscus arcimontensis	Petružela et al. 2019	Evolutionary history of Pneumocystis fungi in their African rodent hosts
Rodentia	Lemniscomys rosalia	Petružela et al. 2019	Evolutionary history of Pneumocystis fungi in their African rodent hosts
Rodentia	Lemniscomys striatus	Petružela et al. 2019	Evolutionary history of Pneumocystis fungi in their African rodent hosts
Rodentia	Leopoldamys herberti	Latinne et al. 2018	Genetic diversity and evolution of Pneumocystis fungi infecting wild Southeast Asian murid rodents
Rodentia	Leopoldamys neilli	Latinne et al. 2021	Revisiting the Pneumocystis host specificity paradigm and transmission ecology in wild Southeast Asian rodents
Rodentia	Leopoldamys sabanus	Latinne et al. 2021	Revisiting the Pneumocystis host specificity paradigm and transmission ecology in wild Southeast Asian rodents
Rodentia	Lophuromys kilonzoi	Petružela et al. 2019	Evolutionary history of Pneumocystis fungi in their African rodent hosts
Rodentia	Lophuromys makundii	Petružela et al. 2019	Evolutionary history of Pneumocystis fungi in their African rodent hosts
Rodentia	Micromys minutus	Shiota et al. 1986	Prevalence of Pneumocystis carinii in wild rodents in Japan
Rodentia	Micromys minutus	Shiota et al. 1986	Prevalence of Pneumocystis carinii in wild rodents in Japan
Rodentia	Mus musculus	Shimizu et al. 1985	Occurrence of Pneumocystis carinii in animals in Japan
Rodentia	Mus musculus	Sebek and Rosicky 1967	The finding of Pneumocystis carinii in shrews (Incestivora:Soricidae)
Rodentia	Mus musculus	Shiota et al. 1986	Prevalence of Pneumocystis carinii in wild rodents in Japan
Rodentia	Mus pahari	Latinne et al. 2021	Revisiting the Pneumocystis host specificity paradigm and transmission ecology in wild Southeast Asian rodents
Rodentia	Myomyscus brockmani	Petružela et al. 2019	Evolutionary history of Pneumocystis fungi in their African rodent hosts
Rodentia	Niviventer fulvescens	Latinne et al. 2018	Genetic diversity and evolution of Pneumocystis fungi infecting wild Southeast Asian murid rodents
Rodentia	Otomys angoniensis	Petružela et al. 2019	Evolutionary history of Pneumocystis fungi in their African rodent hosts

Rodentia	Praomys delectorum	Petružela et al. 2019	Evolutionary history of Pneumocystis fungi in their African rodent hosts
Rodentia	Rattus andamanensis	Latinne et al. 2021	Revisiting the Pneumocystis host specificity paradigm and transmission ecology in wild Southeast Asian rodents
Rodentia	Rattus andamanensis	Latinne et al. 2018	Genetic diversity and evolution of Pneumocystis fungi infecting wild Southeast Asian murid rodents
Rodentia	Rattus everetti	Latinne et al. 2021	Revisiting the Pneumocystis host specificity paradigm and transmission ecology in wild Southeast Asian rodents
Rodentia	Rattus norvegicus	Shimizu et al. 1985	Occurrence of Pneumocystis carinii in animals in Japan
Rodentia	Rattus norvegicus	Kučera et al. 1971	Pneumocystose
Rodentia	Rattus norvegicus	Yoshida and Ikai 1979	Pneumocystis carinii pneumonia: epidemiology in Japan and cyst concentration method
Rodentia	Rattus rattus	Settnes and Lodal 1980	Prevalence of Pneumocystis carinii Delanoë & Delanoë, 1912 in rodents in Denmark
Rodentia	Rattus spp.	Shimizu et al. 1985	Occurrence of Pneumocystis carinii in animals in Japan
Rodentia	Rhabdomys dilectus	Petružela et al. 2019	Evolutionary history of Pneumocystis fungi in their African rodent hosts
Rodentia	Sundamys muelleri	Latinne et al. 2021	Revisiting the Pneumocystis host specificity paradigm and transmission ecology in wild Southeast Asian rodents
Rodentia	Myocastor coypus	Weissenbacher-Lang et al. 2023	Detection of Pneumocystis and morphological description of fungal distribution and severity of infection in thirty-six mammal species
Rodentia	Saccostomus umbriventer	Petružela et al. 2019	Evolutionary history of Pneumocystis fungi in their African rodent hosts
Rodentia	Steatomys ssp.	Petruzela et al. 2044	Evolutionary history of Pneumocystis fungi in their African rodent hosts
Rodentia	Sciurus vulgaris	Weissenbacher-Lang et al. 2023	Detection of Pneumocystis and morphological description of fungal distribution and severity of infection in thirty-six mammal species
Rodentia	Cannomys badius	Latinne et al. 2021	Revisiting the Pneumocystis host specificity paradigm and transmission ecology in wild Southeast Asian rodents
Rodentia	Cannomys badius	Latinne et al. 2018	Genetic diversity and evolution of Pneumocystis fungi infecting wild Southeast Asian murid rodents

Rodentia	Rhizomys pruinosus	Latinne et al. 2021	Revisiting the Pneumocystis host specificity paradigm and transmission ecology in wild Southeast Asian rodents
Rodentia	Tachyoryctes splendens	Petružela et al. 2019	Evolutionary history of Pneumocystis fungi in their African rodent hosts

No exact sample size indicated (n = 7)

Afrosoricida	Microgale spp.	Laakkonen 1998	Pneumocystis carinii in wildlife
Carnivora	Galictis vittata	Guillot et al. 2001	Parallel phylogenies of Pneumocystis species and their mammalian hosts
Chiroptera	Glossophaga soricina	Guillot et al. 2001	Parallel phylogenies of Pneumocystis species and their mammalian hosts
Didelphimorphia	Marmosa murina	Guillot et al. 2001	Parallel phylogenies of Pneumocystis species and their mammalian hosts
Rodentia	Agouti paca	Guillot et al. 2001	Parallel phylogenies of Pneumocystis species and their mammalian hosts
Rodentia	Proechimys guyannensis	Guillot et al. 2001	Parallel phylogenies of Pneumocystis species and their mammalian hosts
Rodentia	Sciurus aestuans	Guillot et al. 2001	Parallel phylogenies of Pneumocystis species and their mammalian hosts

Species not exactly defined (n = 5)

Rodentia	Peromyscus spp.	Salazar-Hamm et al. 2022	Breathing can be dangerous: Opportunistic fungal pathogens and the diverse community of the small mammal lung microbiome
Rodentia	Dipodomys spp.	Salazar-Hamm et al. 2022	Breathing can be dangerous: Opportunistic fungal pathogens and the diverse community of the small mammal lung microbiome
Rodentia	Apodemus spp.	Kučera et al. 1971	Pneumocystosis
Rodentia	Rattus rattus/Rattus norvegicus	Rothenburger et al. 2015	Respiratory Pathology and Pathogens in Wild Urban Rats (Rattus norvegicus and Rattus rattus)
Rodentia	Rattus spp.	Meer and Brug 1942	Infection a Pneumocystis chez l'homme et chez les animaux

Serological studies (n = 7)

Rodentia	Microtus agrestis	Kučera et al. 1971	Pneumocystosis
Rodentia	Microtus arvalis	Kučera et al. 1971	Pneumocystosis
Rodentia	Microtus subterraneus	Kučera et al. 1971	Pneumocystosis
Rodentia	Myodes glareolus	Kučera et al. 1971	Pneumocystosis
Rodentia	Apodemus flavicollis	Kučera et al. 1971	Pneumocystosis
Rodentia	Apodemus sylvaticus	Kučera et al. 1971	Pneumocystosis
Rodentia	Rattus rattus/Rattus norvegicus	Boey et al. 2019	Seroprevalence of Rodent Pathogens in Wild Rats from the Island of St. Kitts, West Indies

3. Level of infection.

Only negative results (n = 148)

Artiodactyla	Antilope spp.	Meer and Brug 1942	Infection a Pneumocystis chez l'homme et chez les animaux
Artiodactyla	Bos bonasus	Meer and Brug 1942	Infection a Pneumocystis chez l'homme et chez les animaux
Artiodactyla	Bos taurus	Yoshida and Ikai 1979	Pneumocystis carinii pneumonia: epidemiology in Japan and cyst concentration method
Artiodactyla	Capra hircus	Shimizu et al. 1985	Occurrence of Pneumocystis carinii in animals in Japan
Artiodactyla	Capra hircus	Settnes and Henriksen 1989	Pneumocystis carinii in large domestic animals in Denmark
Artiodactyla	Capra hircus	Shimizu et al. 1985	Occurrence of Pneumocystis carinii in animals in Japan
Artiodactyla	Capra hircus	Shimizu et al. 1985	Occurrence of Pneumocystis carinii in animals in Japan
Artiodactyla	Ovis aries	Meer and Brug 1942	Infection a Pneumocystis chez l'homme et chez les animaux
Artiodactyla	Camelus dromedarius	Weissenbacher-Lang et al. 2023	Detection of Pneumocystis and morphological description of fungal distribution and severity of infection in thirty-six mammal species
Artiodactyla	Lama glama	Underwood et al. 1992	Apparent retrovirus-induced immunosuppression in a yearling llama
Artiodactyla	Lama glama	Weissenbacher-Lang et al. 2023	Detection of Pneumocystis and morphological description of fungal distribution and severity of infection in thirty-six mammal species

Artiodactyla	Cervus alfredi	Weissenbacher-Lang et al. 2023	Detection of Pneumocystis and morphological description of fungal distribution and severity of infection in thirty-six mammal species
Artiodactyla	Cervus elaphus	Weissenbacher-Lang et al. 2023	Detection of Pneumocystis and morphological description of fungal distribution and severity of infection in thirty-six mammal species
Artiodactyla	Cervus nippon	Weissenbacher-Lang et al. 2023	Detection of Pneumocystis and morphological description of fungal distribution and severity of infection in thirty-six mammal species
Artiodactyla	Cervus spp.	Yoshida and Ikai 1979	Pneumocystis carinii pneumonia: epidemiology in Japan and cyst concentration method
Artiodactyla	Dama dama	Settnes and Henriksen 1989	Pneumocystis carinii in large domestic animals in Denmark
Artiodactyla	Elaphurus davidianus	Weissenbacher-Lang et al. 2023	Detection of Pneumocystis and morphological description of fungal distribution and severity of infection in thirty-six mammal species
Artiodactyla	Muntiacus muntjak	Meer and Brug 1942	Infection a Pneumocystis chez l'homme et chez les animaux
Artiodactyla	Rangifer tarandus	Weissenbacher-Lang et al. 2023	Detection of Pneumocystis and morphological description of fungal distribution and severity of infection in thirty-six mammal species
Artiodactyla	Rangifer tarandus groenlandicus	Settnes et al. 1986	Pneumocystis carinii Delanoe & Delanoe, 1912 found in lungs of freeliving animals in Denmark at Autopsy
Artiodactyla	Rusa unicolor	Meer and Brug 1942	Infection a Pneumocystis chez l'homme et chez les animaux
Artiodactyla	Sus scrofa	Yoshida and Ikai 1979	Pneumocystis carinii pneumonia: epidemiology in Japan and cyst concentration method
Artiodactyla	Sus scrofa domesticus	Yoshida and Ikai 1979	Pneumocystis carinii pneumonia: epidemiology in Japan and cyst concentration method
Artiodactyla	Sus scrofa domesticus	Davalos 1963	Latent infection produced by Pneumocystis carinii in domestic animals in Mexico
Carnivora	Canis lupus familiaris	Wöhrer et al. 2016	Age-related presence of selected viral and bacterial pathogens in paraffin-embedded lung samples of dogs with pneumonia
Carnivora	Vulpes vulpes	Weissenbacher-Lang et al. 2023	Detection of Pneumocystis and morphological description of fungal distribution and severity of infection in thirty-six mammal species
Carnivora	Vulpes vulpes	Yoshida and Ikai 1979	Pneumocystis carinii pneumonia: epidemiology in Japan and cyst concentration method
Carnivora	Vulpes vulpes	Danesi et al. 2016	Barcoding markers for Pneumocystis species in wildlife
Carnivora	Vulpes vulpes	Meer and Brug 1942	Infection a Pneumocystis chez l'homme et chez les animaux

Carnivora	Felis catus	Yoshida and Ikai 1979	Pneumocystis carinii pneumonia: epidemiology in Japan and cyst concentration method
Carnivora	Felis catus	Hagler et al. 1987	Feline leukemia virus and Pneumocystis carinii infection
Carnivora	Felis silvestris	Weissenbacher-Lang et al. 2023	Detection of Pneumocystis and morphological description of fungal distribution and severity of infection in thirty-six mammal species
Carnivora	Lynx lynx	Weissenbacher-Lang et al. 2023	Detection of Pneumocystis and morphological description of fungal distribution and severity of infection in thirty-six mammal species
Carnivora	Panthera leo	Weissenbacher-Lang et al. 2023	Detection of Pneumocystis and morphological description of fungal distribution and severity of infection in thirty-six mammal species
Carnivora	Panthera pardus	Weissenbacher-Lang et al. 2023	Detection of Pneumocystis and morphological description of fungal distribution and severity of infection in thirty-six mammal species
Carnivora	Panthera tigris	Weissenbacher-Lang et al. 2023	Detection of Pneumocystis and morphological description of fungal distribution and severity of infection in thirty-six mammal species
Carnivora	Puma concolor	Weissenbacher-Lang et al. 2023	Detection of Pneumocystis and morphological description of fungal distribution and severity of infection in thirty-six mammal species
Carnivora	Puma concolor	Meer and Brug 1942	Infection a Pneumocystis chez l'homme et chez les animaux
Carnivora	Martes foina	Settnes et al. 1986	Pneumocystis carinii Delanoe & Delanoe, 1912 found in lungs of freeliving animals in Denmark at Autopsy
Carnivora	Martes spp.	Weissenbacher-Lang et al. 2023	Detection of Pneumocystis and morphological description of fungal distribution and severity of infection in thirty-six mammal species
Carnivora	Meles meles	Settnes et al. 1986	Pneumocystis carinii Delanoe & Delanoe, 1912 found in lungs of freeliving animals in Denmark at Autopsy
Carnivora	Meles meles	Danesi et al. 2016	Barcoding markers for Pneumocystis species in wildlife
Carnivora	Mustela sibirica	Yoshida and Ikai 1979	Pneumocystis carinii pneumonia: epidemiology in Japan and cyst concentration method
Chiroptera	Eumops glaucinus	Cavallini Sanches et al. 2013	Pneumocystis spp. in bats evaluated by qPCR
Chiroptera	Mormoops megalophylla	Akbar et al. 2012	Characterizing Pneumocystis in the lungs of bats: understanding Pneumocystis evolution and the spread of Pneumocystis organisms in mammal populations
Chiroptera	Mormoops megalophylla	González-González et al. 2014	Histoplasma capsulatum and Pneumocystis spp. co-infection in wild bats from Argentina, French Guyana, and Mexico

Chiroptera	Pteronotus davyi	Akbar et al. 2012	Characterizing Pneumocystis in the lungs of bats: understanding Pneumocystis evolution and the spread of Pneumocystis organisms in mammal populations
Chiroptera	Pteronotus davyi	González-González et al. 2014	Histoplasma capsulatum and Pneumocystis spp. co-infection in wild bats from Argentina, French Guyana, and Mexico
Chiroptera	Pteronotus parnellii	González-González et al. 2014	Histoplasma capsulatum and Pneumocystis spp. co-infection in wild bats from Argentina, French Guyana, and Mexico
Chiroptera	Cynopterus spp.	Weissenbacher-Lang et al. 2023	Detection of Pneumocystis and morphological description of fungal distribution and severity of infection in thirty-six mammal species
Chiroptera	Pteropus giganteus	Weissenbacher-Lang et al. 2023	Detection of Pneumocystis and morphological description of fungal distribution and severity of infection in thirty-six mammal species
Chiroptera	Pteropus spp.	Weissenbacher-Lang et al. 2023	Detection of Pneumocystis and morphological description of fungal distribution and severity of infection in thirty-six mammal species
Chiroptera	Artibeus fimbriatus	Cavallini Sanches et al. 2013	Pneumocystis spp. in bats evaluated by qPCR
Chiroptera	Artibeus hirsutus	Akbar et al. 2012	Characterizing Pneumocystis in the lungs of bats: understanding Pneumocystis evolution and the spread of Pneumocystis organisms in mammal populations
Chiroptera	Artibeus hirsutus	González-González et al. 2014	Histoplasma capsulatum and Pneumocystis spp. co-infection in wild bats from Argentina, French Guyana, and Mexico
Chiroptera	Artibeus lituratus	Cavallini Sanches et al. 2013	Pneumocystis spp. in bats evaluated by qPCR
Chiroptera	Artibeus lituratus	Veloso et al. 2014	Pneumocystis spp. and Histoplasma capsulatum in bats lungs in Southern and Midwestern Regions of Brazil
Chiroptera	Artibeus spp.	Veloso et al. 2014	Pneumocystis spp. and Histoplasma capsulatum in bats lungs in Southern and Midwestern Regions of Brazil
Chiroptera	Carollia perspicillata	Akbar et al. 2012	Characterizing Pneumocystis in the lungs of bats: understanding Pneumocystis evolution and the spread of Pneumocystis organisms in mammal populations
Chiroptera	Carollia perspicillata	Cavallini Sanches et al. 2013	Pneumocystis spp. in bats evaluated by qPCR
Chiroptera	Carollia perspicillata	González-González et al. 2014	Histoplasma capsulatum and Pneumocystis spp. co-infection in wild bats from Argentina, French Guyana, and Mexico
Chiroptera	Diaemus youngii	Cavallini Sanches et al. 2013	Pneumocystis spp. in bats evaluated by qPCR

Chiroptera	Diaemus youngii	Veloso et al. 2014	Pneumocystis spp. and Histoplasma capsulatum in bats lungs in Southern and Midwestern Regions of Brazil
Chiroptera	Diphylla ecaudata	Veloso et al. 2014	Pneumocystis spp. and Histoplasma capsulatum in bats lungs in Southern and Midwestern Regions of Brazil
Chiroptera	Sturnira lilium	Cavallini Sanches et al. 2013	Pneumocystis spp. in bats evaluated by qPCR
Chiroptera	Sturnira lilium	Veloso et al. 2014	Pneumocystis spp. and Histoplasma capsulatum in bats lungs in Southern and Midwestern Regions of Brazil
Chiroptera	Rhinolophus hipposideros	Weissenbacher-Lang et al. 2023	Detection of Pneumocystis and morphological description of fungal distribution and severity of infection in thirty-six mammal species
Chiroptera	Eptesicus serotinus	Weissenbacher-Lang et al. 2023	Detection of Pneumocystis and morphological description of fungal distribution and severity of infection in thirty-six mammal species
Chiroptera	Hypsugo savii	Weissenbacher-Lang et al. 2023	Detection of Pneumocystis and morphological description of fungal distribution and severity of infection in thirty-six mammal species
Chiroptera	Myotis daubentoni	Akbar et al. 2012	Characterizing Pneumocystis in the lungs of bats: understanding Pneumocystis evolution and the spread of Pneumocystis organisms in mammal populations
Chiroptera	Myotis levis	Cavallini Sanches et al. 2013	Pneumocystis spp. in bats evaluated by qPCR
Chiroptera	Myotis myotis	Akbar et al. 2012	Characterizing Pneumocystis in the lungs of bats: understanding Pneumocystis evolution and the spread of Pneumocystis organisms in mammal populations
Chiroptera	Myotis mystacinus	Weissenbacher-Lang et al. 2023	Detection of Pneumocystis and morphological description of fungal distribution and severity of infection in thirty-six mammal species
Chiroptera	Nyctalus leisleri	Akbar et al. 2012	Characterizing Pneumocystis in the lungs of bats: understanding Pneumocystis evolution and the spread of Pneumocystis organisms in mammal populations
Chiroptera	Nyctalus noctula	Weissenbacher-Lang et al. 2023	Detection of Pneumocystis and morphological description of fungal distribution and severity of infection in thirty-six mammal species
Chiroptera	Vespertilio spp.	Weissenbacher-Lang et al. 2023	Detection of Pneumocystis and morphological description of fungal distribution and severity of infection in thirty-six mammal species
Eulipotyphla	Atelerix albiventris	Weissenbacher-Lang et al. 2023	Detection of Pneumocystis and morphological description of fungal distribution and severity of infection in thirty-six mammal species

Eulipotyphla	Erinaceus europaeus	Settnes et al. 1986	Pneumocystis carinii Delanoe & Delanoe, 1912 found in lungs of freeliving animals in Denmark at Autopsy
Eulipotyphla	Erinaceus europaeus	Weissenbacher-Lang et al. 2023	Detection of Pneumocystis and morphological description of fungal distribution and severity of infection in thirty-six mammal species
Eulipotyphla	Erinaceus roumanicus	Weissenbacher-Lang et al. 2023	Detection of Pneumocystis and morphological description of fungal distribution and severity of infection in thirty-six mammal species
Eulipotyphla	Blarina brevicauda	Laakkonen et al. 1997	Lung parasites of shrews from Pennsylvania
Eulipotyphla	Crocidura leucodon	Weissenbacher-Lang et al. 2023	Detection of Pneumocystis and morphological description of fungal distribution and severity of infection in thirty-six mammal species
Eulipotyphla	Crocidura suaveolens	Sebek and Rosicky 1967	The finding of Pneumocystis carinii in shrews (Incestivora:Soricidae)
Eulipotyphla	Crocidura suaveolens	Weissenbacher-Lang et al. 2023	Detection of Pneumocystis and morphological description of fungal distribution and severity of infection in thirty-six mammal species
Eulipotyphla	Sorex araneus	Settnes and Lodal 1980	Prevalence of Pneumocystis carinii Delanoë & Delanoë, 1912 in rodents in Denmark
Eulipotyphla	Sorex cinereus	Laakkonen et al. 1997	Lung parasites of shrews from Pennsylvania
Eulipotyphla	Sorex fumeus	Laakkonen et al. 1997	Lung parasites of shrews from Pennsylvania
Eulipotyphla	Sorex minutus	Weissenbacher-Lang et al. 2023	Detection of Pneumocystis and morphological description of fungal distribution and severity of infection in thirty-six mammal species
Eulipotyphla	Sorex minutus	Sebek and Rosicky 1967	The finding of Pneumocystis carinii in shrews (Incestivora:Soricidae)
Eulipotyphla	Sorex minutus	Laakkonen 1995	High prevalence of Pneumocystis carinii in Sorex araneus in Finland
Eulipotyphla	Talpa europaea	Weissenbacher-Lang et al. 2023	Detection of Pneumocystis and morphological description of fungal distribution and severity of infection in thirty-six mammal species
Eulipotyphla	Tupaia glis	Weissenbacher-Lang et al. 2023	Detection of Pneumocystis and morphological description of fungal distribution and severity of infection in thirty-six mammal species
Perissodactyla	Equus asinus	Weissenbacher-Lang et al. 2023	Detection of Pneumocystis and morphological description of fungal distribution and severity of infection in thirty-six mammal species
Perissodactyla	Equus quagga	Weissenbacher-Lang et al. 2023	Detection of Pneumocystis and morphological description of fungal distribution and severity of infection in thirty-six mammal species
Primates	Aotus trivirgatus	Weissenbacher-Lang et al. 2023	Detection of Pneumocystis and morphological description of fungal distribution and severity of infection in thirty-six mammal species
Primates	Callithrix jacchus	Weissenbacher-Lang et al. 2023	Detection of Pneumocystis and morphological description of fungal distribution and severity of infection in thirty-six mammal species

Primates	Leontocebus fuscicollis	Weissenbacher-Lang et al. 2023	Detection of Pneumocystis and morphological description of fungal distribution and severity of infection in thirty-six mammal species
Primates	Leontopithecus rosalia	Weissenbacher-Lang et al. 2023	Detection of Pneumocystis and morphological description of fungal distribution and severity of infection in thirty-six mammal species
Primates	Saguinus oedipus	Weissenbacher-Lang et al. 2023	Detection of Pneumocystis and morphological description of fungal distribution and severity of infection in thirty-six mammal species
Primates	Cebus capucinus	Weissenbacher-Lang et al. 2023	Detection of Pneumocystis and morphological description of fungal distribution and severity of infection in thirty-six mammal species
Primates	Saimiri sciureus	Weissenbacher-Lang et al. 2023	Detection of Pneumocystis and morphological description of fungal distribution and severity of infection in thirty-six mammal species
Primates	Cercopithecus/Miopithecus spp.	Weissenbacher-Lang et al. 2023	Detection of Pneumocystis and morphological description of fungal distribution and severity of infection in thirty-six mammal species
Primates	Colobus guereza	Weissenbacher-Lang et al. 2023	Detection of Pneumocystis and morphological description of fungal distribution and severity of infection in thirty-six mammal species
Primates	Colobus polykomos	Weissenbacher-Lang et al. 2023	Detection of Pneumocystis and morphological description of fungal distribution and severity of infection in thirty-six mammal species
Primates	Macaca mulatta	Weissenbacher-Lang et al. 2023	Detection of Pneumocystis and morphological description of fungal distribution and severity of infection in thirty-six mammal species
Primates	Macaca sylvanus	Weissenbacher-Lang et al. 2023	Detection of Pneumocystis and morphological description of fungal distribution and severity of infection in thirty-six mammal species
Primates	Theropithecus gelada	Weissenbacher-Lang et al. 2023	Detection of Pneumocystis and morphological description of fungal distribution and severity of infection in thirty-six mammal species
Primates	Pongo spp.	Weissenbacher-Lang et al. 2023	Detection of Pneumocystis and morphological description of fungal distribution and severity of infection in thirty-six mammal species
Primates	Eulemur macaco	Demanche et al. 2001	Phylogeny of Pneumocystis carinii from 18 primate species confirms host specificity and suggests coevolution
Primates	Pithecia pithecia	Weissenbacher-Lang et al. 2023	Detection of Pneumocystis and morphological description of fungal distribution and severity of infection in thirty-six mammal species
Rodentia	Castor fiber	Weissenbacher-Lang et al. 2023	Detection of Pneumocystis and morphological description of fungal distribution and severity of infection in thirty-six mammal species
Rodentia	Cavia porcellus	Shimizu et al. 1985	Occurrence of Pneumocystis carinii in animals in Japan

Rodentia	Kerodon rupestris	Weissenbacher-Lang et al. 2023	Detection of Pneumocystis and morphological description of fungal distribution and severity of infection in thirty-six mammal species
Rodentia	Arvicola terrestris	Settnes and Lodal 1980	Prevalence of Pneumocystis carinii Delanoë & Delanoë, 1912 in rodents in Denmark
Rodentia	Mesocricetus auratus	Weissenbacher-Lang et al. 2023	Detection of Pneumocystis and morphological description of fungal distribution and severity of infection in thirty-six mammal species
Rodentia	Microtus agrestis	Settnes and Lodal 1980	Prevalence of Pneumocystis carinii Delanoë & Delanoë, 1912 in rodents in Denmark
Rodentia	Microtus agrestis	Sebek and Rosicky 1967	The finding of Pneumocystis carinii in shrews (Incestivora:Soricidae)
Rodentia	Microtus arvalis	Sebek and Rosicky 1967	The finding of Pneumocystis carinii in shrews (Incestivora:Soricidae)
Rodentia	Microtus multiplex	Danesi et al. 2016	Barcoding markers for Pneumocystis species in wildlife
Rodentia	Microtus subterraneus	Sebek and Rosicky 1967	The finding of Pneumocystis carinii in shrews (Incestivora:Soricidae)
Rodentia	Myodes glareolus	Sebek and Rosicky 1967	The finding of Pneumocystis carinii in shrews (Incestivora:Soricidae)
Rodentia	Myodes glareolus	Settnes and Lodal 1980	Prevalence of Pneumocystis carinii Delanoë & Delanoë, 1912 in rodents in Denmark
Rodentia	Myodes rufocanus	Shiota et al. 1986	Prevalence of Pneumocystis carinii in wild rodents in Japan
Rodentia	Myodes smithi	Shiota et al. 1986	Prevalence of Pneumocystis carinii in wild rodents in Japan
Rodentia	Peromyscus boylii	Laakkonen et al. 2001	Pneumocystis in wild small mammals from California
Rodentia	Peromyscus californicus	Laakkonen et al. 2001	Pneumocystis in wild small mammals from California
Rodentia	Peromyscus maniculatus	Laakkonen et al. 2001	Pneumocystis in wild small mammals from California
Rodentia	Phodopus sungorus	Weissenbacher-Lang et al. 2023	Detection of Pneumocystis and morphological description of fungal distribution and severity of infection in thirty-six mammal species
Rodentia	Reithrodontomys megalotis	Laakkonen et al. 2001	Pneumocystis in wild small mammals from California
Rodentia	Muscardinus avellanarius	Sebek and Rosicky 1967	The finding of Pneumocystis carinii in shrews (Incestivora:Soricidae)
Rodentia	Apodemus agrarius	Laakkonen et al. 2006	Is there an association of Pneumocystis infection with the presence of arena-, hanta-, and poxvirus antibodies in wild mice and shrews in Finland?
Rodentia	Apodemus flavicollis	Settnes and Lodal 1980	Prevalence of Pneumocystis carinii Delanoë & Delanoë, 1912 in rodents in Denmark
Rodentia	Apodemus flavicollis	Sebek and Rosicky 1967	The finding of Pneumocystis carinii in shrews (Incestivora:Soricidae)
Rodentia	Apodemus sylvaticus	Sebek and Rosicky 1967	The finding of Pneumocystis carinii in shrews (Incestivora:Soricidae)

Rodentia	Meriones unguiculatus	Weissenbacher-Lang et al. 2023	Detection of Pneumocystis and morphological description of fungal distribution and severity of infection in thirty-six mammal species
Rodentia	Micromys minutus	Shiota et al. 1986	Prevalence of Pneumocystis carinii in wild rodents in Japan
Rodentia	Micromys minutus	Shiota et al. 1986	Prevalence of Pneumocystis carinii in wild rodents in Japan
Rodentia	Mus musculus	Sebek and Rosicky 1967	The finding of Pneumocystis carinii in shrews (Incestivora:Soricidae)
Rodentia	Mus musculus	Weissenbacher-Lang et al. 2023	Detection of Pneumocystis and morphological description of fungal distribution and severity of infection in thirty-six mammal species
Rodentia	Mus saxicola	Weissenbacher-Lang et al. 2023	Detection of Pneumocystis and morphological description of fungal distribution and severity of infection in thirty-six mammal species
Rodentia	Rattus everetti	Latinne et al. 2021	Revisiting the Pneumocystis host specificity paradigm and transmission ecology in wild Southeast Asian rodents
Rodentia	Rattus rattus	Settnes and Lodal 1980	Prevalence of Pneumocystis carinii Delanoë & Delanoë, 1912 in rodents in Denmark
Rodentia	Rattus spp.	Shimizu et al. 1985	Occurrence of Pneumocystis carinii in animals in Japan
Rodentia	Myocastor coypus	Weissenbacher-Lang et al. 2023	Detection of Pneumocystis and morphological description of fungal distribution and severity of infection in thirty-six mammal species
Rodentia	Octodon degus	Weissenbacher-Lang et al. 2023	Detection of Pneumocystis and morphological description of fungal distribution and severity of infection in thirty-six mammal species
Rodentia	Sciurus vulgaris	Weissenbacher-Lang et al. 2023	Detection of Pneumocystis and morphological description of fungal distribution and severity of infection in thirty-six mammal species
Rodentia	Tamias spp.	Weissenbacher-Lang et al. 2023	Detection of Pneumocystis and morphological description of fungal distribution and severity of infection in thirty-six mammal species
Rodentia	Tamiops swinhoei	Weissenbacher-Lang et al. 2023	Detection of Pneumocystis and morphological description of fungal distribution and severity of infection in thirty-six mammal species

Level of infection not indicated (n = 249)

Afrosoricida	Microgale spp.	Laakkonen 1998	Pneumocystis carinii in wildlife
Artiodactyla	Bos taurus	Shimizu et al. 1985	Occurrence of Pneumocystis carinii in animals in Japan
Artiodactyla	Bos taurus	Kučera et al. 1971	Pneumocystose
Artiodactyla	Bos taurus	Settnes and Henriksen 1989	Pneumocystis carinii in large domestic animals in Denmark

Artiodactyla	Ovis aries	Settnes and Henriksen 1989	Pneumocystis carinii in large domestic animals in Denmark
Artiodactyla	Capreolus capreolus	Settnes et al. 1986	Pneumocystis carinii Delanoe & Delanoe, 1912 found in lungs of freeliving animals in Denmark at Autopsy
Artiodactyla	Sus scrofa domesticus	Shimizu et al. 1985	Occurrence of Pneumocystis carinii in animals in Japan
Artiodactyla	Sus scrofa domesticus	Shimizu et al. 1985	Occurrence of Pneumocystis carinii in animals in Japan
Artiodactyla	Sus scrofa domesticus	Settnes and Henriksen 1989	Pneumocystis carinii in large domestic animals in Denmark
Artiodactyla	Sus scrofa domesticus	Kučera et al. 1971	Pneumocystose
Artiodactyla	Sus scrofa domesticus	Huang et al. 2022	A total infectome approach to understand the etiology of infectious disease in pigs
Artiodactyla	Sus scrofa domesticus	Esgalhado et al. 2013	Study of the epidemiology of Pneumocystis carinii f. sp. suis in abattoir swine in Portugal
Artiodactyla	Sus scrofa domesticus	Sanches et al. 2006	Co-Infection of Pneumocystis carinii f. sp. suis and Porcine Circovirus-2 (PCV2) in Pig Lungs Obtained from Slaughterhouses in Southern and Midwestern Regions of Brazil
Artiodactyla	Sus scrofa domesticus	Weissenbacher-Lang et al. 2023 2017	Comparison of Pneumocystis nucleic acid and antibody profiles and their associations with other respiratory pathogens in two Austrian pig herds
Artiodactyla	Sus scrofa domesticus	Sanches et al. 2007	Detection ofPneumocystisspp. in lung samples from pigs in Brazil
Artiodactyla	Sus scrofa domesticus	Sanches et al. 2011	Phylogenetic analysis of Pneumocystis from pig lungs obtained from slaughterhouses in southern and midwestern regions of Brazil
Carnivora	Ailurus fulgens	Poelma 1975	Pneumocystis carinii infections in zoo animals
Carnivora	Canis aureus	Kureljušic et al. 2022	First Molecular Detection of Pneumocystis spp. in the Golden Jackal (Canis aureus)
Carnivora	Canis lupus familiaris	English et al. 2001	DNA analysis of Pneumocystis infecting a Cavalier King Charles spaniel
Carnivora	Canis lupus familiaris	Merrill et al. 2021	X-linked CD40 ligand deficiency in a 1-year-old male Shih Tzu with secondary Pneumocystis pneumonia
Carnivora	Canis lupus familiaris	Yoshida and Ikai 1979	Pneumocystis carinii pneumonia: epidemiology in Japan and cyst concentration method
Carnivora	Canis lupus familiaris	Sukura et al. 1997	Occurrence of Pneumocystis carinii in canine distemper
Carnivora	Canis lupus familiaris	Brownlie 1990	A retrospective study of diagnosis in 109 cases of canine lower respiratory disease

Carnivora	Canis lupus familiaris	Davalos 1963	Latent infection produced by Pneumocystis carinii in domestic animals in Mexico
Carnivora	Canis lupus familiaris	Zavala and Rosado 1972	Pneumocystis carinii in domestic animals of the city of Mérida, Yucatán
Carnivora	Canis lupus familiaris	Cisse et al. 2021	Genomic insights into the host specific adaptation the Pneumocystis genus
Carnivora	Canis lupus familiaris	Petini et al. 2019	Nested–polymerase chain reaction detection of Pneumocystis carinii f. sp. canisin a suspected immunocompromised Cavalier King Charles spaniel with multiple infections
Carnivora	Nyctereutes procyonoides	Riebold et al. 2020	First molecular detection of Pneumocystis spp. in red foxes (Vulpes vulpes LINNAEUS, 1758) and raccoon dogs (Nyctereutes procyonoides GRAY, 1834)
Carnivora	Vulpes vulpes	Settnes et al. 1986	Pneumocystis carinii Delanoe & Delanoe, 1912 found in lungs of freeliving animals in Denmark at Autopsy
Carnivora	Vulpes vulpes	Riebold et al. 2020	First molecular detection of Pneumocystis spp. in red foxes (Vulpes vulpes LINNAEUS, 1758) and raccoon dogs (Nyctereutes procyonoides GRAY, 1834)
Carnivora	Vulpes zerda	Poelma 1975	Pneumocystis carinii infections in zoo animals
Carnivora	Felis catus	Zavala and Rosado 1972	Pneumocystis carinii in domestic animals of the city of Mérida, Yucatán
Carnivora	Felis catus	Danesi et al. 2019	Molecular detection of Pneumocystis in the lungs of cats
Carnivora	Felis catus	Davalos 1963	Latent infection produced by Pneumocystis carinii in domestic animals in Mexico
Carnivora	Galictis vittata	Guillot et al. 2001	Parallel phylogenies of Pneumocystis species and their mammalian hosts
Chiroptera	Eumops glaucinus	Veloso et al. 2014	Pneumocystis spp. and Histoplasma capsulatum in bats lungs in Southern and Midwestern Regions of Brazil
Chiroptera	Molossus currentium	Veloso et al. 2014	Pneumocystis spp. and Histoplasma capsulatum in bats lungs in Southern and Midwestern Regions of Brazil
Chiroptera	Molossus molossus	Veloso et al. 2014	Pneumocystis spp. and Histoplasma capsulatum in bats lungs in Southern and Midwestern Regions of Brazil

Chiroptera	Molossus molossus	Sanches et al. 2009	Detection of Pneumocystis in lungs of bats from Brazil by PCR amplification
Chiroptera	Molossus rufus	Veloso et al. 2014	Pneumocystis spp. and Histoplasma capsulatum in bats lungs in Southern and Midwestern Regions of Brazil
Chiroptera	Nyctinomops laticaudatus	Sanches et al. 2009	Detection of Pneumocystis in lungs of bats from Brazil by PCR amplification
Chiroptera	Nyctinomops laticaudatus	Veloso et al. 2014	Pneumocystis spp. and Histoplasma capsulatum in bats lungs in Southern and Midwestern Regions of Brazil
Chiroptera	Nyctinomops macrotis	Veloso et al. 2014	Pneumocystis spp. and Histoplasma capsulatum in bats lungs in Southern and Midwestern Regions of Brazil
Chiroptera	Promops nasutus	Veloso et al. 2014	Pneumocystis spp. and Histoplasma capsulatum in bats lungs in Southern and Midwestern Regions of Brazil
Chiroptera	Tadarida brasiliensis	Sanches et al. 2009	Detection of Pneumocystis in lungs of bats from Brazil by PCR amplification
Chiroptera	Tadarida brasiliensis	Akbar et al. 2012	Characterizing Pneumocystis in the lungs of bats: understanding Pneumocystis evolution and the spread of Pneumocystis organisms in mammal populations
Chiroptera	Tadarida brasiliensis	Derouiche et al. 2009	Pneumocystis diversity as a phyleographic tool
Chiroptera	Tadarida brasiliensis	González-González et al. 2014	Histoplasma capsulatum and Pneumocystis spp. co-infection in wild bats from Argentina, French Guyana, and Mexico
Chiroptera	Tadarida brasiliensis	Veloso et al. 2014	Pneumocystis spp. and Histoplasma capsulatum in bats lungs in Southern and Midwestern Regions of Brazil
Chiroptera	Pteronotus parnellii	Akbar et al. 2012	Characterizing Pneumocystis in the lungs of bats: understanding Pneumocystis evolution and the spread of Pneumocystis organisms in mammal populations
Chiroptera	Natalus stramineus	Akbar et al. 2012	Characterizing Pneumocystis in the lungs of bats: understanding Pneumocystis evolution and the spread of Pneumocystis organisms in mammal populations
Chiroptera	Natalus stramineus	González-González et al. 2014	Histoplasma capsulatum and Pneumocystis spp. co-infection in wild bats from Argentina, French Guyana, and Mexico

Chiroptera	Pteropus rodricensis	Akbar et al. 2012	Characterizing Pneumocystis in the lungs of bats: understanding Pneumocystis evolution and the spread of Pneumocystis organisms in mammal populations
Chiroptera	Rousettus aegyptiacus	Akbar et al. 2012	Characterizing Pneumocystis in the lungs of bats: understanding Pneumocystis evolution and the spread of Pneumocystis organisms in mammal populations
Chiroptera	Artibeus fimbriatus	Sanches et al. 2009	Detection of Pneumocystis in lungs of bats from Brazil by PCR amplification
Chiroptera	Artibeus fimbriatus	Veloso et al. 2014	Pneumocystis spp. and Histoplasma capsulatum in bats lungs in Southern and Midwestern Regions of Brazil
Chiroptera	Carollia perspicillata	Veloso et al. 2014	Pneumocystis spp. and Histoplasma capsulatum in bats lungs in Southern and Midwestern Regions of Brazil
Chiroptera	Desmodus rotundus	Sanches et al. 2009	Detection of Pneumocystis in lungs of bats from Brazil by PCR amplification
Chiroptera	Desmodus rotundus	Veloso et al. 2014	Pneumocystis spp. and Histoplasma capsulatum in bats lungs in Southern and Midwestern Regions of Brazil
Chiroptera	Diphylla ecaudata	Sanches et al. 2009	Detection of Pneumocystis in lungs of bats from Brazil by PCR amplification
Chiroptera	Glossophaga soricina	Guillot et al. 2001	Parallel phylogenies of Pneumocystis species and their mammalian hosts
Chiroptera	Glossophaga soricina	Akbar et al. 2012	Characterizing Pneumocystis in the lungs of bats: understanding Pneumocystis evolution and the spread of Pneumocystis organisms in mammal populations
Chiroptera	Glossophaga soricina	Derouiche et al. 2009	Pneumocystis diversity as a phyleographic tool
Chiroptera	Glossophaga soricina	González-González et al. 2014	Histoplasma capsulatum and Pneumocystis spp. co-infection in wild bats from Argentina, French Guyana, and Mexico
Chiroptera	Glossophaga soricina	Veloso et al. 2014	Pneumocystis spp. and Histoplasma capsulatum in bats lungs in Southern and Midwestern Regions of Brazil
Chiroptera	Rhinophylla pumilio	Veloso et al. 2014	Pneumocystis spp. and Histoplasma capsulatum in bats lungs in Southern and Midwestern Regions of Brazil
Chiroptera	Sturnira lilium	Sanches et al. 2009	Detection of Pneumocystis in lungs of bats from Brazil by PCR amplification

Chiroptera	Aeorestes cinereus	Veloso et al. 2014	Pneumocystis spp. and Histoplasma capsulatum in bats lungs in Southern and Midwestern Regions of Brazil
Chiroptera	Eptesicus furinalis	Veloso et al. 2014	Pneumocystis spp. and Histoplasma capsulatum in bats lungs in Southern and Midwestern Regions of Brazil
Chiroptera	Eptesicus serotinus	Akbar et al. 2012	Characterizing Pneumocystis in the lungs of bats: understanding Pneumocystis evolution and the spread of Pneumocystis organisms in mammal populations
Chiroptera	Histiotus velatus	Veloso et al. 2014	Pneumocystis spp. and Histoplasma capsulatum in bats lungs in Southern and Midwestern Regions of Brazil
Chiroptera	Lasiurus blossevillii	Veloso et al. 2014	Pneumocystis spp. and Histoplasma capsulatum in bats lungs in Southern and Midwestern Regions of Brazil
Chiroptera	Myotis californicus	Akbar et al. 2012	Characterizing Pneumocystis in the lungs of bats: understanding Pneumocystis evolution and the spread of Pneumocystis organisms in mammal populations
Chiroptera	Myotis californicus	González-González et al. 2014	Histoplasma capsulatum and Pneumocystis spp. co-infection in wild bats from Argentina, French Guyana, and Mexico
Chiroptera	Myotis levis	Sanches et al. 2009	Detection of Pneumocystis in lungs of bats from Brazil by PCR amplification
Chiroptera	Myotis levis	Veloso et al. 2014	Pneumocystis spp. and Histoplasma capsulatum in bats lungs in Southern and Midwestern Regions of Brazil
Chiroptera	Myotis nigricans	Veloso et al. 2014	Pneumocystis spp. and Histoplasma capsulatum in bats lungs in Southern and Midwestern Regions of Brazil
Chiroptera	Nyctalus noctula	Akbar et al. 2012	Characterizing Pneumocystis in the lungs of bats: understanding Pneumocystis evolution and the spread of Pneumocystis organisms in mammal populations
Chiroptera	Pipistrellus pipistrellus	Akbar et al. 2012	Characterizing Pneumocystis in the lungs of bats: understanding Pneumocystis evolution and the spread of Pneumocystis organisms in mammal populations
Chiroptera	Pipistrellus pipistrellus	Derouiche et al. 2009	Pneumocystis diversity as a phyleographic tool
Chiroptera	Plecotus auritus	Akbar et al. 2012	Characterizing Pneumocystis in the lungs of bats: understanding Pneumocystis evolution and the spread of Pneumocystis organisms in mammal populations

Chiroptera	Plecotus austriacus	Akbar et al. 2012	Characterizing Pneumocystis in the lungs of bats: understanding Pneumocystis evolution and the spread of Pneumocystis organisms in mammal populations
Didelphimorphia	Marmosa murina	Guillot et al. 2001	Parallel phylogenies of Pneumocystis species and their mammalian hosts
Diprotodontia	Osphranter rufus	Poelma 1975	Pneumocystis carinii infections in zoo animals
Eulipotyphla	Neomys fodiens	Kučera et al. 1971	Pneumocystose
Eulipotyphla	Sorex antinorii	Danesi et al. 2016	Barcoding markers for Pneumocystis species in wildlife
Eulipotyphla	Sorex araneus	Kučera et al. 1971	Pneumocystose
Eulipotyphla	Sorex araneus	Laakkonen 1998	Pneumocystis carinii in wildlife
Eulipotyphla	Sorex araneus	Laakkonen 1998	Pneumocystis carinii in wildlife
Eulipotyphla	Sorex araneus	Laakkonen et al. 1993	Pneumocystis carinii and helminth parasitism in shrews Sorex araneus and Sorex caecutiens
Eulipotyphla	Sorex caecutiens	Laakkonen et al. 1993	Pneumocystis carinii and helminth parasitism in shrews Sorex araneus and Sorex caecutiens
Eulipotyphla	Sorex isodon	Laakkonen 1998	Pneumocystis carinii in wildlife
Eulipotyphla	Sorex minutissimus	Laakkonen 1998	Pneumocystis carinii in wildlife
Eulipotyphla	Sorex minutus	Kučera et al. 1971	Pneumocystose
Eulipotyphla	Sorex minutus	Laakkonen 1998	Pneumocystis carinii in wildlife
Eulipotyphla	Sorex minutus	Kučera et al. 1971	Pneumocystose
Eulipotyphla	Sorex spp.	Peters et al. 1994	DNA analysis of Pneumocystis carinii infecting Finnish and English shrews
Eulipotyphla	Sorex spp.	Peters et al. 1994	DNA analysis of Pneumocystis carinii infecting Finnish and English shrews
Eulipotyphla	Tupaia glis	Poelma 1975	Pneumocystis carinii infections in zoo animals
Hyracoidea	Dendrohyrax arboreus	Poelma 1975	Pneumocystis carinii infections in zoo animals
Hyracoidea	Procavia capensis	Poelma 1975	Pneumocystis carinii infections in zoo animals
Lagomorpha	Lepus europaeus	Settnes et al. 1986	Pneumocystis carinii Delanoe & Delanoe, 1912 found in lungs of freeliving animals in Denmark at Autopsy
Lagomorpha	Oryctolagus cuniculus	Poelma 1975	Pneumocystis carinii infections in zoo animals

Lagomorpha	Oryctolagus cuniculus	Davalos 1963	Latent infection produced by Pneumocystis carinii in domestic animals in Mexico
Perissodactyla	Equus caballus	Clark-Price et al. 2004	Use of dapsone in the treatment of Pneumocystis carinii pneumonia in a foal
Perissodactyla	Equus caballus	Franklin et al. 2002	Proliferative interstitial pneumonia, Pneumocystis carinii infection, and immunodeficiency in an adult Paso Fino horse
Perissodactyla	Equus caballus	Prescott et al. 1991	Sporadic, severe bronchointerstitial pneumonia of foals
Perissodactyla	Equus caballus	Peters et al. 1994	Pneumocystis carinii pneumonia in thoroughbred foals: identification of a genetically distinct organism by DNA amplification
Pilosa	Bradypus tridactylus	Poelma 1975	Pneumocystis carinii infections in zoo animals
Primates	Alouatta fusca	Poelma 1975	Pneumocystis carinii infections in zoo animals
Primates	Ateles belzebuth	Poelma 1975	Pneumocystis carinii infections in zoo animals
Primates	Lagothrix lagothricha	Poelma 1975	Pneumocystis carinii infections in zoo animals
Primates	Callimico goeldii	Demanche et al. 2001	Phylogeny of Pneumocystis carinii from 18 primate species confirms host specificity and suggests coevolution
Primates	Callithrix aurita	Poelma 1975	Pneumocystis carinii infections in zoo animals
Primates	Callithrix geoffroyi	Demanche et al. 2001	Phylogeny of Pneumocystis carinii from 18 primate species confirms host specificity and suggests coevolution
Primates	Callithrix jacchus	Demanche et al. 2001	Phylogeny of Pneumocystis carinii from 18 primate species confirms host specificity and suggests coevolution
Primates	Saguinus fuscicollis	Demanche et al. 2001	Phylogeny of Pneumocystis carinii from 18 primate species confirms host specificity and suggests coevolution
Primates	Saguinus imperator	Demanche et al. 2001	Phylogeny of Pneumocystis carinii from 18 primate species confirms host specificity and suggests coevolution
Primates	Saguinus midas	Demanche et al. 2001	Phylogeny of Pneumocystis carinii from 18 primate species confirms host specificity and suggests coevolution
Primates	Saguinus oedipus	Demanche et al. 2001	Phylogeny of Pneumocystis carinii from 18 primate species confirms host specificity and suggests coevolution
Primates	Saimiri sciureus	Demanche et al. 2001	Phylogeny of Pneumocystis carinii from 18 primate species confirms host specificity and suggests coevolution

Primates	Allenopithecus nigroviridis	Demanche et al. 2001	Phylogeny of Pneumocystis carinii from 18 primate species confirms host specificity and suggests coevolution
Primates	Cercopithecus hamlyni	Demanche et al. 2001	Phylogeny of Pneumocystis carinii from 18 primate species confirms host specificity and suggests coevolution
Primates	Cercopithecus nictitans	Demanche et al. 2001	Phylogeny of Pneumocystis carinii from 18 primate species confirms host specificity and suggests coevolution
Primates	Macaca fascicularis	Demanche et al. 2001	Phylogeny of Pneumocystis carinii from 18 primate species confirms host specificity and suggests coevolution
Primates	Macaca fascicularis	Demanche et al. 2005	Molecular and serological evidence of Pneumocystis circulation in a social organization of healthy macaques (Macaca fascicularis)
Primates	Macaca fascicularis	Demanche et al. 2005	Molecular and serological evidence of Pneumocystis circulation in a social organization of healthy macaques (Macaca fascicularis)
Primates	Macaca mulatta	Demanche et al. 2001	Phylogeny of Pneumocystis carinii from 18 primate species confirms host specificity and suggests coevolution
Primates	Macaca nemestrina	Demanche et al. 2001	Phylogeny of Pneumocystis carinii from 18 primate species confirms host specificity and suggests coevolution
Primates	Galago demidoff	Poelma 1975	Pneumocystis carinii infections in zoo animals
Primates	Galago senegalensis	Poelma 1975	Pneumocystis carinii infections in zoo animals
Primates	Pan troglodytes	Poelma 1975	Pneumocystis carinii infections in zoo animals
Primates	Hapalemur griseus	Demanche et al. 2001	Phylogeny of Pneumocystis carinii from 18 primate species confirms host specificity and suggests coevolution
Primates	Pithecia pithecia	Demanche et al. 2001	Phylogeny of Pneumocystis carinii from 18 primate species confirms host specificity and suggests coevolution
Rodentia	Heliophobius argenteocinereus	Petružela et al. 2019	Evolutionary history of Pneumocystis fungi in their African rodent hosts
Rodentia	Microtus agrestis	Kučera et al. 1971	Pneumocystose
Rodentia	Microtus agrestis	Kučera et al. 1971	Pneumocystose
Rodentia	Microtus agrestis	Kučera et al. 1971	Pneumocystose
Rodentia	Microtus arvalis	Kučera et al. 1971	Pneumocystose
Rodentia	Microtus arvalis	Kučera et al. 1971	Pneumocystose
Rodentia	Microtus subterraneus	Kučera et al. 1971	Pneumocystose
Rodentia	Microtus subterraneus	Kučera et al. 1971	Pneumocystose

Rodentia	Myodes glareolus	Kučera et al. 1971	Pneumocystose
Rodentia	Myodes glareolus	Kučera et al. 1971	Pneumocystose
Rodentia	Myodes glareolus	Kučera et al. 1971	Pneumocystose
Rodentia	Myodes glareolus	Danesi et al. 2016	Barcoding markers for Pneumocystis species in wildlife
Rodentia	Peromyscus spp.	Salazar-Hamm et al. 2022	Breathing can be dangerous: Opportunistic fungal pathogens and the diverse community of the small mammal lung microbiome
Rodentia	Agouti paca	Guillot et al. 2001	Parallel phylogenies of Pneumocystis species and their mammalian hosts
Rodentia	Proechimys guyannensis	Guillot et al. 2001	Parallel phylogenies of Pneumocystis species and their mammalian hosts
Rodentia	Graphiurus murinus	Petružela et al. 2019	Evolutionary history of Pneumocystis fungi in their African rodent hosts
Rodentia	Graphiuru ssp.	Petružela et al. 2019	Evolutionary history of Pneumocystis fungi in their African rodent hosts
Rodentia	Dipodomys spp.	Salazar-Hamm et al. 2022	Breathing can be dangerous: Opportunistic fungal pathogens and the diverse community of the small mammal lung microbiome
Rodentia	Acomys ignitus	Petružela et al. 2019	Evolutionary history of Pneumocystis fungi in their African rodent hosts
Rodentia	Acomys muzei	Petružela et al. 2019	Evolutionary history of Pneumocystis fungi in their African rodent hosts
Rodentia	Acomys ngurui	Petružela et al. 2019	Evolutionary history of Pneumocystis fungi in their African rodent hosts
Rodentia	Acomys wilsoni	Petružela et al. 2019	Evolutionary history of Pneumocystis fungi in their African rodent hosts
Rodentia	Aethomys chrysophilus	Petružela et al. 2019	Evolutionary history of Pneumocystis fungi in their African rodent hosts
Rodentia	Aethomys hindei	Petružela et al. 2019	Evolutionary history of Pneumocystis fungi in their African rodent hosts
Rodentia	Aethomys kaiseri	Petružela et al. 2019	Evolutionary history of Pneumocystis fungi in their African rodent hosts
Rodentia	Apodemus flavicollis	Danesi et al. 2016	Barcoding markers for Pneumocystis species in wildlife
Rodentia	Apodemus flavicollis	Kučera et al. 1971	Pneumocystose

Rodentia	Apodemus sylvaticus	Kučera et al. 1971	Pneumocystose
Rodentia	Apodemus sylvaticus	Settnes and Lodal 1980	Prevalence of Pneumocystis carinii Delanoë & Delanoë, 1912 in rodents in Denmark
Rodentia	Apodemus sylvaticus	Demanche et al. 2015	What Do Pneumocystis Organisms Tell Us about the Phylogeography of Their Hosts? The Case of the Woodmouse Apodemus sylvaticus in Continental Europe and Western Mediterranean Islands
Rodentia	Apodemus spp.	Kučera et al. 1971	Pneumocystose
Rodentia	Apomys banahao	Latinne et al. 2021	Revisiting the Pneumocystis host specificity paradigm and transmission ecology in wild Southeast Asian rodents
Rodentia	Bandicota indica	Latinne et al. 2021	Revisiting the Pneumocystis host specificity paradigm and transmission ecology in wild Southeast Asian rodents
Rodentia	Bandicota indica	Latinne et al. 2018	Genetic diversity and evolution of Pneumocystis fungi infecting wild Southeast Asian murid rodents
Rodentia	Bandicota savilei	Latinne et al. 2021	Revisiting the Pneumocystis host specificity paradigm and transmission ecology in wild Southeast Asian rodents
Rodentia	Bandicota savilei	Latinne et al. 2018	Genetic diversity and evolution of Pneumocystis fungi infecting wild Southeast Asian murid rodents
Rodentia	Berylmys berdmorei	Latinne et al. 2021	Revisiting the Pneumocystis host specificity paradigm and transmission ecology in wild Southeast Asian rodents
Rodentia	Berylmys berdmorei	Latinne et al. 2018	Genetic diversity and evolution of Pneumocystis fungi infecting wild Southeast Asian murid rodents
Rodentia	Berylmys bowersi	Latinne et al. 2021	Revisiting the Pneumocystis host specificity paradigm and transmission ecology in wild Southeast Asian rodents
Rodentia	Berylmys bowersi	Latinne et al. 2018	Genetic diversity and evolution of Pneumocystis fungi infecting wild Southeast Asian murid rodents
Rodentia	Gerbilliscus vicinus	Petružela et al. 2019	Evolutionary history of Pneumocystis fungi in their African rodent hosts
Rodentia	Grammomys surdaster	Petružela et al. 2019	Evolutionary history of Pneumocystis fungi in their African rodent hosts
Rodentia	Hylomyscus arcimontensis	Petružela et al. 2019	Evolutionary history of Pneumocystis fungi in their African rodent hosts

Rodentia	Lemniscomys rosalia	Petružela et al. 2019	Evolutionary history of Pneumocystis fungi in their African rodent hosts
Rodentia	Lemniscomys striatus	Petružela et al. 2019	Evolutionary history of Pneumocystis fungi in their African rodent hosts
Rodentia	Leopoldamys herberti	Latinne et al. 2021	Revisiting the Pneumocystis host specificity paradigm and transmission ecology in wild Southeast Asian rodents
Rodentia	Leopoldamys herberti	Latinne et al. 2018	Genetic diversity and evolution of Pneumocystis fungi infecting wild Southeast Asian murid rodents
Rodentia	Leopoldamys neilli	Latinne et al. 2021	Revisiting the Pneumocystis host specificity paradigm and transmission ecology in wild Southeast Asian rodents
Rodentia	Leopoldamys sabanus	Latinne et al. 2021	Revisiting the Pneumocystis host specificity paradigm and transmission ecology in wild Southeast Asian rodents
Rodentia	Lophuromys kilonzoi	Petružela et al. 2019	Evolutionary history of Pneumocystis fungi in their African rodent hosts
Rodentia	Lophuromys makundii	Petružela et al. 2019	Evolutionary history of Pneumocystis fungi in their African rodent hosts
Rodentia	Mastomys natalensis	Petružela et al. 2019	Evolutionary history of Pneumocystis fungi in their African rodent hosts
Rodentia	Maxomys surifer	Latinne et al. 2021	Revisiting the Pneumocystis host specificity paradigm and transmission ecology in wild Southeast Asian rodents
Rodentia	Maxomys surifer	Latinne et al. 2018	Genetic diversity and evolution of Pneumocystis fungi infecting wild Southeast Asian murid rodents
Rodentia	Mus caroli	Latinne et al. 2018	Genetic diversity and evolution of Pneumocystis fungi infecting wild Southeast Asian murid rodents
Rodentia	Mus caroli	Latinne et al. 2021	Revisiting the Pneumocystis host specificity paradigm and transmission ecology in wild Southeast Asian rodents
Rodentia	Mus cervicolor	Latinne et al. 2021	Revisiting the Pneumocystis host specificity paradigm and transmission ecology in wild Southeast Asian rodents
Rodentia	Mus cervicolor	Latinne et al. 2018	Genetic diversity and evolution of Pneumocystis fungi infecting wild Southeast Asian murid rodents
Rodentia	Mus cookii	Latinne et al. 2018	Genetic diversity and evolution of Pneumocystis fungi infecting wild Southeast Asian murid rodents

Rodentia	Mus cookii	Latinne et al. 2021	Revisiting the Pneumocystis host specificity paradigm and transmission ecology in wild Southeast Asian rodents
Rodentia	Mus minutoides	Petružela et al. 2019	Evolutionary history of Pneumocystis fungi in their African rodent hosts
Rodentia	Mus musculus	Shimizu et al. 1985	Occurrence of Pneumocystis carinii in animals in Japan
Rodentia	Mus musculus	Kučera et al. 1971	Pneumocystose
Rodentia	Mus musculus	Settnes and Lodal 1980	Prevalence of Pneumocystis carinii Delanoë & Delanoë, 1912 in rodents in Denmark
Rodentia	Mus musculus	Porter 1915	The occurrence of Pneumocystis carinii in mice in England
Rodentia	Mus musculus	Hayashimoto et al. 2014	Microbiological survey of mice (Mus musculus) purchased from commercial pet shops in Kanagawa and Tokyo, Japan
Rodentia	Mus musculus	Bellocq et al. 2018	Holobiont suture zones: Parasite evidence across the European house mouse hybrid zone
Rodentia	Mus pahari	Latinne et al. 2021	Revisiting the Pneumocystis host specificity paradigm and transmission ecology in wild Southeast Asian rodents
Rodentia	Mus triton	Petružela et al. 2019	Evolutionary history of Pneumocystis fungi in their African rodent hosts
Rodentia	Myomyscus brockmani	Petružela et al. 2019	Evolutionary history of Pneumocystis fungi in their African rodent hosts
Rodentia	Niviventer fulvescens	Latinne et al. 2021	Revisiting the Pneumocystis host specificity paradigm and transmission ecology in wild Southeast Asian rodents
Rodentia	Niviventer fulvescens	Latinne et al. 2018	Genetic diversity and evolution of Pneumocystis fungi infecting wild Southeast Asian murid rodents
Rodentia	Otomys angoniensis	Petružela et al. 2019	Evolutionary history of Pneumocystis fungi in their African rodent hosts
Rodentia	Praomys delectorum	Petružela et al. 2019	Evolutionary history of Pneumocystis fungi in their African rodent hosts
Rodentia	Rattus andamanensis	Latinne et al. 2021	Revisiting the Pneumocystis host specificity paradigm and transmission ecology in wild Southeast Asian rodents
Rodentia	Rattus andamanensis	Latinne et al. 2018	Genetic diversity and evolution of Pneumocystis fungi infecting wild Southeast Asian murid rodents

Rodentia	Rattus argentiventer	Latinne et al. 2021	Revisiting the Pneumocystis host specificity paradigm and transmission ecology in wild Southeast Asian rodents
Rodentia	Rattus argentiventer	Latinne et al. 2018	Genetic diversity and evolution of Pneumocystis fungi infecting wild Southeast Asian murid rodents
Rodentia	Rattus exulans	Latinne et al. 2021	Revisiting the Pneumocystis host specificity paradigm and transmission ecology in wild Southeast Asian rodents
Rodentia	Rattus exulans	Latinne et al. 2018	Genetic diversity and evolution of Pneumocystis fungi infecting wild Southeast Asian murid rodents
Rodentia	Rattus nitidus	Latinne et al. 2021	Revisiting the Pneumocystis host specificity paradigm and transmission ecology in wild Southeast Asian rodents
Rodentia	Rattus nitidus	Latinne et al. 2018	Genetic diversity and evolution of Pneumocystis fungi infecting wild Southeast Asian murid rodents
Rodentia	Rattus norvegicus	Shimizu et al. 1985	Occurrence of Pneumocystis carinii in animals in Japan
Rodentia	Rattus norvegicus	Kučera et al. 1971	Pneumocystose
Rodentia	Rattus norvegicus	Settnes and Lodal 1980	Prevalence of Pneumocystis carinii Delanoë & Delanoë, 1912 in rodents in Denmark
Rodentia	Rattus norvegicus	Yoshida and Ikai 1979	Pneumocystis carinii pneumonia: epidemiology in Japan and cyst concentration method
Rodentia	Rattus norvegicus	Palmer et al. 2000	Population structure of rat-derived Pneumocystis carinii in Danish wild rats
Rodentia	Rattus norvegicus	Chabé et al. 2010	Pneumocystis carinii and Pneumocystis wakefieldiae in wild Rattus norvegicus trapped in Thailand
Rodentia	Rattus norvegicus	Latinne et al. 2021	Revisiting the Pneumocystis host specificity paradigm and transmission ecology in wild Southeast Asian rodents
Rodentia	Rattus norvegicus	Latinne et al. 2018	Genetic diversity and evolution of Pneumocystis fungi infecting wild Southeast Asian murid rodents
Rodentia	Rattus norvegicus	Yoshida and Ikai 1979	Pneumocystis carinii pneumonia: epidemiology in Japan and cyst concentration method
Rodentia	Rattus rattus	Shimizu et al. 1985	Occurrence of Pneumocystis carinii in animals in Japan
Rodentia	Rattus rattus	Shimizu et al. 1985	Occurrence of Pneumocystis carinii in animals in Japan
Rodentia	Rattus sakeratensis	Latinne et al. 2021	Revisiting the Pneumocystis host specificity paradigm and transmission ecology in wild Southeast Asian rodents

Rodentia	Rattus sakeratensis	Latinne et al. 2018	Genetic diversity and evolution of Pneumocystis fungi infecting wild Southeast Asian murid rodents
Rodentia	Rattus tanezumi	Latinne et al. 2021	Revisiting the Pneumocystis host specificity paradigm and transmission ecology in wild Southeast Asian rodents
Rodentia	Rattus tanezumi	Latinne et al. 2018	Genetic diversity and evolution of Pneumocystis fungi infecting wild Southeast Asian murid rodents
Rodentia	Rattus tanezumi	Latinne et al. 2021	Revisiting the Pneumocystis host specificity paradigm and transmission ecology in wild Southeast Asian rodents
Rodentia	Rattus tanezumi	Latinne et al. 2018	Genetic diversity and evolution of Pneumocystis fungi infecting wild Southeast Asian murid rodents
Rodentia	Rattus tiomanicus	Latinne et al. 2021	Revisiting the Pneumocystis host specificity paradigm and transmission ecology in wild Southeast Asian rodents
Rodentia	Rattus rattus/Rattus norvegicus	Rothenburger et al. 2015	Respiratory Pathology and Pathogens in Wild Urban Rats (Rattus norvegicus and Rattus rattus)
Rodentia	Rattus rattus/Rattus norvegicus	Boey et al. 2019	Seroprevalence of Rodent Pathogens in Wild Rats from the Island of St. Kitts, West Indies
Rodentia	Rhabdomys dilectus	Petružela et al. 2019	Evolutionary history of Pneumocystis fungi in their African rodent hosts
Rodentia	Sundamys muelleri	Latinne et al. 2021	Revisiting the Pneumocystis host specificity paradigm and transmission ecology in wild Southeast Asian rodents
Rodentia	Myocastor coypus	Danesi et al. 2016	Barcoding markers for Pneumocystis species in wildlife
Rodentia	Myocastor coypus	Danesi et al. 2018	Real-time PCR assay for screening Pneumocystis in free-living wild squirrels and river rats
Rodentia	Saccostomus umbriventer	Petružela et al. 2019	Evolutionary history of Pneumocystis fungi in their African rodent hosts
Rodentia	Steatomys spp.	Petružela et al. 2019	Evolutionary history of Pneumocystis fungi in their African rodent hosts
Rodentia	Callosciurus finlaysonii	Danesi et al. 2018	Real-time PCR assay for screening Pneumocystis in free-living wild squirrels and river rats
Rodentia	Funambulus palmarum	Poelma 1975	Pneumocystis carinii infections in zoo animals
Rodentia	Sciurus aestuans	Guillot et al. 2001	Parallel phylogenies of Pneumocystis species and their mammalian hosts

Rodentia	Cannomys badius	Latinne et al. 2021	Revisiting the Pneumocystis host specificity paradigm and transmission ecology in wild Southeast Asian rodents
Rodentia	Cannomys badius	Latinne et al. 2018	Genetic diversity and evolution of Pneumocystis fungi infecting wild Southeast Asian murid rodents
Rodentia	Rhizomys pruinosus	Latinne et al. 2021	Revisiting the Pneumocystis host specificity paradigm and transmission ecology in wild Southeast Asian rodents
Rodentia	Tachyoryctes splendens	Petružela et al. 2019	Evolutionary history of Pneumocystis fungi in their African rodent hosts

Species not exactly defined (n = 3)

Artiodactyla	Cervus spp.	Weissenbacher-Lang et al. 2023	Detection of Pneumocystis and morphological description of fungal distribution and severity of infection in thirty-six mammal species
Chiroptera	Artibeus spp.	Cavallini Sanches et al. 2013	Pneumocystis spp. in bats evaluated by qPCR
Rodentia	Rattus spp.	Meer and Brug 1942	Infection a Pneumocystis chez l'homme et chez les animaux

4. Level of infection in wild animals.

Only negative results (n = 88)

Artiodactyla	Cervus elaphus	Weissenbacher-Lang et al. 2023	Detection of Pneumocystis and morphological description of fungal distribution and severity of infection in thirty-six mammal species
Artiodactyla	Cervus spp.	Yoshida and Ikai 1979	Pneumocystis carinii pneumonia: epidemiology in Japan and cyst concentration method
Artiodactyla	Elaphurus davidianus	Weissenbacher-Lang et al. 2023	Detection of Pneumocystis and morphological description of fungal distribution and severity of infection in thirty-six mammal species
Artiodactyla	Rangifer tarandus	Weissenbacher-Lang et al. 2023	Detection of Pneumocystis and morphological description of fungal distribution and severity of infection in thirty-six mammal species
Artiodactyla	Rangifer tarandus groenlandicus	Settnes et al. 1986	Pneumocystis carinii Delanoe & Delanoe, 1912 found in lungs of freeliving animals in Denmark at Autopsy

Artiodactyla	Sus scrofa	Yoshida and Ikai 1979	Pneumocystis carinii pneumonia: epidemiology in Japan and cyst concentration method
Carnivora	Vulpes vulpes	Weissenbacher-Lang et al. 2023	Detection of Pneumocystis and morphological description of fungal distribution and severity of infection in thirty-six mammal species
Carnivora	Vulpes vulpes	Yoshida and Ikai 1979	Pneumocystis carinii pneumonia: epidemiology in Japan and cyst concentration method
Carnivora	Vulpes vulpes	Danesi et al. 2016	Barcoding markers for Pneumocystis species in wildlife
Carnivora	Vulpes vulpes	Meer and Brug 1942	Infection a Pneumocystis chez l'homme et chez les animaux
Carnivora	Martes foina	Settnes et al. 1986	Pneumocystis carinii Delanoe & Delanoe, 1912 found in lungs of freeliving animals in Denmark at Autopsy
Carnivora	Martes spp.	Weissenbacher-Lang et al. 2023	Detection of Pneumocystis and morphological description of fungal distribution and severity of infection in thirty-six mammal species
Carnivora	Meles meles	Settnes et al. 1986	Pneumocystis carinii Delanoe & Delanoe, 1912 found in lungs of freeliving animals in Denmark at Autopsy
Carnivora	Meles meles	Danesi et al. 2016	Barcoding markers for Pneumocystis species in wildlife
Carnivora	Mustela sibirica	Yoshida and Ikai 1979	Pneumocystis carinii pneumonia: epidemiology in Japan and cyst concentration method
Chiroptera	Eumops glaucinus	Cavallini Sanches et al. 2013	Pneumocystis spp. in bats evaluated by qPCR
Chiroptera	Mormoops megalophylla	Akbar et al. 2012	Characterizing Pneumocystis in the lungs of bats: understanding Pneumocystis evolution and the spread of Pneumocystis organisms in mammal populations
Chiroptera	Mormoops megalophylla	González-González et al. 2014	Histoplasma capsulatum and Pneumocystis spp. co-infection in wild bats from Argentina, French Guyana, and Mexico
Chiroptera	Pteronotus davyi	Akbar et al. 2012	Characterizing Pneumocystis in the lungs of bats: understanding Pneumocystis evolution and the spread of Pneumocystis organisms in mammal populations
Chiroptera	Pteronotus davyi	González-González et al. 2014	Histoplasma capsulatum and Pneumocystis spp. co-infection in wild bats from Argentina, French Guyana, and Mexico
Chiroptera	Pteronotus parnellii	González-González et al. 2014	Histoplasma capsulatum and Pneumocystis spp. co-infection in wild bats from Argentina, French Guyana, and Mexico
Chiroptera	Artibeus fimbriatus	Cavallini Sanches et al. 2013	Pneumocystis spp. in bats evaluated by qPCR

Chiroptera	Artibeus hirsutus	Akbar et al. 2012	Characterizing Pneumocystis in the lungs of bats: understanding Pneumocystis evolution and the spread of Pneumocystis organisms in mammal populations
Chiroptera	Artibeus hirsutus	González-González et al. 2014	Histoplasma capsulatum and Pneumocystis spp. co-infection in wild bats from Argentina, French Guyana, and Mexico
Chiroptera	Artibeus lituratus	Cavallini Sanches et al. 2013	Pneumocystis spp. in bats evaluated by qPCR
Chiroptera	Artibeus lituratus	Veloso et al. 2014	Pneumocystis spp. and Histoplasma capsulatum in bats lungs in Southern and Midwestern Regions of Brazil
Chiroptera	Artibeus spp.	Veloso et al. 2014	Pneumocystis spp. and Histoplasma capsulatum in bats lungs in Southern and Midwestern Regions of Brazil
Chiroptera	Carollia perspicillata	Akbar et al. 2012	Characterizing Pneumocystis in the lungs of bats: understanding Pneumocystis evolution and the spread of Pneumocystis organisms in mammal populations
Chiroptera	Carollia perspicillata	Cavallini Sanches et al. 2013	Pneumocystis spp. in bats evaluated by qPCR
Chiroptera	Carollia perspicillata	González-González et al. 2014	Histoplasma capsulatum and Pneumocystis spp. co-infection in wild bats from Argentina, French Guyana, and Mexico
Chiroptera	Diaemus youngii	Cavallini Sanches et al. 2013	Pneumocystis spp. in bats evaluated by qPCR
Chiroptera	Diaemus youngii	Veloso et al. 2014	Pneumocystis spp. and Histoplasma capsulatum in bats lungs in Southern and Midwestern Regions of Brazil
Chiroptera	Diphylla ecaudata	Veloso et al. 2014	Pneumocystis spp. and Histoplasma capsulatum in bats lungs in Southern and Midwestern Regions of Brazil
Chiroptera	Sturnira lilium	Cavallini Sanches et al. 2013	Pneumocystis spp. in bats evaluated by qPCR
Chiroptera	Sturnira lilium	Veloso et al. 2014	Pneumocystis spp. and Histoplasma capsulatum in bats lungs in Southern and Midwestern Regions of Brazil
Chiroptera	Rhinolophus hipposideros	Weissenbacher-Lang et al. 2023	Detection of Pneumocystis and morphological description of fungal distribution and severity of infection in thirty-six mammal species
Chiroptera	Eptesicus serotinus	Weissenbacher-Lang et al. 2023	Detection of Pneumocystis and morphological description of fungal distribution and severity of infection in thirty-six mammal species
Chiroptera	Hypsugo savii	Weissenbacher-Lang et al. 2023	Detection of Pneumocystis and morphological description of fungal distribution and severity of infection in thirty-six mammal species

Chiroptera	Myotis daubentoni	Akbar et al. 2012	Characterizing Pneumocystis in the lungs of bats: understanding Pneumocystis evolution and the spread of Pneumocystis organisms in mammal populations
Chiroptera	Myotis levis	Cavallini Sanches et al. 2013	Pneumocystis spp. in bats evaluated by qPCR
Chiroptera	Myotis myotis	Akbar et al. 2012	Characterizing Pneumocystis in the lungs of bats: understanding Pneumocystis evolution and the spread of Pneumocystis organisms in mammal populations
Chiroptera	Myotis mystacinus	Weissenbacher-Lang et al. 2023	Detection of Pneumocystis and morphological description of fungal distribution and severity of infection in thirty-six mammal species
Chiroptera	Nyctalus leisleri	Akbar et al. 2012	Characterizing Pneumocystis in the lungs of bats: understanding Pneumocystis evolution and the spread of Pneumocystis organisms in mammal populations
Chiroptera	Nyctalus noctula	Weissenbacher-Lang et al. 2023	Detection of Pneumocystis and morphological description of fungal distribution and severity of infection in thirty-six mammal species
Chiroptera	Vespertilio spp.	Weissenbacher-Lang et al. 2023	Detection of Pneumocystis and morphological description of fungal distribution and severity of infection in thirty-six mammal species
Eulipotyphla	Atelerix albiventris	Weissenbacher-Lang et al. 2023	Detection of Pneumocystis and morphological description of fungal distribution and severity of infection in thirty-six mammal species
Eulipotyphla	Erinaceus europaeus	Settnes et al. 1986	Pneumocystis carinii Delanoe & Delanoe, 1912 found in lungs of freeliving animals in Denmark at Autopsy
Eulipotyphla	Erinaceus europaeus	Weissenbacher-Lang et al. 2023	Detection of Pneumocystis and morphological description of fungal distribution and severity of infection in thirty-six mammal species
Eulipotyphla	Erinaceus roumanicus	Weissenbacher-Lang et al. 2023	Detection of Pneumocystis and morphological description of fungal distribution and severity of infection in thirty-six mammal species
Eulipotyphla	Blarina brevicauda	Laakkonen et al. 1997	Lung parasites of shrews from Pennsylvania
Eulipotyphla	Crocidura leucodon	Weissenbacher-Lang et al. 2023	Detection of Pneumocystis and morphological description of fungal distribution and severity of infection in thirty-six mammal species
Eulipotyphla	Crocidura suaveolens	Sebek and Rosicky 1967	The finding of Pneumocystis carinii in shrews (Incestivora:Soricidae)
Eulipotyphla	Crocidura suaveolens	Weissenbacher-Lang et al. 2023	Detection of Pneumocystis and morphological description of fungal distribution and severity of infection in thirty-six mammal species
Eulipotyphla	Sorex araneus	Settnes and Lodal 1980	Prevalence of Pneumocystis carinii Delanoë & Delanoë, 1912 in rodents in Denmark

Eulipotyphla	Sorex cinereus	Laakkonen et al. 1997	Lung parasites of shrews from Pennsylvania
Eulipotyphla	Sorex fumeus	Laakkonen et al. 1997	Lung parasites of shrews from Pennsylvania
Eulipotyphla	Sorex minutus	Weissenbacher-Lang et al. 2023	Detection of Pneumocystis and morphological description of fungal distribution and severity of infection in thirty-six mammal species
Eulipotyphla	Sorex minutus	Sebek and Rosicky 1967	The finding of Pneumocystis carinii in shrews (Incestivora:Soricidae)
Eulipotyphla	Sorex minutus	Laakkonen 1995	High prevalence of Pneumocystis carinii in Sorex araneus in Finland
Eulipotyphla	Talpa europaea	Weissenbacher-Lang et al. 2023	Detection of Pneumocystis and morphological description of fungal distribution and severity of infection in thirty-six mammal species
Rodentia	Castor fiber	Weissenbacher-Lang et al. 2023	Detection of Pneumocystis and morphological description of fungal distribution and severity of infection in thirty-six mammal species
Rodentia	Arvicola terrestris	Settnes and Lodal 1980	Prevalence of Pneumocystis carinii Delanoë & Delanoë, 1912 in rodents in Denmark
Rodentia	Microtus agrestis	Settnes and Lodal 1980	Prevalence of Pneumocystis carinii Delanoë & Delanoë, 1912 in rodents in Denmark
Rodentia	Microtus agrestis	Sebek and Rosicky 1967	The finding of Pneumocystis carinii in shrews (Incestivora:Soricidae)
Rodentia	Microtus arvalis	Sebek and Rosicky 1967	The finding of Pneumocystis carinii in shrews (Incestivora:Soricidae)
Rodentia	Microtus multiplex	Danesi et al. 2016	Barcoding markers for Pneumocystis species in wildlife
Rodentia	Microtus subterraneus	Sebek and Rosicky 1967	The finding of Pneumocystis carinii in shrews (Incestivora:Soricidae)
Rodentia	Myodes glareolus	Sebek and Rosicky 1967	The finding of Pneumocystis carinii in shrews (Incestivora:Soricidae)
Rodentia	Myodes glareolus	Settnes and Lodal 1980	Prevalence of Pneumocystis carinii Delanoë & Delanoë, 1912 in rodents in Denmark
Rodentia	Myodes rufocanus	Shiota et al. 1986	Prevalence of Pneumocystis carinii in wild rodents in Japan
Rodentia	Myodes smithi	Shiota et al. 1986	Prevalence of Pneumocystis carinii in wild rodents in Japan
Rodentia	Peromyscus boylii	Laakkonen et al. 2001	Pneumocystis in wild small mammals from California
Rodentia	Peromyscus californicus	Laakkonen et al. 2001	Pneumocystis in wild small mammals from California
Rodentia	Peromyscus maniculatus	Laakkonen et al. 2001	Pneumocystis in wild small mammals from California
Rodentia	Reithrodontomys megalotis	Laakkonen et al. 2001	Pneumocystis in wild small mammals from California
Rodentia	Muscardinus avellanarius	Sebek and Rosicky 1967	The finding of Pneumocystis carinii in shrews (Incestivora:Soricidae)
Rodentia	Apodemus agrarius	Laakkonen et al. 2006	Is there an association of Pneumocystis infection with the presence of arena-, hanta-, and poxvirus antibodies in wild mice and shrews in Finland?

Rodentia	Apodemus flavicollis	Settnes and Lodal 1980	Prevalence of Pneumocystis carinii Delanoë & Delanoë, 1912 in rodents in Denmark
Rodentia	Apodemus flavicollis	Sebek and Rosicky 1967	The finding of Pneumocystis carinii in shrews (Incestivora:Soricidae)
Rodentia	Apodemus sylvaticus	Sebek and Rosicky 1967	The finding of Pneumocystis carinii in shrews (Incestivora:Soricidae)
Rodentia	Micromys minutus	Shiota et al. 1986	Prevalence of Pneumocystis carinii in wild rodents in Japan
Rodentia	Micromys minutus	Shiota et al. 1986	Prevalence of Pneumocystis carinii in wild rodents in Japan
Rodentia	Mus musculus	Sebek and Rosicky 1967	The finding of Pneumocystis carinii in shrews (Incestivora:Soricidae)
Rodentia	Rattus everetti	Latinne et al. 2021	Revisiting the Pneumocystis host specificity paradigm and transmission ecology in wild Southeast Asian rodents
Rodentia	Rattus rattus	Settnes and Lodal 1980	Prevalence of Pneumocystis carinii Delanoë & Delanoë, 1912 in rodents in Denmark
Rodentia	Rattus spp.	Shimizu et al. 1985	Occurrence of Pneumocystis carinii in animals in Japan
Rodentia	Myocastor coypus	Weissenbacher-Lang et al. 2023	Detection of Pneumocystis and morphological description of fungal distribution and severity of infection in thirty-six mammal species
Rodentia	Sciurus vulgaris	Weissenbacher-Lang et al. 2023	Detection of Pneumocystis and morphological description of fungal distribution and severity of infection in thirty-six mammal species

Level of infection not indicated (n = 184)

Afrosoricida	Microgale spp.	Laakkonen 1998	Pneumocystis carinii in wildlife
Artiodactyla	Capreolus capreolus	Settnes et al. 1986	Pneumocystis carinii Delanoe & Delanoe, 1912 found in lungs of freeliving animals in Denmark at Autopsy
Carnivora	Canis aureus	Kureljušic et al. 2022	First Molecular Detection of Pneumocystis spp. in the Golden Jackal (Canis aureus)
Carnivora	Nyctereutes procyonoides	Riebold et al. 2020	First molecular detection of Pneumocystis spp. in red foxes (Vulpes vulpes LINNAEUS, 1758) and raccoon dogs (Nyctereutes procyonoides GRAY, 1834)
Carnivora	Vulpes vulpes	Settnes et al. 1986	Pneumocystis carinii Delanoe & Delanoe, 1912 found in lungs of freeliving animals in Denmark at Autopsy
Carnivora	Vulpes vulpes	Riebold et al. 2020	First molecular detection of Pneumocystis spp. in red foxes (Vulpes vulpes LINNAEUS, 1758) and raccoon dogs (Nyctereutes procyonoides GRAY, 1834)

Carnivora	Galictis vittata	Guillot et al. 2001	Parallel phylogenies of Pneumocystis species and their mammalian hosts
Chiroptera	Eumops glaucinus	Veloso et al. 2014	Pneumocystis spp. and Histoplasma capsulatum in bats lungs in Southern and Midwestern Regions of Brazil
Chiroptera	Molossus currentium	Veloso et al. 2014	Pneumocystis spp. and Histoplasma capsulatum in bats lungs in Southern and Midwestern Regions of Brazil
Chiroptera	Molossus molossus	Veloso et al. 2014	Pneumocystis spp. and Histoplasma capsulatum in bats lungs in Southern and Midwestern Regions of Brazil
Chiroptera	Molossus molossus	Sanches et al. 2009	Detection of Pneumocystis in lungs of bats from Brazil by PCR amplification
Chiroptera	Molossus rufus	Veloso et al. 2014	Pneumocystis spp. and Histoplasma capsulatum in bats lungs in Southern and Midwestern Regions of Brazil
Chiroptera	Nyctinomops laticaudatus	Sanches et al. 2009	Detection of Pneumocystis in lungs of bats from Brazil by PCR amplification
Chiroptera	Nyctinomops laticaudatus	Veloso et al. 2014	Pneumocystis spp. and Histoplasma capsulatum in bats lungs in Southern and Midwestern Regions of Brazil
Chiroptera	Nyctinomops macrotis	Veloso et al. 2014	Pneumocystis spp. and Histoplasma capsulatum in bats lungs in Southern and Midwestern Regions of Brazil
Chiroptera	Promops nasutus	Veloso et al. 2014	Pneumocystis spp. and Histoplasma capsulatum in bats lungs in Southern and Midwestern Regions of Brazil
Chiroptera	Tadarida brasiliensis	Sanches et al. 2009	Detection of Pneumocystis in lungs of bats from Brazil by PCR amplification
Chiroptera	Tadarida brasiliensis	Akbar et al. 2012	Characterizing Pneumocystis in the lungs of bats: understanding Pneumocystis evolution and the spread of Pneumocystis organisms in mammal populations
Chiroptera	Tadarida brasiliensis	Derouiche et al. 2009	Pneumocystis diversity as a phyleographic tool
Chiroptera	Tadarida brasiliensis	González-González et al. 2014	Histoplasma capsulatum and Pneumocystis spp. co-infection in wild bats from Argentina, French Guyana, and Mexico
Chiroptera	Tadarida brasiliensis	Veloso et al. 2014	Pneumocystis spp. and Histoplasma capsulatum in bats lungs in Southern and Midwestern Regions of Brazil

Chiroptera	Pteronotus parnellii	Akbar et al. 2012	Characterizing Pneumocystis in the lungs of bats: understanding Pneumocystis evolution and the spread of Pneumocystis organisms in mammal populations
Chiroptera	Natalus stramineus	Akbar et al. 2012	Characterizing Pneumocystis in the lungs of bats: understanding Pneumocystis evolution and the spread of Pneumocystis organisms in mammal populations
Chiroptera	Natalus stramineus	González-González et al. 2014	Histoplasma capsulatum and Pneumocystis spp. co-infection in wild bats from Argentina, French Guyana, and Mexico
Chiroptera	Pteropus rodricensis	Akbar et al. 2012	Characterizing Pneumocystis in the lungs of bats: understanding Pneumocystis evolution and the spread of Pneumocystis organisms in mammal populations
Chiroptera	Rousettus aegyptiacus	Akbar et al. 2012	Characterizing Pneumocystis in the lungs of bats: understanding Pneumocystis evolution and the spread of Pneumocystis organisms in mammal populations
Chiroptera	Artibeus fimbriatus	Sanches et al. 2009	Detection of Pneumocystis in lungs of bats from Brazil by PCR amplification
Chiroptera	Artibeus fimbriatus	Veloso et al. 2014	Pneumocystis spp. and Histoplasma capsulatum in bats lungs in Southern and Midwestern Regions of Brazil
Chiroptera	Carollia perspicillata	Veloso et al. 2014	Pneumocystis spp. and Histoplasma capsulatum in bats lungs in Southern and Midwestern Regions of Brazil
Chiroptera	Desmodus rotundus	Sanches et al. 2009	Detection of Pneumocystis in lungs of bats from Brazil by PCR amplification
Chiroptera	Desmodus rotundus	Veloso et al. 2014	Pneumocystis spp. and Histoplasma capsulatum in bats lungs in Southern and Midwestern Regions of Brazil
Chiroptera	Diphylla ecaudata	Sanches et al. 2009	Detection of Pneumocystis in lungs of bats from Brazil by PCR amplification
Chiroptera	Glossophaga soricina	Guillot et al. 2001	Parallel phylogenies of Pneumocystis species and their mammalian hosts
Chiroptera	Glossophaga soricina	Akbar et al. 2012	Characterizing Pneumocystis in the lungs of bats: understanding Pneumocystis evolution and the spread of Pneumocystis organisms in mammal populations
Chiroptera	Glossophaga soricina	Derouiche et al. 2009	Pneumocystis diversity as a phyleographic tool

Chiroptera	Glossophaga soricina	González-González et al. 2014	Histoplasma capsulatum and Pneumocystis spp. co-infection in wild bats from Argentina, French Guyana, and Mexico
Chiroptera	Glossophaga soricina	Veloso et al. 2014	Pneumocystis spp. and Histoplasma capsulatum in bats lungs in Southern and Midwestern Regions of Brazil
Chiroptera	Rhinophylla pumilio	Veloso et al. 2014	Pneumocystis spp. and Histoplasma capsulatum in bats lungs in Southern and Midwestern Regions of Brazil
Chiroptera	Sturnira lilium	Sanches et al. 2009	Detection of Pneumocystis in lungs of bats from Brazil by PCR amplification
Chiroptera	Aeorestes cinereus	Veloso et al. 2014	Pneumocystis spp. and Histoplasma capsulatum in bats lungs in Southern and Midwestern Regions of Brazil
Chiroptera	Eptesicus furinalis	Veloso et al. 2014	Pneumocystis spp. and Histoplasma capsulatum in bats lungs in Southern and Midwestern Regions of Brazil
Chiroptera	Eptesicus serotinus	Akbar et al. 2012	Characterizing Pneumocystis in the lungs of bats: understanding Pneumocystis evolution and the spread of Pneumocystis organisms in mammal populations
Chiroptera	Histiotus velatus	Veloso et al. 2014	Pneumocystis spp. and Histoplasma capsulatum in bats lungs in Southern and Midwestern Regions of Brazil
Chiroptera	Lasiurus blossevillii	Veloso et al. 2014	Pneumocystis spp. and Histoplasma capsulatum in bats lungs in Southern and Midwestern Regions of Brazil
Chiroptera	Myotis californicus	Akbar et al. 2012	Characterizing Pneumocystis in the lungs of bats: understanding Pneumocystis evolution and the spread of Pneumocystis organisms in mammal populations
Chiroptera	Myotis californicus	González-González et al. 2014	Histoplasma capsulatum and Pneumocystis spp. co-infection in wild bats from Argentina, French Guyana, and Mexico
Chiroptera	Myotis levis	Sanches et al. 2009	Detection of Pneumocystis in lungs of bats from Brazil by PCR amplification
Chiroptera	Myotis levis	Veloso et al. 2014	Pneumocystis spp. and Histoplasma capsulatum in bats lungs in Southern and Midwestern Regions of Brazil
Chiroptera	Myotis nigricans	Veloso et al. 2014	Pneumocystis spp. and Histoplasma capsulatum in bats lungs in Southern and Midwestern Regions of Brazil
Chiroptera	Nyctalus noctula	Akbar et al. 2012	Characterizing Pneumocystis in the lungs of bats: understanding Pneumocystis evolution and the spread of Pneumocystis organisms in mammal populations

Chiroptera	Pipistrellus pipistrellus	Akbar et al. 2012	Characterizing Pneumocystis in the lungs of bats: understanding Pneumocystis evolution and the spread of Pneumocystis organisms in mammal populations
Chiroptera	Pipistrellus pipistrellus	Derouiche et al. 2009	Pneumocystis diversity as a phyleographic tool
Chiroptera	Plecotus auritus	Akbar et al. 2012	Characterizing Pneumocystis in the lungs of bats: understanding Pneumocystis evolution and the spread of Pneumocystis organisms in mammal populations
Chiroptera	Plecotus austriacus	Akbar et al. 2012	Characterizing Pneumocystis in the lungs of bats: understanding Pneumocystis evolution and the spread of Pneumocystis organisms in mammal populations
Didelphimorphia	Marmosa murina	Guillot et al. 2001	Parallel phylogenies of Pneumocystis species and their mammalian hosts
Eulipotyphla	Neomys fodiens	Kučera et al. 1971	Pneumocystose
Eulipotyphla	Sorex antinorii	Danesi et al. 2016	Barcoding markers for Pneumocystis species in wildlife
Eulipotyphla	Sorex araneus	Kučera et al. 1971	Pneumocystose
Eulipotyphla	Sorex araneus	Laakkonen 1998	Pneumocystis carinii in wildlife
Eulipotyphla	Sorex araneus	Laakkonen 1998	Pneumocystis carinii in wildlife
Eulipotyphla	Sorex araneus	Laakkonen et al. 1993	Pneumocystis carinii and helminth parasitism in shrews Sorex araneus and Sorex caecutiens
Eulipotyphla	Sorex caecutiens	Laakkonen et al. 1993	Pneumocystis carinii and helminth parasitism in shrews Sorex araneus and Sorex caecutiens
Eulipotyphla	Sorex isodon	Laakkonen 1998	Pneumocystis carinii in wildlife
Eulipotyphla	Sorex minutissimus	Laakkonen 1998	Pneumocystis carinii in wildlife
Eulipotyphla	Sorex minutus	Kučera et al. 1971	Pneumocystose
Eulipotyphla	Sorex minutus	Laakkonen 1998	Pneumocystis carinii in wildlife
Eulipotyphla	Sorex minutus	Kučera et al. 1971	Pneumocystose
Eulipotyphla	Sorex spp.	Peters et al. 1994	DNA analysis of Pneumocystis carinii infecting Finnish and English shrews
Eulipotyphla	Sorex spp.	Peters et al. 1994	DNA analysis of Pneumocystis carinii infecting Finnish and English shrews
Lagomorpha	Lepus europaeus	Settnes et al. 1986	Pneumocystis carinii Delanoe & Delanoe, 1912 found in lungs of freeliving animals in Denmark at Autopsy

Primates	Pithecia pithecia	Demanche et al. 2001	Phylogeny of Pneumocystis carinii from 18 primate species confirms host specificity and suggests coevolution
Rodentia	Heliophobius argenteocinereus	Petružela et al. 2019	Evolutionary history of Pneumocystis fungi in their African rodent hosts
Rodentia	Microtus agrestis	Kučera et al. 1971	Pneumocystose
Rodentia	Microtus agrestis	Kučera et al. 1971	Pneumocystose
Rodentia	Microtus agrestis	Kučera et al. 1971	Pneumocystose
Rodentia	Microtus arvalis	Kučera et al. 1971	Pneumocystose
Rodentia	Microtus arvalis	Kučera et al. 1971	Pneumocystose
Rodentia	Microtus subterraneus	Kučera et al. 1971	Pneumocystose
Rodentia	Microtus subterraneus	Kučera et al. 1971	Pneumocystose
Rodentia	Myodes glareolus	Kučera et al. 1971	Pneumocystose
Rodentia	Myodes glareolus	Kučera et al. 1971	Pneumocystose
Rodentia	Myodes glareolus	Kučera et al. 1971	Pneumocystose
Rodentia	Myodes glareolus	Danesi et al. 2016	Barcoding markers for Pneumocystis species in wildlife
Rodentia	Peromyscus spp.	Salazar-Hamm et al. 2022	Breathing can be dangerous: Opportunistic fungal pathogens and the diverse community of the small mammal lung microbiome
Rodentia	Agouti paca	Guillot et al. 2001	Parallel phylogenies of Pneumocystis species and their mammalian hosts
Rodentia	Proechimys guyannensis	Guillot et al. 2001	Parallel phylogenies of Pneumocystis species and their mammalian hosts
Rodentia	Graphiurus murinus	Petružela et al. 2019	Evolutionary history of Pneumocystis fungi in their African rodent hosts
Rodentia	Graphiuru ssp.	Petružela et al. 2019	Evolutionary history of Pneumocystis fungi in their African rodent hosts
Rodentia	Dipodomys spp.	Salazar-Hamm et al. 2022	Breathing can be dangerous: Opportunistic fungal pathogens and the diverse community of the small mammal lung microbiome
Rodentia	Acomys ignitus	Petružela et al. 2019	Evolutionary history of Pneumocystis fungi in their African rodent hosts
Rodentia	Acomys muzei	Petružela et al. 2019	Evolutionary history of Pneumocystis fungi in their African rodent hosts

Rodentia	Acomys ngurui	Petružela et al. 2019	Evolutionary history of Pneumocystis fungi in their African rodent hosts
Rodentia	Acomys wilsoni	Petružela et al. 2019	Evolutionary history of Pneumocystis fungi in their African rodent hosts
Rodentia	Aethomys chrysophilus	Petružela et al. 2019	Evolutionary history of Pneumocystis fungi in their African rodent hosts
Rodentia	Aethomys hindei	Petružela et al. 2019	Evolutionary history of Pneumocystis fungi in their African rodent hosts
Rodentia	Aethomys kaiseri	Petružela et al. 2019	Evolutionary history of Pneumocystis fungi in their African rodent hosts
Rodentia	Apodemus flavicollis	Danesi et al. 2016	Barcoding markers for Pneumocystis species in wildlife
Rodentia	Apodemus flavicollis	Kučera et al. 1971	Pneumocystose
Rodentia	Apodemus sylvaticus	Kučera et al. 1971	Pneumocystose
Rodentia	Apodemus sylvaticus	Settnes and Lodal 1980	Prevalence of Pneumocystis carinii Delanoë & Delanoë, 1912 in rodents in Denmark
Rodentia	Apodemus sylvaticus	Demanche et al. 2015	What Do Pneumocystis Organisms Tell Us about the Phylogeography of Their Hosts? The Case of the Woodmouse Apodemus sylvaticus in Continental Europe and Western Mediterranean Islands
Rodentia	Apodemus spp.	Kučera et al. 1971	Pneumocystose
Rodentia	Apomys banahao	Latinne et al. 2021	Revisiting the Pneumocystis host specificity paradigm and transmission ecology in wild Southeast Asian rodents
Rodentia	Bandicota indica	Latinne et al. 2021	Revisiting the Pneumocystis host specificity paradigm and transmission ecology in wild Southeast Asian rodents
Rodentia	Bandicota indica	Latinne et al. 2018	Genetic diversity and evolution of Pneumocystis fungi infecting wild Southeast Asian murid rodents
Rodentia	Bandicota savilei	Latinne et al. 2021	Revisiting the Pneumocystis host specificity paradigm and transmission ecology in wild Southeast Asian rodents
Rodentia	Bandicota savilei	Latinne et al. 2018	Genetic diversity and evolution of Pneumocystis fungi infecting wild Southeast Asian murid rodents
Rodentia	Berylmys berdmorei	Latinne et al. 2021	Revisiting the Pneumocystis host specificity paradigm and transmission ecology in wild Southeast Asian rodents

Rodentia	Berylmys berdmorei	Latinne et al. 2018	Genetic diversity and evolution of Pneumocystis fungi infecting wild Southeast Asian murid rodents
Rodentia	Berylmys bowersi	Latinne et al. 2021	Revisiting the Pneumocystis host specificity paradigm and transmission ecology in wild Southeast Asian rodents
Rodentia	Berylmys bowersi	Latinne et al. 2018	Genetic diversity and evolution of Pneumocystis fungi infecting wild Southeast Asian murid rodents
Rodentia	Gerbilliscus vicinus	Petružela et al. 2019	Evolutionary history of Pneumocystis fungi in their African rodent hosts
Rodentia	Grammomys surdaster	Petružela et al. 2019	Evolutionary history of Pneumocystis fungi in their African rodent hosts
Rodentia	Hylomyscus arcimontensis	Petružela et al. 2019	Evolutionary history of Pneumocystis fungi in their African rodent hosts
Rodentia	Lemniscomys rosalia	Petružela et al. 2019	Evolutionary history of Pneumocystis fungi in their African rodent hosts
Rodentia	Lemniscomys striatus	Petružela et al. 2019	Evolutionary history of Pneumocystis fungi in their African rodent hosts
Rodentia	Leopoldamys herberti	Latinne et al. 2021	Revisiting the Pneumocystis host specificity paradigm and transmission ecology in wild Southeast Asian rodents
Rodentia	Leopoldamys herberti	Latinne et al. 2018	Genetic diversity and evolution of Pneumocystis fungi infecting wild Southeast Asian murid rodents
Rodentia	Leopoldamys neilli	Latinne et al. 2021	Revisiting the Pneumocystis host specificity paradigm and transmission ecology in wild Southeast Asian rodents
Rodentia	Leopoldamys sabanus	Latinne et al. 2021	Revisiting the Pneumocystis host specificity paradigm and transmission ecology in wild Southeast Asian rodents
Rodentia	Lophuromys kilonzoi	Petružela et al. 2019	Evolutionary history of Pneumocystis fungi in their African rodent hosts
Rodentia	Lophuromys makundii	Petružela et al. 2019	Evolutionary history of Pneumocystis fungi in their African rodent hosts
Rodentia	Mastomys natalensis	Petružela et al. 2019	Evolutionary history of Pneumocystis fungi in their African rodent hosts
Rodentia	Maxomys surifer	Latinne et al. 2021	Revisiting the Pneumocystis host specificity paradigm and transmission ecology in wild Southeast Asian rodents

Rodentia	Maxomys surifer	Latinne et al. 2018	Genetic diversity and evolution of Pneumocystis fungi infecting wild Southeast Asian murid rodents
Rodentia	Mus caroli	Latinne et al. 2018	Genetic diversity and evolution of Pneumocystis fungi infecting wild Southeast Asian murid rodents
Rodentia	Mus caroli	Latinne et al. 2021	Revisiting the Pneumocystis host specificity paradigm and transmission ecology in wild Southeast Asian rodents
Rodentia	Mus cervicolor	Latinne et al. 2021	Revisiting the Pneumocystis host specificity paradigm and transmission ecology in wild Southeast Asian rodents
Rodentia	Mus cervicolor	Latinne et al. 2018	Genetic diversity and evolution of Pneumocystis fungi infecting wild Southeast Asian murid rodents
Rodentia	Mus cookii	Latinne et al. 2018	Genetic diversity and evolution of Pneumocystis fungi infecting wild Southeast Asian murid rodents
Rodentia	Mus cookii	Latinne et al. 2021	Revisiting the Pneumocystis host specificity paradigm and transmission ecology in wild Southeast Asian rodents
Rodentia	Mus minutoides	Petružela et al. 2019	Evolutionary history of Pneumocystis fungi in their African rodent hosts
Rodentia	Mus musculus	Shimizu et al. 1985	Occurrence of Pneumocystis carinii in animals in Japan
Rodentia	Mus musculus	Kučera et al. 1971	Pneumocystose
Rodentia	Mus musculus	Settnes and Lodal 1980	Prevalence of Pneumocystis carinii Delanoë & Delanoë, 1912 in rodents in Denmark
Rodentia	Mus musculus	Porter 1915	The occurrence of Pneumocystis carinii in mice in England
Rodentia	Mus musculus	Bellocq et al. 2018	Holobiont suture zones: Parasite evidence across the European house mouse hybrid zone
Rodentia	Mus pahari	Latinne et al. 2021	Revisiting the Pneumocystis host specificity paradigm and transmission ecology in wild Southeast Asian rodents
Rodentia	Mus triton	Petružela et al. 2019	Evolutionary history of Pneumocystis fungi in their African rodent hosts
Rodentia	Myomyscus brockmani	Petružela et al. 2019	Evolutionary history of Pneumocystis fungi in their African rodent hosts
Rodentia	Niviventer fulvescens	Latinne et al. 2021	Revisiting the Pneumocystis host specificity paradigm and transmission ecology in wild Southeast Asian rodents

Rodentia	Niviventer fulvescens	Latinne et al. 2018	Genetic diversity and evolution of Pneumocystis fungi infecting wild Southeast Asian murid rodents
Rodentia	Otomys angoniensis	Petružela et al. 2019	Evolutionary history of Pneumocystis fungi in their African rodent hosts
Rodentia	Praomys delectorum	Petružela et al. 2019	Evolutionary history of Pneumocystis fungi in their African rodent hosts
Rodentia	Rattus andamanensis	Latinne et al. 2021	Revisiting the Pneumocystis host specificity paradigm and transmission ecology in wild Southeast Asian rodents
Rodentia	Rattus andamanensis	Latinne et al. 2018	Genetic diversity and evolution of Pneumocystis fungi infecting wild Southeast Asian murid rodents
Rodentia	Rattus argentiventer	Latinne et al. 2021	Revisiting the Pneumocystis host specificity paradigm and transmission ecology in wild Southeast Asian rodents
Rodentia	Rattus argentiventer	Latinne et al. 2018	Genetic diversity and evolution of Pneumocystis fungi infecting wild Southeast Asian murid rodents
Rodentia	Rattus exulans	Latinne et al. 2021	Revisiting the Pneumocystis host specificity paradigm and transmission ecology in wild Southeast Asian rodents
Rodentia	Rattus exulans	Latinne et al. 2018	Genetic diversity and evolution of Pneumocystis fungi infecting wild Southeast Asian murid rodents
Rodentia	Rattus nitidus	Latinne et al. 2021	Revisiting the Pneumocystis host specificity paradigm and transmission ecology in wild Southeast Asian rodents
Rodentia	Rattus nitidus	Latinne et al. 2018	Genetic diversity and evolution of Pneumocystis fungi infecting wild Southeast Asian murid rodents
Rodentia	Rattus norvegicus	Shimizu et al. 1985	Occurrence of Pneumocystis carinii in animals in Japan
Rodentia	Rattus norvegicus	Kučera et al. 1971	Pneumocystose
Rodentia	Rattus norvegicus	Settnes and Lodal 1980	Prevalence of Pneumocystis carinii Delanoë & Delanoë, 1912 in rodents in Denmark
Rodentia	Rattus norvegicus	Yoshida and Ikai 1979	Pneumocystis carinii pneumonia: epidemiology in Japan and cyst concentration method
Rodentia	Rattus norvegicus	Palmer et al. 2000	Population structure of rat-derived Pneumocystis carinii in Danish wild rats
Rodentia	Rattus norvegicus	Chabé et al. 2010	Pneumocystis carinii and Pneumocystis wakefieldiae in wild Rattus norvegicus trapped in Thailand

Rodentia	Rattus norvegicus	Latinne et al. 2021	Revisiting the Pneumocystis host specificity paradigm and transmission ecology in wild Southeast Asian rodents
Rodentia	Rattus norvegicus	Latinne et al. 2018	Genetic diversity and evolution of Pneumocystis fungi infecting wild Southeast Asian murid rodents
Rodentia	Rattus norvegicus	Yoshida and Ikai 1979	Pneumocystis carinii pneumonia: epidemiology in Japan and cyst concentration method
Rodentia	Rattus rattus	Shimizu et al. 1985	Occurrence of Pneumocystis carinii in animals in Japan
Rodentia	Rattus rattus	Shimizu et al. 1985	Occurrence of Pneumocystis carinii in animals in Japan
Rodentia	Rattus sakeratensis	Latinne et al. 2021	Revisiting the Pneumocystis host specificity paradigm and transmission ecology in wild Southeast Asian rodents
Rodentia	Rattus sakeratensis	Latinne et al. 2018	Genetic diversity and evolution of Pneumocystis fungi infecting wild Southeast Asian murid rodents
Rodentia	Rattus tanezumi R2	Latinne et al. 2021	Revisiting the Pneumocystis host specificity paradigm and transmission ecology in wild Southeast Asian rodents
Rodentia	Rattus tanezumi R2	Latinne et al. 2018	Genetic diversity and evolution of Pneumocystis fungi infecting wild Southeast Asian murid rodents
Rodentia	Rattus tanezumi R3	Latinne et al. 2021	Revisiting the Pneumocystis host specificity paradigm and transmission ecology in wild Southeast Asian rodents
Rodentia	Rattus tanezumi R3	Latinne et al. 2018	Genetic diversity and evolution of Pneumocystis fungi infecting wild Southeast Asian murid rodents
Rodentia	Rattus tiomanicus	Latinne et al. 2021	Revisiting the Pneumocystis host specificity paradigm and transmission ecology in wild Southeast Asian rodents
Rodentia	Rattus rattus/Rattus norvegicus	Rothenburger et al. 2015	Respiratory Pathology and Pathogens in Wild Urban Rats (Rattus norvegicus and Rattus rattus)
Rodentia	Rattus rattus/Rattus norvegicus	Boey et al. 2019	Seroprevalence of Rodent Pathogens in Wild Rats from the Island of St. Kitts, West Indies
Rodentia	Rhabdomys dilectus	Petružela et al. 2019	Evolutionary history of Pneumocystis fungi in their African rodent hosts
Rodentia	Sundamys muelleri	Latinne et al. 2021	Revisiting the Pneumocystis host specificity paradigm and transmission ecology in wild Southeast Asian rodents
Rodentia	Myocastor coypus	Danesi et al. 2016	Barcoding markers for Pneumocystis species in wildlife

Rodentia	Myocastor coypus	Danesi et al. 2018	Real-time PCR assay for screening Pneumocystis in free-living wild squirrels and river rats
Rodentia	Saccostomus umbriventer	Petružela et al. 2019	Evolutionary history of Pneumocystis fungi in their African rodent hosts
Rodentia	Steatomys spp.	Petružela et al. 2019	Evolutionary history of Pneumocystis fungi in their African rodent hosts
Rodentia	Callosciurus finlaysonii	Danesi et al. 2018	Real-time PCR assay for screening Pneumocystis in free-living wild squirrels and river rats
Rodentia	Sciurus aestuans	Guillot et al. 2001	Parallel phylogenies of Pneumocystis species and their mammalian hosts
Rodentia	Cannomys badius	Latinne et al. 2021	Revisiting the Pneumocystis host specificity paradigm and transmission ecology in wild Southeast Asian rodents
Rodentia	Cannomys badius	Latinne et al. 2018	Genetic diversity and evolution of Pneumocystis fungi infecting wild Southeast Asian murid rodents
Rodentia	Rhizomys pruinosus	Latinne et al. 2021	Revisiting the Pneumocystis host specificity paradigm and transmission ecology in wild Southeast Asian rodents
Rodentia	Tachyoryctes splendens	Petružela et al. 2019	Evolutionary history of Pneumocystis fungi in their African rodent hosts

Species not exactly defined (n = 2)

Chiroptera	Artibeus spp.	Cavallini Sanches et al. 2013	Pneumocystis spp. in bats evaluated by qPCR
Rodentia	Rattus spp.	Meer and Brug 1942	Infection a Pneumocystis chez l'homme et chez les animaux

5. Prevalence x level of infection.

Only negative results (n = 23)

Artiodactyla	Bos taurus	Yoshida and Ikai 1979	Pneumocystis carinii pneumonia: epidemiology in Japan and cyst concentration method

Artiodactyla	Sus scrofa domesticus	Yoshida and Ikai 1979	Pneumocystis carinii pneumonia: epidemiology in Japan and cyst concentration method
Artiodactyla	Sus scrofa domesticus	Davalos 1963	Latent infection produced by Pneumocystis carinii in domestic animals in Mexico
Carnivora	Canis lupus familiaris	Wöhrer et al. 2016	Age-related presence of selected viral and bacterial pathogens in paraffin-embedded lung samples of dogs with pneumonia
Carnivora	Vulpes vulpes	Danesi et al. 2016	Barcoding markers for Pneumocystis species in wildlife
Carnivora	Felis catus	Hagler et al. 1987	Feline leukemia virus and Pneumocystis carinii infection
Carnivora	Meles meles	Danesi et al. 2016	Barcoding markers for Pneumocystis species in wildlife
Carnivora	Mustela sibirica	Yoshida and Ikai 1979	Pneumocystis carinii pneumonia: epidemiology in Japan and cyst concentration method
Eulipotyphla	Erinaceus europaeus	Weissenbacher-Lang et al. 2023	Detection of Pneumocystis and morphological description of fungal distribution and severity of infection in thirty-six mammal species
Eulipotyphla	Blarina brevicauda	Laakkonen et al. 1997	Lung parasites of shrews from Pennsylvania
Eulipotyphla	Sorex cinereus	Laakkonen et al. 1997	Lung parasites of shrews from Pennsylvania
Eulipotyphla	Sorex fumeus	Laakkonen et al. 1997	Lung parasites of shrews from Pennsylvania
Eulipotyphla	Sorex minutus	Laakkonen 1995	High prevalence of Pneumocystis carinii in Sorex araneus in Finland
Primates	Callithrix jacchus	Weissenbacher-Lang et al. 2023	Detection of Pneumocystis and morphological description of fungal distribution and severity of infection in thirty-six mammal species
Rodentia	Microtus agrestis	Sebek and Rosicky 1967	The finding of Pneumocystis carinii in shrews (Incestivora:Soricidae)
Rodentia	Microtus subterraneus	Sebek and Rosicky 1967	The finding of Pneumocystis carinii in shrews (Incestivora:Soricidae)
Rodentia	Myodes glareolus	Sebek and Rosicky 1967	The finding of Pneumocystis carinii in shrews (Incestivora:Soricidae)
Rodentia	Myodes smithi	Shiota et al. 1986	Prevalence of Pneumocystis carinii in wild rodents in Japan

Rodentia	Peromyscus californicus	Laakkonen et al. 2001	Pneumocystis in wild small mammals from California
Rodentia	Reithrodontomys megalotis	Laakkonen et al. 2001	Pneumocystis in wild small mammals from California
Rodentia	Apodemus agrarius	Laakkonen et al. 2006	Is there an association of Pneumocystis infection with the presence of arena-, hanta-, and poxvirus antibodies in wild mice and shrews in Finland?
Rodentia	Apodemus flavicollis	Sebek and Rosicky 1967	The finding of Pneumocystis carinii in shrews (Incestivora:Soricidae)
Rodentia	Apodemus sylvaticus	Sebek and Rosicky 1967	The finding of Pneumocystis carinii in shrews (Incestivora:Soricidae)

Level of infection not indicated (n = 104)

Artiodactyla	Bos taurus	Shimizu et al. 1985	Occurrence of Pneumocystis carinii in animals in Japan
Artiodactyla	Bos taurus	Settnes and Henriksen 1989	Pneumocystis carinii in large domestic animals in Denmark
Artiodactyla	Ovis aries	Settnes and Henriksen 1989	Pneumocystis carinii in large domestic animals in Denmark
Artiodactyla	Sus scrofa domesticus	Shimizu et al. 1985	Occurrence of Pneumocystis carinii in animals in Japan
Artiodactyla	Sus scrofa domesticus	Settnes and Henriksen 1989	Pneumocystis carinii in large domestic animals in Denmark
Artiodactyla	Sus scrofa domesticus	Huang et al. 2022	A total infectome approach to understand the etiology of infectious disease in pigs
Artiodactyla	Sus scrofa domesticus	Esgalhado et al. 2013	Study of the epidemiology of Pneumocystis carinii f. sp. suis in abattoir swine in Portugal
Artiodactyla	Sus scrofa domesticus	Sanches et al. 2006	Co-Infection of Pneumocystis carinii f. sp. suis and Porcine Circovirus-2 (PCV2) in Pig Lungs Obtained from Slaughterhouses in Southern and Midwestern Regions of Brazil
Carnivora	Canis aureus	Kureljušic et al. 2022	First Molecular Detection of Pneumocystis spp. in the Golden Jackal (Canis aureus)
Carnivora	Canis lupus familiaris	Yoshida and Ikai 1979	Pneumocystis carinii pneumonia: epidemiology in Japan and cyst concentration method
Carnivora	Canis lupus familiaris	Sukura et al. 1997	Occurrence of Pneumocystis carinii in canine distemper
Carnivora	Canis lupus familiaris	Davalos 1963	Latent infection produced by Pneumocystis carinii in domestic animals in Mexico

Carnivora	Canis lupus familiaris	Zavala and Rosado 1972	Pneumocystis carinii in domestic animals of the city of Mérida, Yucatán
Carnivora	Nyctereutes procyonoides	Riebold et al. 2020	First molecular detection of Pneumocystis spp. in red foxes (Vulpes vulpes LINNAEUS, 1758) and raccoon dogs (Nyctereutes procyonoides GRAY, 1834)
Carnivora	Vulpes vulpes	Settnes et al. 1986	Pneumocystis carinii Delanoe & Delanoe, 1912 found in lungs of freeliving animals in Denmark at Autopsy
Carnivora	Vulpes vulpes	Riebold et al. 2020	First molecular detection of Pneumocystis spp. in red foxes (Vulpes vulpes LINNAEUS, 1758) and raccoon dogs (Nyctereutes procyonoides GRAY, 1834)
Carnivora	Felis catus	Zavala and Rosado 1972	Pneumocystis carinii in domestic animals of the city of Mérida, Yucatán
Carnivora	Felis catus	Danesi et al. 2019	Molecular detection of Pneumocystis in the lungs of cats
Carnivora	Felis catus	Davalos 1963	Latent infection produced by Pneumocystis carinii in domestic animals in Mexico
Chiroptera	Molossus molossus	Veloso et al. 2014	Pneumocystis spp. and Histoplasma capsulatum in bats lungs in Southern and Midwestern Regions of Brazil
Chiroptera	Molossus molossus	Sanches et al. 2009	Detection of Pneumocystis in lungs of bats from Brazil by PCR amplification
Chiroptera	Nyctinomops laticaudatus	Sanches et al. 2009	Detection of Pneumocystis in lungs of bats from Brazil by PCR amplification
Chiroptera	Nyctinomops laticaudatus	Veloso et al. 2014	Pneumocystis spp. and Histoplasma capsulatum in bats lungs in Southern and Midwestern Regions of Brazil
Chiroptera	Tadarida brasiliensis	Sanches et al. 2009	Detection of Pneumocystis in lungs of bats from Brazil by PCR amplification
Chiroptera	Tadarida brasiliensis	Akbar et al. 2012	Characterizing Pneumocystis in the lungs of bats: understanding Pneumocystis evolution and the spread of Pneumocystis organisms in mammal populations
Chiroptera	Tadarida brasiliensis	Derouiche et al. 2009	Pneumocystis diversity as a phyleographic tool
Chiroptera	Tadarida brasiliensis	González-González et al. 2014	Histoplasma capsulatum and Pneumocystis spp. co-infection in wild bats from Argentina, French Guyana, and Mexico

Chiroptera	Tadarida brasiliensis	Veloso et al. 2014	Pneumocystis spp. and Histoplasma capsulatum in bats lungs in Southern and Midwestern Regions of Brazil
Chiroptera	Pteropus rodricensis	Akbar et al. 2012	Characterizing Pneumocystis in the lungs of bats: understanding Pneumocystis evolution and the spread of Pneumocystis organisms in mammal populations
Chiroptera	Rousettus aegyptiacus	Akbar et al. 2012	Characterizing Pneumocystis in the lungs of bats: understanding Pneumocystis evolution and the spread of Pneumocystis organisms in mammal populations
Chiroptera	Desmodus rotundus	Sanches et al. 2009	Detection of Pneumocystis in lungs of bats from Brazil by PCR amplification
Chiroptera	Desmodus rotundus	Veloso et al. 2014	Pneumocystis spp. and Histoplasma capsulatum in bats lungs in Southern and Midwestern Regions of Brazil
Chiroptera	Glossophaga soricina	Akbar et al. 2012	Characterizing Pneumocystis in the lungs of bats: understanding Pneumocystis evolution and the spread of Pneumocystis organisms in mammal populations
Chiroptera	Glossophaga soricina	Derouiche et al. 2009	Pneumocystis diversity as a phyleographic tool
Chiroptera	Glossophaga soricina	González-González et al. 2014	Histoplasma capsulatum and Pneumocystis spp. co-infection in wild bats from Argentina, French Guyana, and Mexico
Chiroptera	Histiotus velatus	Veloso et al. 2014	Pneumocystis spp. and Histoplasma capsulatum in bats lungs in Southern and Midwestern Regions of Brazil
Chiroptera	Nyctalus noctula	Akbar et al. 2012	Characterizing Pneumocystis in the lungs of bats: understanding Pneumocystis evolution and the spread of Pneumocystis organisms in mammal populations
Eulipotyphla	Sorex antinorii	Danesi et al. 2016	Barcoding markers for Pneumocystis species in wildlife
Eulipotyphla	Sorex araneus	Laakkonen 1998	Pneumocystis carinii in wildlife
Eulipotyphla	Sorex araneus	Laakkonen 1998	Pneumocystis carinii in wildlife
Eulipotyphla	Sorex araneus	Laakkonen et al. 1993	Pneumocystis carinii and helminth parasitism in shrews Sorex araneus and Sorex caecutiens
Eulipotyphla	Sorex caecutiens	Laakkonen et al. 1993	Pneumocystis carinii and helminth parasitism in shrews Sorex araneus and Sorex caecutiens
Lagomorpha	Lepus europaeus	Settnes et al. 1986	Pneumocystis carinii Delanoe & Delanoe, 1912 found in lungs of freeliving animals in Denmark at Autopsy

Lagomorpha	Oryctolagus cuniculus	Davalos 1963	Latent infection produced by Pneumocystis carinii in domestic animals in Mexico
Perissodactyla	Equus caballus	Prescott et al. 1991	Sporadic, severe bronchointerstitial pneumonia of foals
Primates	Callithrix jacchus	Demanche et al. 2001	Phylogeny of Pneumocystis carinii from 18 primate species confirms host specificity and suggests coevolution
Primates	Macaca fascicularis	Demanche et al. 2005	Molecular and serological evidence of Pneumocystis circulation in a social organization of healthy macaques (Macaca fascicularis)
Rodentia	Microtus agrestis	Kučera et al. 1971	Pneumocystose
Rodentia	Microtus arvalis	Kučera et al. 1971	Pneumocystose
Rodentia	Myodes glareolus	Kučera et al. 1971	Pneumocystose
Rodentia	Graphiurus murinus	Petružela et al. 2019	Evolutionary history of Pneumocystis fungi in their African rodent hosts
Rodentia	Acomys wilsoni	Petružela et al. 2019	Evolutionary history of Pneumocystis fungi in their African rodent hosts
Rodentia	Aethomys chrysophilus	Petružela et al. 2019	Evolutionary history of Pneumocystis fungi in their African rodent hosts
Rodentia	Apodemus flavicollis	Danesi et al. 2016	Barcoding markers for Pneumocystis species in wildlife
Rodentia	Apodemus sylvaticus	Petružela et al. 2019	Evolutionary history of Pneumocystis fungi in their African rodent hosts
Rodentia	Bandicota indica	Latinne et al. 2021	Revisiting the Pneumocystis host specificity paradigm and transmission ecology in wild Southeast Asian rodents
Rodentia	Bandicota indica	Latinne et al. 2018	Genetic diversity and evolution of Pneumocystis fungi infecting wild Southeast Asian murid rodents
Rodentia	Bandicota savilei	Latinne et al. 2021	Revisiting the Pneumocystis host specificity paradigm and transmission ecology in wild Southeast Asian rodents
Rodentia	Bandicota savilei	Latinne et al. 2018	Genetic diversity and evolution of Pneumocystis fungi infecting wild Southeast Asian murid rodents
Rodentia	Berylmys berdmorei	Latinne et al. 2021	Revisiting the Pneumocystis host specificity paradigm and transmission ecology in wild Southeast Asian rodents
Rodentia	Berylmys berdmorei	Latinne et al. 2018	Genetic diversity and evolution of Pneumocystis fungi infecting wild Southeast Asian murid rodents

Rodentia	Gerbilliscus vicinus	Petružela et al. 2019	Evolutionary history of Pneumocystis fungi in their African rodent hosts
Rodentia	Leopoldamys herberti	Latinne et al. 2021	Revisiting the Pneumocystis host specificity paradigm and transmission ecology in wild Southeast Asian rodents
Rodentia	Mastomys natalensis	Petružela et al. 2019	Evolutionary history of Pneumocystis fungi in their African rodent hosts
Rodentia	Maxomys surifer	Latinne et al. 2021	Revisiting the Pneumocystis host specificity paradigm and transmission ecology in wild Southeast Asian rodents
Rodentia	Maxomys surifer	Latinne et al. 2018	Genetic diversity and evolution of Pneumocystis fungi infecting wild Southeast Asian murid rodents
Rodentia	Mus caroli	Latinne et al. 2018	Genetic diversity and evolution of Pneumocystis fungi infecting wild Southeast Asian murid rodents
Rodentia	Mus caroli	Latinne et al. 2021	Revisiting the Pneumocystis host specificity paradigm and transmission ecology in wild Southeast Asian rodents
Rodentia	Mus cervicolor	Latinne et al. 2021	Revisiting the Pneumocystis host specificity paradigm and transmission ecology in wild Southeast Asian rodents
Rodentia	Mus cervicolor	Latinne et al. 2018	Genetic diversity and evolution of Pneumocystis fungi infecting wild Southeast Asian murid rodents
Rodentia	Mus cookii	Latinne et al. 2018	Genetic diversity and evolution of Pneumocystis fungi infecting wild Southeast Asian murid rodents
Rodentia	Mus cookii	Latinne et al. 2021	Revisiting the Pneumocystis host specificity paradigm and transmission ecology in wild Southeast Asian rodents
Rodentia	Mus minutoides	Petružela et al. 2019	Evolutionary history of Pneumocystis fungi in their African rodent hosts
Rodentia	Mus musculus	Kučera et al. 1971	Pneumocystose
Rodentia	Mus musculus	Settnes and Lodal 1980	Prevalence of Pneumocystis carinii Delanoë & Delanoë, 1912 in rodents in Denmark
Rodentia	Mus musculus	Porter 1915	The occurrence of Pneumocystis carinii in mice in England
Rodentia	Mus musculus	Hayashimoto et al. 2014	Microbiological survey of mice (Mus musculus) purchased from commercial pet shops in Kanagawa and Tokyo, Japan
Rodentia	Mus musculus	Bellocq et al. 2018	Holobiont suture zones: Parasite evidence across the European house mouse hybrid zone

Rodentia	Mus triton	Petružela et al. 2019	Evolutionary history of Pneumocystis fungi in their African rodent hosts
Rodentia	Niviventer fulvescens	Latinne et al. 2021	Revisiting the Pneumocystis host specificity paradigm and transmission ecology in wild Southeast Asian rodents
Rodentia	Rattus argentiventer	Latinne et al. 2021	Revisiting the Pneumocystis host specificity paradigm and transmission ecology in wild Southeast Asian rodents
Rodentia	Rattus argentiventer	Latinne et al. 2018	Genetic diversity and evolution of Pneumocystis fungi infecting wild Southeast Asian murid rodents
Rodentia	Rattus exulans	Latinne et al. 2021	Revisiting the Pneumocystis host specificity paradigm and transmission ecology in wild Southeast Asian rodents
Rodentia	Rattus exulans	Latinne et al. 2018	Genetic diversity and evolution of Pneumocystis fungi infecting wild Southeast Asian murid rodents
Rodentia	Rattus nitidus	Latinne et al. 2021	Revisiting the Pneumocystis host specificity paradigm and transmission ecology in wild Southeast Asian rodents
Rodentia	Rattus nitidus	Latinne et al. 2018	Genetic diversity and evolution of Pneumocystis fungi infecting wild Southeast Asian murid rodents
Rodentia	Rattus norvegicus	Settnes and Lodal 1980	Prevalence of Pneumocystis carinii Delanoë & Delanoë, 1912 in rodents in Denmark
Rodentia	Rattus norvegicus	Yoshida and Ikai 1979	Pneumocystis carinii pneumonia: epidemiology in Japan and cyst concentration method
Rodentia	Rattus norvegicus	Palmer et al. 2000	Population structure of rat-derived Pneumocystis carinii in Danish wild rats
Rodentia	Rattus norvegicus	Chabé et al. 2010	Pneumocystis carinii and Pneumocystis wakefieldiae in wild Rattus norvegicus trapped in Thailand
Rodentia	Rattus norvegicus	Latinne et al. 2021	Revisiting the Pneumocystis host specificity paradigm and transmission ecology in wild Southeast Asian rodents
Rodentia	Rattus norvegicus	Latinne et al. 2018	Genetic diversity and evolution of Pneumocystis fungi infecting wild Southeast Asian murid rodents
Rodentia	Rattus rattus	Shimizu et al. 1985	Occurrence of Pneumocystis carinii in animals in Japan
Rodentia	Rattus rattus	Shimizu et al. 1985	Occurrence of Pneumocystis carinii in animals in Japan
Rodentia	Rattus sakeratensis	Latinne et al. 2021	Revisiting the Pneumocystis host specificity paradigm and transmission ecology in wild Southeast Asian rodents

Rodentia	Rattus sakeratensis	Latinne et al. 2018	Genetic diversity and evolution of Pneumocystis fungi infecting wild Southeast Asian murid rodents
Rodentia	Rattus tanezumi	Latinne et al. 2021	Revisiting the Pneumocystis host specificity paradigm and transmission ecology in wild Southeast Asian rodents
Rodentia	Rattus tanezumi	Latinne et al. 2018	Genetic diversity and evolution of Pneumocystis fungi infecting wild Southeast Asian murid rodents
Rodentia	Rattus tanezumi	Latinne et al. 2021	Revisiting the Pneumocystis host specificity paradigm and transmission ecology in wild Southeast Asian rodents
Rodentia	Rattus tanezumi	Latinne et al. 2018	Genetic diversity and evolution of Pneumocystis fungi infecting wild Southeast Asian murid rodents
Rodentia	Rattus tiomanicus	Latinne et al. 2021	Revisiting the Pneumocystis host specificity paradigm and transmission ecology in wild Southeast Asian rodents
Rodentia	Myocastor coypus	Danesi et al. 2016	Barcoding markers for Pneumocystis species in wildlife
Rodentia	Myocastor coypus	Danesi et al. 2018	Real-time PCR assay for screening Pneumocystis in free-living wild squirrels and river rats
Rodentia	Callosciurus finlaysonii	Danesi et al. 2018	Real-time PCR assay for screening Pneumocystis in free-living wild squirrels and river rats
