# Supplementary material for: Meta-Analysis and Systematic Literature Review of the Genus Pneumocystis in Pet, Farm, Zoo, and Wild Mammal Species
Source: J Fungi (Basel). 2023 Nov 4;9(11):1081. doi: 10.3390/jof9111081 (PMC10672670; doi:10.3390/jof9111081)
Supplement: Supplementary file 1 [file jof-09-01081-s001.zip › Table S3_v2.pdf]

Table S3. Additional statistical data on homogeneity and robustness of data.

1. Descriptive statistics including mean value, median value, standard deviation, standard error, confidence interval, minimum and maximum value.

1.1. Prevalence of the individual mammal orders.

	N	Mean value	Median value	Standard deviation	Standard error	95% confidence interval of the mean value		Minimum value	Maximum value
						Lower limit	Upper limit		
Artiodactyla	29	21.503	18.200	16.8565	3.1302	15.092	27.915	0.000	51.000
Carnivora	23	15.061	6.300	21.4303	4.4685	5.794	24.328	0.000	78.400
Chiroptera	23	30.974	30.000	14.7742	3.0806	24.585	37.363	0.000	56.500
Eulipotyphla	22	24.568	15.150	24.2666	5.1737	13.809	35.327	0.000	80.000
Lagomorpha	6	14.933	16.950	5.4441	2.2226	9.220	20.647	6.000	20.000
Perissodactyla	5	21.040	16.100	17.8748	7.9938	-1.154	43.234	5.000	45.500
Primates	3	22.300	33.300	19.3129	11.1503	-25.676	70.276	0.000	33.600
Rodentia	76	31.187	26.600	27.6183	3.1680	24.876	37.498	0.000	93.100

1.2. Prevalence achieved by the different methods.

	N	Mean value	Median value	Standard deviation	Standard error	95% confidence interval of the mean value		Minimum value	Maximum value
						Lower limit	Upper limit		
Cytology	78	11.856	6.700	14.7074	1.6653	8.540	15.172	0.000	70.000
Histology	4	31.275	32.650	6.1125	3.0562	21.549	41.001	22.700	37.100
ISH	16	19.994	15.200	20.7466	5.1867	8.939	31.049	0.000	68.000
IHC	3	49.167	50.000	13.2696	7.6612	16.203	82.130	35.500	62.000
PCR	86	38.808	35.400	23.2425	2.5063	33.825	43.791	0.000	93.100

1.3. Prevalence related to sample size.

	N	Mean value	Median value	Standard deviation	Standard error	95% confidence interval of the mean value		Minimum value	Maximum value
						Lower limit	Upper limit		
11-50 samples	121	25.598	20.000	23.3840	2.1258	21.389	29.807	0.000	90.900
51-100 samples	39	25.582	18.900	24.6290	3.9438	17.598	33.566	0.000	92.200
101-150 samples	9	15.889	8.000	13.7189	4.5730	5.344	26.434	0.900	35.500
151-200 samples	3	15.167	19.000	10.0162	5.7828	-9.715	40.048	3.800	22.700
201-250 samples	9	41.044	43.000	25.3819	8.4606	21.534	60.555	7.000	93.100
251-300 samples	1	9.000	9.000	.	.	.	.	9.000	9.000
301-350 samples	2	52.550	52.550	26.2337	18.5500	-183.150	288.250	34.000	71.100
351-400 samples	0								
401-450 samples	1	17.200	17.200					17.200	17.200
451-500 samples	1	33.600	33.600					33.600	33.600
501-550 samples	0								
551-600 samples	1	36.900	36.900					36.900	36.900

1.4. Prevalence of the different housing categories.

	N	Mean value	Median value	Standard deviation	Standard error	95% confidence interval of the mean value		Minimum value	Maximum value
						Lower limit	Upper limit		
Pet animals	23	14.939	9.000	16.5187	3.4444	7.796	22.082	0.000	68.000
Farm animals	27	21.681	18.000	17.4774	3.3635	14.768	28.595	0.000	51.000
Zoo animals	3	22.300	33.300	19.3129	11.1503	-25.676	70.276	0.000	33.600
Wild animals	133	28.864	24.000	24.9739	2.1655	24.580	33.148	0.000	93.100

1.5. Prevalence of wild animal species living in woodlands or not.

	N	Mean value	Median value	Standard deviation	Standard error	95% confidence interval of the mean value		Minimum value	Maximum value
						Lower limit	Upper limit		
Woodland	85	25.595	21.700	22.9754	2.4920	20.640	30.551	0.000	93.100
No woodland	41	36.310	29.400	28.9614	4.5230	27.168	45.451	0.000	92.200

1.6. Prevalence of wild animal species living in polar regions or not.

	N	Mean value	Median value	Standard deviation	Standard error	95% confidence interval of the mean value		Minimum value	Maximum value
						Lower limit	Upper limit		
Polar region	10	11.960	7.850	14.8784	4.7050	1.317	22.603	0.000	46.800
No polar region	116	30.558	24.500	25.6888	2.3851	25.833	35.282	0.000	93.100

1.7. Prevalence of wild animal species consuming different diets.

	N	Mean value	Median value	Standard deviation	Standard error	95% confidence interval of the mean value		Minimum value	Maximum value
						Lower limit	Upper limit		
Herbivore or frugivore	18	20.850	8.350	27.5304	6.4890	7.159	34.541	0.000	81.000
Insectivore	34	28.994	29.850	20.2148	3.4668	21.941	36.047	0.000	80.000
Carnivore	2	2.150	2.150	3.0406	2.1500	-25.168	29.468	0.000	4.300
Omnivore	49	36.496	31.600	28.3997	4.0571	28.339	44.653	0.000	93.100
Bloodsucking	3	24.233	20.000	10.3230	5.9600	-1.410	49.877	16.700	36.000
Herbivore or frugivore + insectivore	14	19.629	9.550	21.0406	5.6233	7.480	31.777	0.000	56.500
Herbivore or frugivore + carnivore	1	78.400	78.400	.	.	.	.	78.400	78.400
Insectivore + carnivore	2	10.650	10.650	3.3234	2.3500	-19.210	40.510	8.300	13.000

1.8. Prevalence of different lifestyles of wild animal species.

	N	Mean value	Median value	Standard deviation	Standard error	95% confidence interval of the mean value		Minimum value	Maximum value
						Lower limit	Upper limit		
Terrestrial	86	25.309	17.500	25.2136	2.7188	19.904	30.715	0.000	93.100
Semiaquatic	11	48.391	57.700	38.2714	11.5393	22.680	74.102	2.000	92.200
Arboreal	27	31.756	30.000	15.2789	2.9404	25.711	37.800	0.000	64.400
Terrestrial	86	25.309	17.500	25.2136	2.7188	19.904	30.715	0.000	93.100

1.9. Prevalence in animals with low- and high-grade infection levels.

	N	Mean value	Median value	Standard deviation	Standard error	95% confidence interval of the mean value		Minimum value	Maximum value
						Lower limit	Upper limit		
Low-grade infection levels	57	22.419	16.700	18.1463	2.4035	17.604	27.234	0.900	68.000
High-grade infection levels	30	31.673	33.650	17.0856	3.1194	25.293	38.053	3.600	68.000

2. Kolmogorov-Smirnov test for normal distribution.

Parameter	N	Absolute difference	p-value
Mammal order	187	0.269	< 0.001
Prevalence	187	0.133	< 0.001
Sample number	187	0.254	< 0.001
Sample size categorized	187	0.340	< 0.001
Method	187	0.296	< 0.001
Housing category	186	0.442	< 0.001
Woodland	129	0.434	< 0.001
Polar region	129	0.536	< 0.001
Diet	126	0.214	< 0.001
Lifestyle	127	0.421	< 0.001
Infection level	60	0.323	< 0.001

3. Levene's test for variance homogeneity.

Parameter	Levene's statistics	p-value
Prevalence of the individual mammal orders	3.851	< 0.001
Prevalence achieved by the different methods	4.846	< 0.001
Prevalence related to sample size	0.944	0.454
Prevalence of the different housing categories	3.566	0.015
Prevalence of wild animal species living in woodlands or not	5.307	0.023
Prevalence of wild animal species living in polar regions or not	4.908	0.029
Prevalence of wild animal species consuming different diets	2.954	0.010
Prevalence of different lifestyles of wild animal species	12.802	< 0.001
Prevalence in animals with low- and high-grade infection levels	0.175	0.677

4. Welch's test for robustness.

Parameter	Welch's statistics	p-value
Prevalence of the individual mammal orders	3.584	0.010
Prevalence achieved by the different methods	21.704	< 0.001
Prevalence related to sample size	---	---
Prevalence of the different housing categories	3.636	0.055
Prevalence of wild animal species living in woodlands or not	4.305	0.042
Prevalence of wild animal species living in polar regions or not	12.430	0.003
Prevalence of wild animal species consuming different diets	---	---
Prevalence of different lifestyles of wild animal species	2.697	0.087
Prevalence in animals with low- and high-grade infection levels	5.522	0.022
